# Anionic cascade reactions. One-pot assembly of (*Z*)-chloro-*exo*-methylenetetrahydrofurans from β-hydroxyketones

**DOI:** 10.3762/bjoc.9.148

**Published:** 2013-07-03

**Authors:** István E Markó, Florian T Schevenels

**Affiliations:** 1 Université catholique de Louvain, Laboratory of Organic and Medicinal chemistry, Place Louis Pasteur 1 bte L4.01.02, 1348 Louvain-la-Neuve; Belgium, Fax 0032-10-472788

**Keywords:** acetylene addition, dichloroethylene, dimerisation, dioxanes, tetrahydrofurans

## Abstract

The assembly of (*Z*)-chloro-*exo*-methylenetetrahydrofurans by an original and connective anionic cascade sequence is reported. Base-catalysed condensation of β-hydroxyketones with 1,1-dichloroethylene generates, in moderate to good yields, the corresponding (*Z*)-chloro-*exo*-methylenetetrahydrofurans. Acidic treatment of this motif leads to several unexpected dimers, possessing unique structural features.

## Introduction

**Scheme 1:**
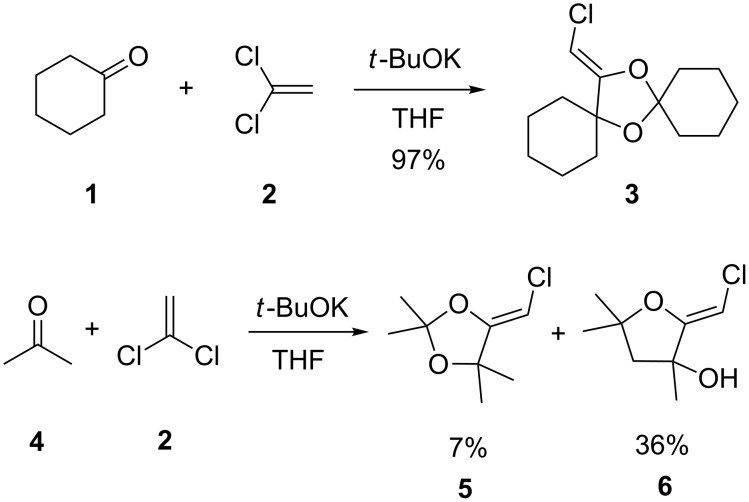
Formation of (*Z*)-chloro-*exo*-methyleneketals.

Recently, we have shown that simple ketones reacted with 1,1-dichloroethylene, in the presence of potassium *tert*-butoxide, to afford rare (*Z*)-chloro-*exo*-methyleneketals [1–3]. This unique transformation is particularly efficient in the case of six-membered ring ketones (Scheme 1). In an attempt to extend the scope of this cascade process to acyclic ketones, acetone was submitted to the usual reaction conditions. To our surprise, the expected adduct **5** was formed in only poor yields. The major product was isolated in 36% yield and its structure was unambiguously determined as the (*Z*)-chloro-*exo*-methylenetetrahydrofuran **6**.

**Scheme 2:**
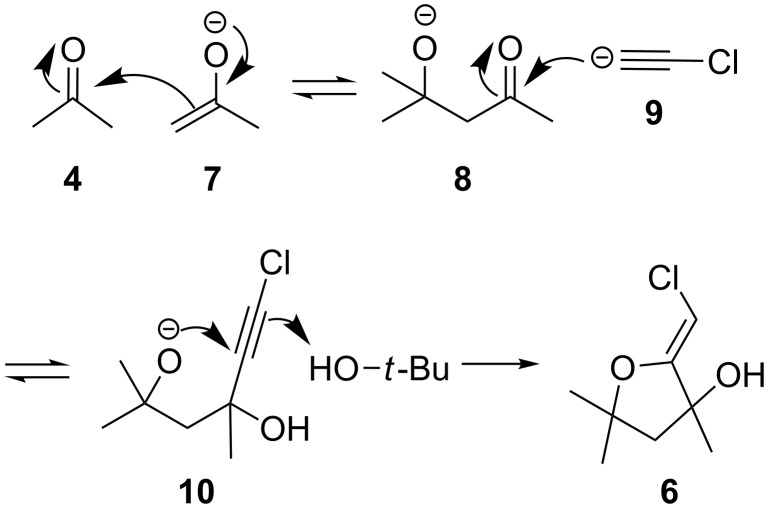
Mechanism of formation of (*Z*)-chloro-*exo*-methylenetetrahydrofurans.

The generation of **6** can be rationalized as depicted in Scheme 2. Under the basic conditions employed for the cascade reaction, 1,1-dichloroethylene is converted into the corresponding chloro-acetylene anion **9** [4–5]. This nucleophilic species adds rapidly, though reversibly, to acetone, leading ultimately to the formation of **5**. However, in this case, competitive aldol reaction appears to take place, delivering the adduct **8**. The subsequent addition of **9** then affords the intermediate **10**, which undergoes a 5-*exo*-*dig* cyclisation, ultimately yielding the observed (*Z*)-chloro-*exo*-methylenetetrahydrofuran **6**.

This structural motif constitutes the core of several biologically active compounds, including antimicrobial agents, fungicides and interesting competitive inhibitors of *S*-adenosyl-L-homocysteine (AdoHcy) hydrolase, one of the target enzymes extensively studied in the context of antiviral chemotherapy [6–9]. Despite their utility, only a limited number of multistep methods have been described for the preparation of this subunit [10–16]. The paucity of efficient synthetic approaches towards this family of compounds and the desire to assess the scope and limitations of this method prompted us to further investigate this transformation.

## Results and Discussion

**Scheme 3:**
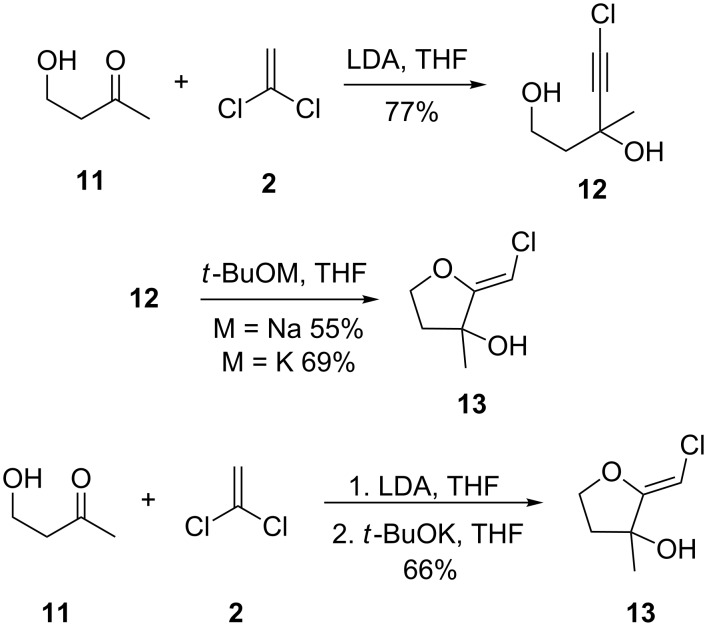
Stepwise formation of (*Z*)-chloro-*exo*-methylenetetrahydrofurans.

When 4-hydroxy-2-butanone (**11**) was treated with lithium chloroacetylide, generated in situ from dichloroethylene and LDA, the diol **12** was obtained in 77% yield. This adduct smoothly cyclized to **13** upon addition of sodium or potassium *tert*-butoxide, thereby supporting our mechanistic proposal. Repeating the process without isolation of the intermediate diol **12** proved to be even more efficient, affording the (*Z*)-chloro-*exo*-methylenetetrahydrofuran **13** in 66% overall yield (Scheme 3).

**Scheme 4:**
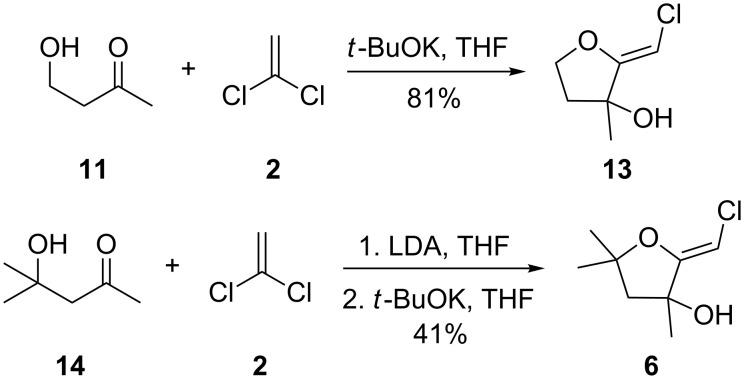
Optimized protocols to form (*Z*)-chloro-*exo*-methylenetetrahydrofurans.

It is interesting to note that, in the case of the substrate **11**, the adduct **13** could be isolated in an improved 81% yield when potassium *tert*-butoxide was employed as the sole base (Scheme 4). In stark contrast, applying this procedure to the keto-alcohol **14** resulted in a mediocre 13% yield of **6**, probably due to a rapid retro-aldol reaction of the derived, rather hindered, potassium alkoxide. Applying the LDA/KO*t*-Bu protocol improved the yield to 41%.

It thus transpires that good to excellent yields of (*Z*)-chloro-*exo*-methylenetetrahydrofurans could be obtained by the judicious choice of the bases employed to promote this cascade process. Having delineated some suitable conditions, the scope and limitations of this methodology were investigated [17–27]. Some selected examples are collected in Table 1.

**Table 1:** Preparation of (Z)-chloro-*exo*-methylenetetrahydrofurans.

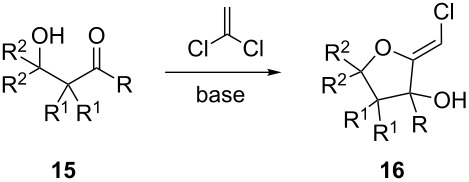			

Entry	Substrate	Product	Yield^a^

1	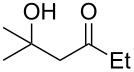 **17**	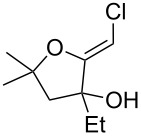 **25**	39%^b^
2	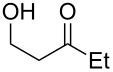 **18**	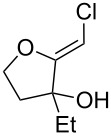 **26**	80%^c^
3	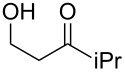 **19**	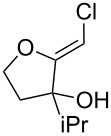 **27**	73%^c^
4	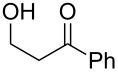 **20**	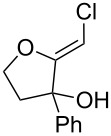 **28**	38%^c^ 47%^b^
5	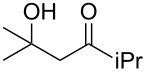 **21**	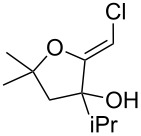 **29**	40%^b^
6	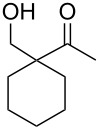 **22**	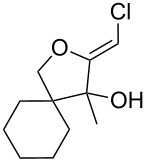 **30**	42%^c^
7	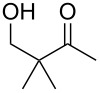 **23**	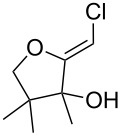 **31**	77%^c^
8	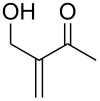 **24**	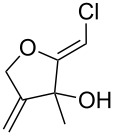 **32**	10%^c^ 61%^b^

^a^All yields are for pure, isolated products. ^b^In these cases, 2.2 equiv of C_2_H_2_Cl_2_ and 4.4 equiv of LDA were used in the first step, followed by 1 equiv of *t*-BuOK in the second step. ^c^For these substrates, 2.5 equiv of C_2_H_2_Cl_2_ and 5 equiv of *t*-BuOK were used to generate the acetylide.

As can be seen from Table 1, the reaction proves to be quite general and high-yielding for several primary alcohols (Table 1, entries 2, 3, 7 and 8). The yield was somewhat lower for two of them (Table 1, entries 4 and 6) due to rapid retro-aldol reactions and to enhanced steric hindrance, respectively. In the case of tertiary alcohols, the reaction proceeded with acceptable yields, especially in view of the one-pot nature of our procedure (Table 1, entries 1 and 5).

**Scheme 5:**
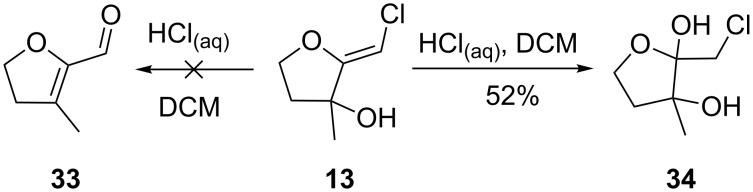
Hydration of (*Z*)-chloro-*exo*-methylenetetrahydrofurans.

Having access to a broad range of substituted (*Z*)-chloro-*exo*-methylenetetrahydrofurans, a brief survey of their reactivity was performed. Several reactions involving the vinyl chloride function proved unsuccessful [28–29]. Attempts to perform oxidative rearrangement and dehydration failed and functionalisation of the hydroxy group appeared difficult [30–31]. Initially, the adduct **13** was treated with aqueous hydrochloric acid, in the hope of generating the corresponding dihydrofuran carbaldehyde **33**. Instead, the diol **34** was obtained in 52% yield as a 1:1 mixture of diastereoisomers. It thus transpires that protonation and hydration of the *exo*-methylene double bond of **13** proceeded faster than the expected rearrangement of the tertiary allylic alcohol (Scheme 5).

**Scheme 6:**
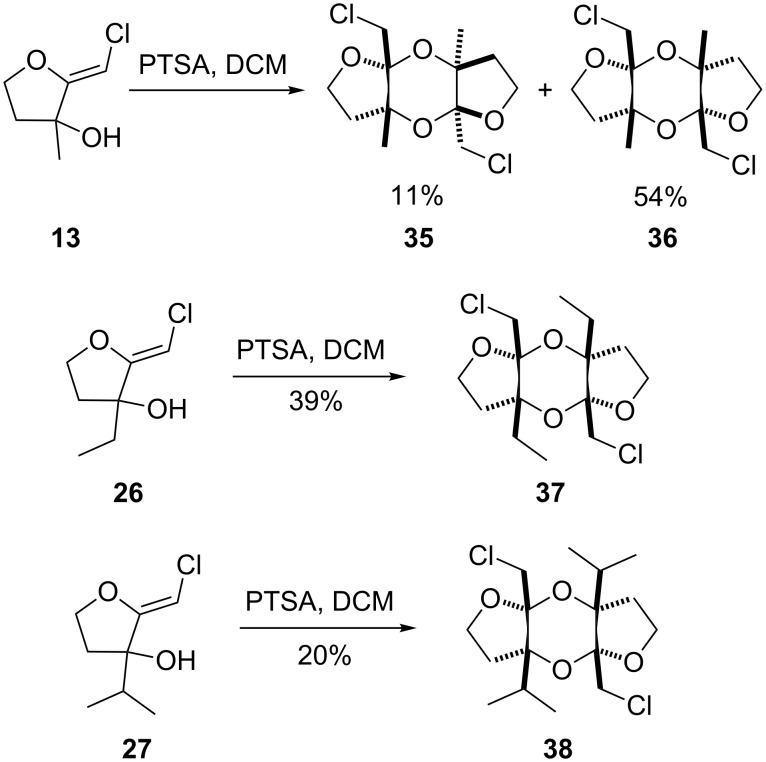
Formation of dioxanes.

**Figure 1:**
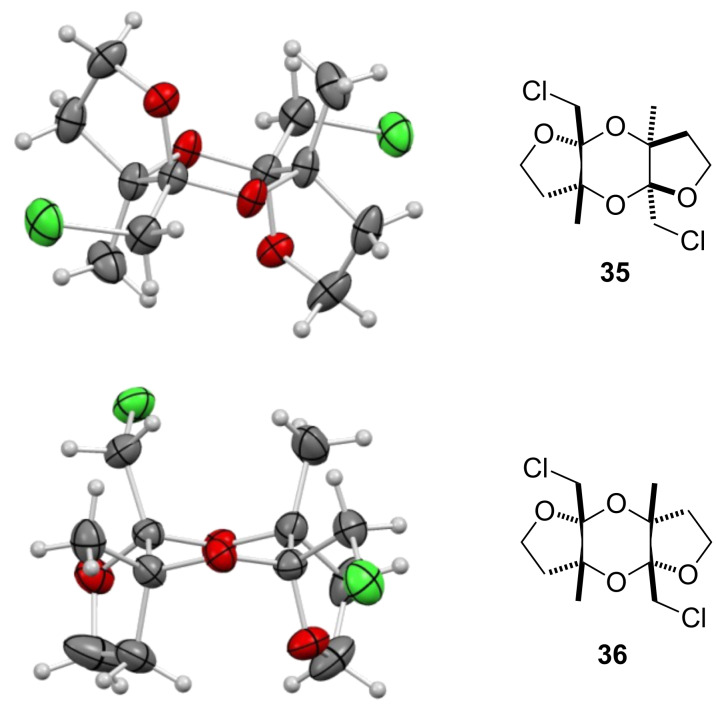
X-ray diffraction analysis of dioxanes **35** and **36**.

To promote such an acid-catalyzed rearrangement, the (*Z*)-chloro-*exo*-methylenetetrahydrofuran **13** was treated with a catalytic amount of *para*-toluenesulfonic acid. To our surprise, the *anti*-dioxane **35** and its *syn*-derivative **36** were obtained as a 1:5 mixture of diastereoisomers that could be separated (Scheme 6). Their structure was unambiguously established by single-crystal X-ray diffraction analysis, as shown in Figure 1. Increasing the steric hindrance at the tertiary alcohol site resulted in the exclusive formation of the *syn*-dioxanes **37** and **38** respectively, albeit at the expense of the yield [32–38].

**Scheme 7:**
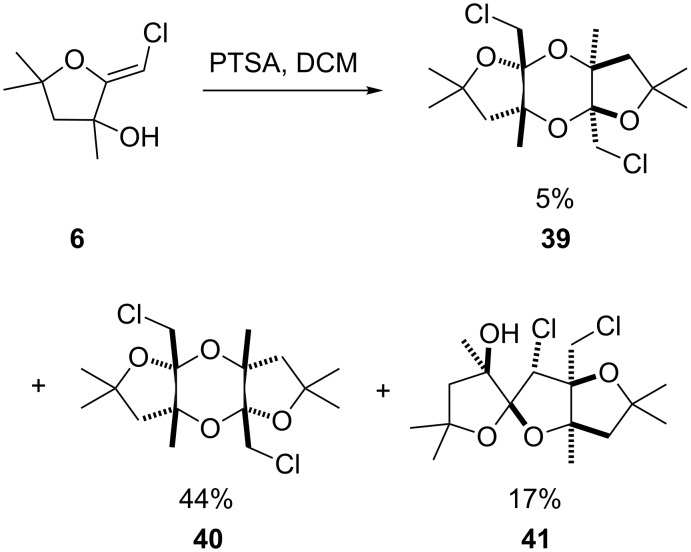
Formation of a new spirocyclic dimer.

When the dimethyl adduct **6** was submitted to the same conditions, a new product [39–45] **41** was formed besides the dioxanes **39** and **40** (Scheme 7). Its structure was unambiguously established as the spirocyclic dimer **41** by single-crystal X-ray diffraction analysis. It is noteworthy that a single diastereoisomer was generated in this transformation.

**Scheme 8:**
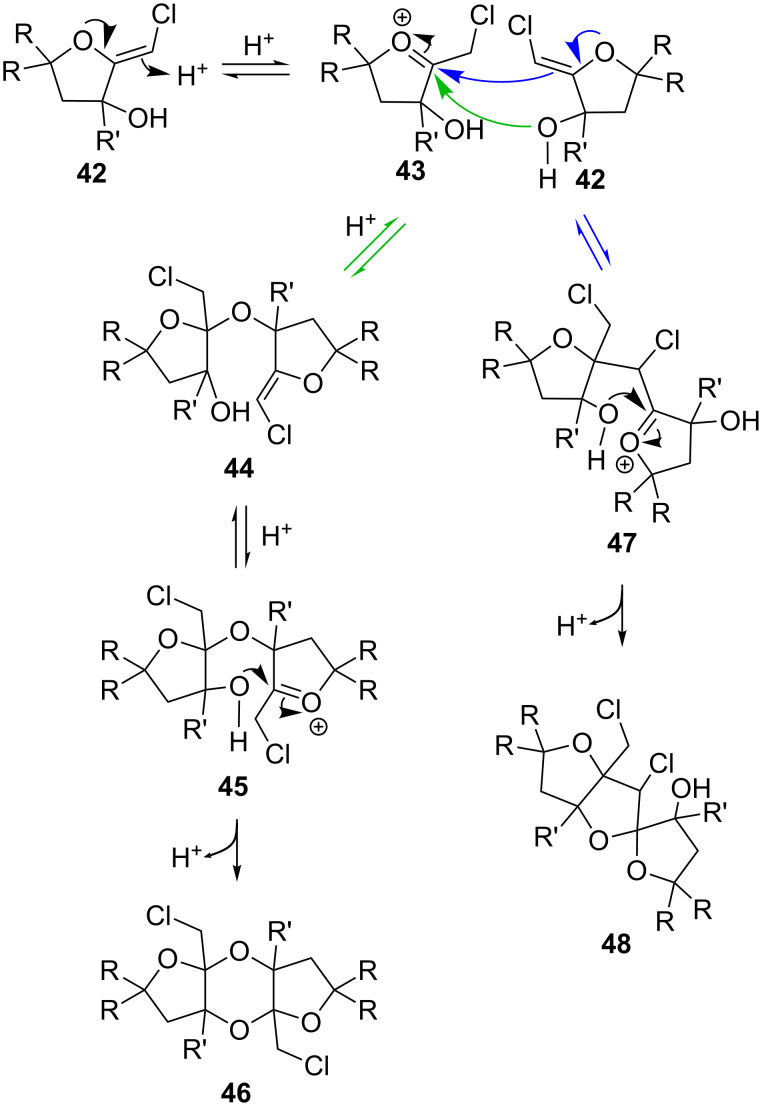
Mechanism leading to dioxanes and spirocycles.

The formation of these unique compounds can be rationalized as depicted in Scheme 8. Under acidic conditions, the (*Z*)-chloro-*exo*-methylenetetrahydrofuran **42** is converted into the oxonium cation **43**. Subsequent capture of this intermediate **43** by a second molecule of **42** can occur via two different pathways. The first one involves the addition of the enol ether function of **42** onto **43**, leading to the creation of a new C–C bond with concomitant generation of another oxonium species **47**. Intramolecular capture of this electrophile by the pendant hydroxy substituent then delivers the spirocyclic adduct **48**. Alternatively, reaction of the tertiary alcohol of **42** with the oxonium cation **43** affords the ketal **44**. Protonation of the enol ether function, followed by 6-*exo-trig* cyclization, completes this sequence of events and provides the dioxane derivative **46**. Interestingly, no reaction was observed when the spirocycle **41** and the dioxanes **35**, **36** and **39** were reacted under acidic conditions, indicating that the final step is not reversible.

**Scheme 9:**
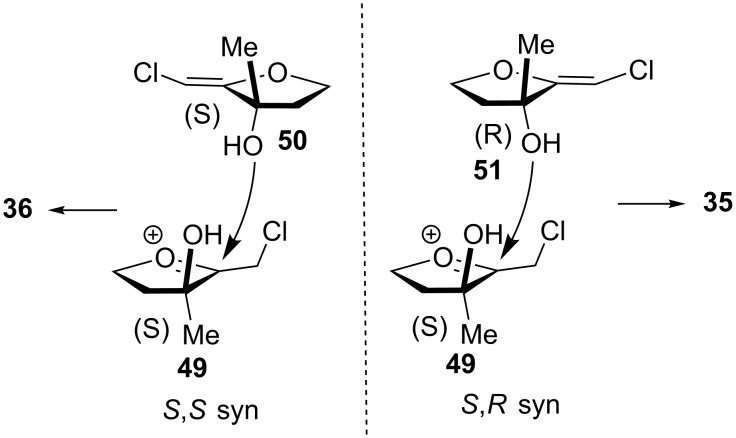
(*S*,*S)*-*syn* and (*S*,*R)*-*syn* approaches.

It is noteworthy that the dioxanes possess predominantly (**13**) or exclusively (**26** and **27**) the *syn–syn* relative stereochemistry at the ring junctions. This selectivity can be rationalized by an initial addition of the nucleophile *syn* to the alcohol function, as shown in Scheme 9. Interestingly, the homochiral (*S,S;R,R*)-dimer, leading to the *syn–syn* product, is preferred over the heterochiral (*S,R;R,S*)-adduct, affording the *syn–anti* isomer. Increasing the steric hindrance at the tertiary alcohol center of **49** leads to prohibitive steric repulsion during the heterodimer formation. A similar facial selectivity can explain the formation of **41** as a single diastereoisomer.

**Scheme 10:**
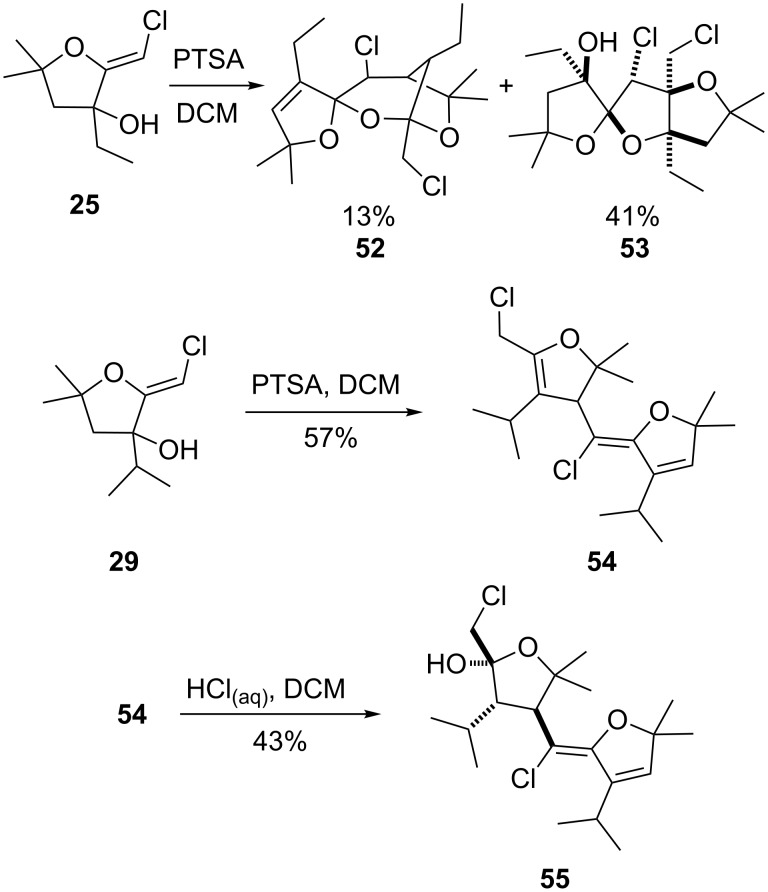
Formation of a bridged dimer and a triene.

When the adduct **25**, bearing a *gem*-dimethyl substituent was submitted to this transformation, no dioxane was observed. The major product proved to be the spirocyclic adduct **53**, accompanied by the bridged bis-ketal **52**, in 41% and 13% yield respectively (Scheme 10). Treatment of the isopropyl derivative **29** under the same acidic conditions provided the triene **54** in 57% yield. Addition of aqueous HCl generated the hemi-ketal **55** in 43% yield. The starting material was recovered in 52% yield.

**Figure 2:**
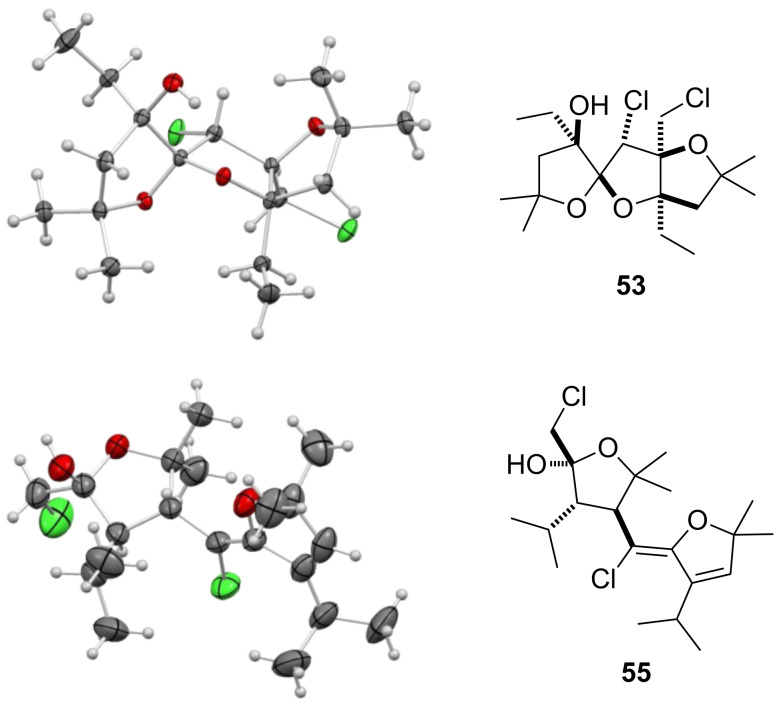
X-ray diffraction analysis of two new dimers.

The structures of compounds **53** and **55** were unambiguously established by single-crystal X-ray diffraction analysis (Figure 2).

**Scheme 11:**
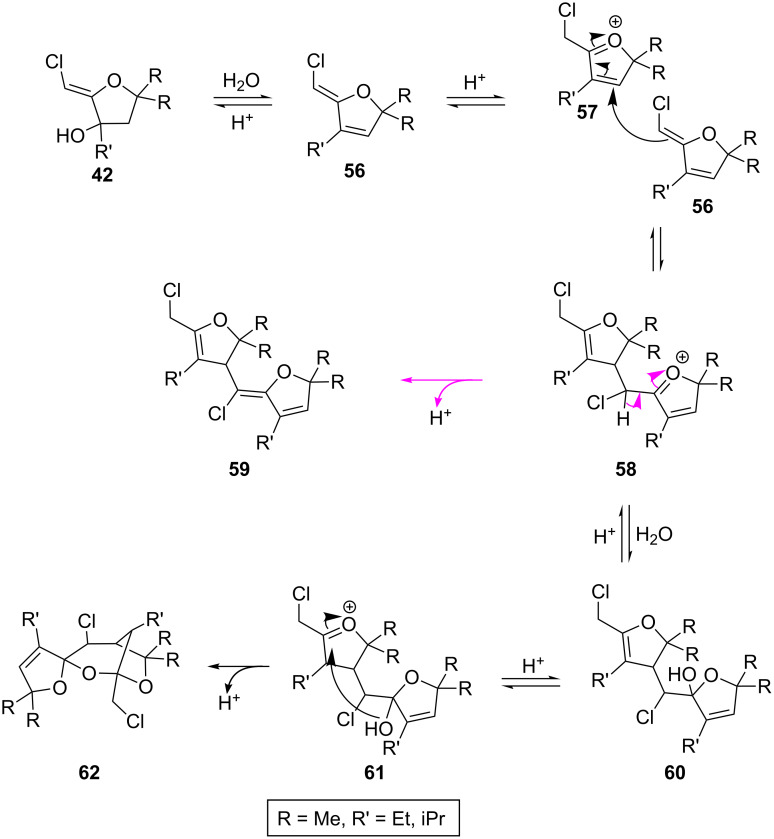
Mechanism leading to bridged and dienic dimers.

Once again, increasing the steric effect around the tertiary alcohol function of the (*Z*)-chloro-*exo*-methylenetetrahydrofurans has a profound influence on the fate of the condensation reaction. A plausible mechanistic rationale is provided in Scheme 11.

In the presence of the *gem*-dimethyl substituent, dehydration of **42** to afford **56** appears to proceed rapidly, probably owing to a release of steric strain. The loss of water occurs especially readily when R’ = Et or iPr and to a lesser extent when R’ = Me, indicating that steric decompression may indeed be operational in these cases. Protonation of diene **56** leads to the oxonium ion **57**, which undergoes a 1,4-addition of another **56** unit, affording the new cation **58**. At this stage, two different pathways can be followed. Either carbocation **58** can lose a proton, generating the observed triene **59**, or it may undergo addition of a water molecule, affording the hemiketal **60**. Addition of a proton to the vinyl ether function, followed by intramolecular capture by the hydroxy group, finally provides the unique bridged adduct **62**.

## Conclusion

In summary, a unique anionic cascade process, leading to the efficient and connective assembly of (*Z*)-chloro-*exo*-methylenetetrahydrofurans from β-hydroxyketones, has been uncovered and developed. The reactivity of this unusual motif has been briefly investigated, and dimers, possessing rather unusual structures, have been obtained. It is noteworthy that some of these dimeric products form the core of interesting biologically active compounds and of unique natural products.

## Experimental

### General procedure for the synthesis of chloromethylenefurans

To 40 mL of anhydrous THF, cooled to 0 °C, 1.2 mL (15.0 mmol, 2.5 equiv) of dichloroethylene and 3.37 g of potassium *tert*-butoxide were added. After 15 minutes, 700 mg (6.0 mmol, 1 equiv) of hydroxyketone in 1 mL of THF were added. After one hour, 20 mL of water was added and the mixture was neutralized with diluted sulfuric acid. The aqueous layer was extracted with 2 × 20 mL of DCM. The organic layer was dried, filtered and concentrated. The crude product was further purified by chromatography over silica gel providing 820 mg (4.6 mmol, 77%) of chloromethylenefuran **31** as a white solid. For full details, see Supporting Information File 1.

## Supporting Information

File 1 Experimental procedures and analytical data of cited compounds.
