# Supplementary material for: Anionic cascade reactions. One-pot assembly of (Z)-chloro-exo-methylenetetrahydrofurans from β-hydroxyketones
Source: Beilstein J Org Chem. 2013 Jul 3;9:1319–25. doi: 10.3762/bjoc.9.148 (PMC3701418; doi:10.3762/bjoc.9.148)

Supporting Information

for

Anionic cascade reactions. One-pot assembly of (*Z*)-chloro-*exo*-methylenetetrahydrofurans from β-hydroxyketones

István E. Markó* and Florian T. Schevenels

Address: Université catholique de Louvain, Laboratory of Organic and Medicinal chemistry, Place Louis Pasteur 1 bte L4.01.02, 1348 Louvain-la-Neuve; Belgium, Fax 0032-10-472788

Email: István E. Markó - istvan.marko@uclouvain.be

* Corresponding author

Experimental procedures and analytical data of cited compounds

Experimental section

General: ^{1}H and ^{13}C NMR spectra were recorded at room temperature with a Bruker Avance 300 instrument operating at a frequency of 300 MHz for ^{1}H and 75 MHz for ^{13}C. In cases of ambiguous assignments, spectra were recorded with a Bruker Avance 500. ^{1}H NMR spectra were recorded in $CDCl_3$ and referenced to $CHCl_3$ (δ = 7.26) as an internal standard. ^{13}C NMR spectra were referenced to the $CDCl_3$ (δ = 77.16 ppm) signal. Mass spectra were recorded using Varian Matt 44S and Finnigan-Matt TSQ-70 spectrometer. High-resolution mass data were obtained with a Kratos MS50TC instrument. Infrared spectra were recorded on a SHIMATZU-FTIR-8400S spectrometer and recorded in cm^{-1}. Elemental analyses were carried out at the University of Stuttgart, Germany or at the Chemical Research Center of Budapest, Hungary.

Tetramethyl-chloromethyleneketal 5

To a solution of 5.15 mL (70.0 mmol, 2 equiv) of acetone and 2.80 mL (35.0 mmol, 1 equiv) of vinylidene dichloride in 175 mL of THF, cooled to 0 °C, were added 7.85 g (70.0 mmol, 2 equiv) of potassium *tert*-butoxide. After 4 hours at room temperature, the mixture was concentrated to 30 mL. The solution was filtered over a pad of silica gel with 400 mL of

PE/EtOAc 3:1. After concentration, the orange oil was carefully heated under vacuum to collect, by sublimation, 400 mg (2.3 mmol, 7%) of **5** as a white solid. R_f = 0.82 (PE/EtOAc 5:1). ^{1}H NMR (300 MHz) δ = 4.95 (s, 1H), 1.50 (s, 6H), 1.41 (s, 6H) ppm. ^{13}C NMR (75 MHz) δ = 158.9, 112.6, 85.6, 81.8, 29.8, 28.1 ppm. MS (CI): *m/z* = 176.0. IR (neat): ν 2978, 1666. HRMS (EI): Calcd for $C_8H_{13}O_2Cl$; 176.0599. Found: 176.0602. mp 58 °C.

5-Chloro-3-methylpent-4-yne-1,3-diol (12)

To a solution of 6.2 mL (44.0 mmol, 4.4 equiv) of diisopropylamine in 50 mL of THF, cooled to 0 °C, were added 17.6 mL (44.0 mmol, 4.4 equiv, 2.5 M in hexane) of *n*-BuLi. The mixture was cooled to −78 °C before the addition of 1.8 mL (22.0 mmol, 2.2 equiv) of 1,1-dichloroethylene. The mixture was brought back to 0 °C and 0.86 mL (10.0 mmol, 1 equiv) of 4-hydroxy-2-butanone was added. After one hour at room temperature, 50 mL of aqueous NH_4Cl were poured in the reaction. The aqueous layer was extracted with 3 × 50 mL of dichloromethane. The organic phase was dried over magnesium sulfate, filtered and concentrated. Purification over silica gel provided 1.15 g (7.7 mmol, 77%) of **12** as a yellow oil. R_f = 0.17 (PE/EtOAc 3:2). ^{1}H NMR (300 MHz) δ = 4.37 (s, 1H), 4.11 (m, 1H), 3.88 (m, 1H), 3.23 (t, *J* = 4.6 Hz, 1H), 1.97 (m, 1H), 1.79 (dt, *J* = 11.7 Hz, *J* = 3.9 Hz, 1H), 1.50 (s, 3H) ppm. ^{13}C NMR (75 MHz) δ = 72.2, 69.5, 62.7, 60.6, 43.4, 30.6 ppm. MS (CI): *m/z* = 148.0. IR (neat): ν 3294, 2986, 2957, 2930, 2228. HRMS (CI): Calcd for $C_6H_{10}ClO_2$; 149.0369. Found: 149.0370.

(*Z*)-2-(Chloromethylene)-3-methyltetrahydrofuran-3-ol (13) from 12

To a solution of 450 mg (3.0 mmol, 1 equiv) of 5-chloro-3-methylpent-4-yne-1,3-diol (**12**) in 15 mL of THF, cooled to 0 °C, were added 340 mg (3.0 mmol, 1 equiv) of *t*-BuOK. After three hours, 10 mL of water were poured in the reaction and the pH was brought back to 7 by the addition of diluted sulfuric acid. The aqueous layer was extracted with 3 × 20 mL of dichloromethane. The organic phase was dried over magnesium sulfate, filtered and concentrated. Purification over silica gel provided 310 mg (2.1 mmol, 69%) of **13** as a yellowish oil. R_f = 0.33 (PE/EtOAc 2:1). ^{1}H NMR (300 MHz) δ = 5.27 (s, 1H), 4.33 (m, 1H), 4.26 (m, 1H), 2.18 (m, 1H), 2.06 (m, 1H), 1.98 (s, 1H), 1.49 (s, 3H) ppm. ^{13}C NMR (75 MHz) δ = 161.5, 88.0, 76.9, 69.2, 40.7, 25.4 ppm. MS (EI): *m/z* = 148.0. IR (neat): ν 3336, 2978, 2928, 2905, 1672. HRMS (EI): Calcd for $C_6H_9ClO_2$; 148.0286. Found: 148.0285.

(*Z*)-2-(Chloromethylene)-3-methyltetrahydrofuran-3-ol (13) using the LDA procedure

To a solution of 6.2 mL (44.0 mmol, 4.4 equiv) of diisopropylamine in 50 mL of THF, cooled to 0 °C, was added 17.6 mL (44.0 mmol, 4.4 equiv, 2.5 M in hexane) of *n*-BuLi. The mixture was cooled to −78 °C before the addition of 1.8 mL (22.0 mmol, 2.2 equiv) of 1,1-dichloroethylene. The mixture was brought back to 0 °C and 0.86 mL (10.0 mmol, 1 equiv) of 4-hydroxy-2-butanone were added. After one hour at room temperature, 50 mL of aqueous NH_4Cl were poured in the reaction. The aqueous layer was extracted with 3 × 50 mL of dichloromethane. The organic phase was dried over magnesium sulfate, filtered and concentrated. The crude mixture was dissolved in 50 mL of THF and cooled to 0 °C before the addition of 1.12 g (10.0 mmol, 1 equiv) of *t*-BuOK. After one hour, 30 mL of water was poured into the reaction and the pH was brought back to 7 by the addition of diluted sulfuric acid. The aqueous layer was extracted with 3 × 30 mL of dichloromethane. The organic phase was dried over magnesium sulfate, filtered and concentrated. Purification over silica gel provided 1.15 g (6.6 mmol, 66%) of **13** as a yellow oil.

(*Z*)-2-(Chloromethylene)-3-methyltetrahydrofuran-3-ol (13) using the *t*-BuOK procedure

To a solution of 2.96 mL (37.0 mmol, 3.7 equiv) of 1,1-dichloroethylene in 65 mL of THF, cooled to 0 °C, were added 8.30 g (74.0 mmol, 7.4 equiv) of *t*-BuOK. After 15 minutes, 0.86 mL (10.0 mmol, 1 equiv) of 4-hydroxy-2-butanone were added to the reaction. After three hours, 30 mL of water was poured into the reaction and the pH was brought back to 7 by the addition of diluted sulfuric acid. The aqueous layer was extracted with 3 × 30 mL of dichloromethane. The organic phase was dried over magnesium sulfate, filtered and concentrated. Purification over silica gel provided 1.20 g (8.1 mmol, 81%) of **13** as a yellow oil.

(*Z*)-2-(Chloromethylene)-3,5,5-trimethyltetrahydrofuran-3-ol (6)

To a solution of 6.2 mL (44.0 mmol, 4.4 equiv) of diisopropylamine in 50 mL of THF, cooled to 0 °C, was added 17.6 mL (44.0 mmol, 4.4 equiv, 2.5 M in hexane) of *n*-BuLi. The mixture was cooled to −78 °C before addition of 1.8 mL (22.0 mmol, 2.2 equiv) of 1,1-dichloroethylene. The mixture was brought back to 0 °C and 1.25 mL (10.0 mmol, 1 equiv) of 4-hydroxy-4-methyl-2-pentanone was added. After one hour, 50 mL of aqueous NH_4Cl was poured into the reaction. The aqueous layer was extracted with 3 × 50 mL of dichloromethane. The organic phase was dried over magnesium sulfate, filtered and concentrated. The crude mixture was dissolved in 50 mL of THF and cooled to 0 °C before the addition of 1.12 g (10.0 mmol, 1 equiv) of *t*-BuOK. After one hour, 30 mL of water was poured into the reaction and the pH was brought back to 7 by addition of diluted sulfuric acid. The aqueous layer was extracted with 3 × 30 mL of dichloromethane. The organic phase was dried over magnesium sulfate, filtered and concentrated. Purification over silica gel provided 720 mg (4.1 mmol, 41%) of **6** as a yellow oil. R_f = 0.44 (PE/EtOAc 2:1). ^{1}H NMR (300 MHz) δ = 5.20 (s, 1H), 2.17 (d, *J* = 13.3 Hz, 1H), 1.97 (d, *J* = 13.4 Hz, 1H), 1.92 (s, 1H), 1.51 (s, 3H), 1.49 (s, 3H), 1.41 (s, 3H) ppm. ^{13}C NMR (75 MHz) δ = 162.1, 87.6, 85.6, 78.8, 52.3, 29.4, 28.4, 27.7 ppm. MS (EI): *m*/*z* = 176.1. IR (neat): ν 3377, 2976, 2935, 1666. HRMS (EI): Calcd for $C_8H_{13}ClO_2$; 176.0599. Found: 176.0603.

5-Hydroxy-5-methylhexan-3-one (17)

To a solution of 6.2 mL (44.0 mmol, 1.1 equiv) of diisopropylamine in 110 mL of diethylether, cooled to 0 °C, was added 17.6 mL (44.0 mmol, 1.1 equiv, 2.5 M in hexane) of *n*-BuLi. After 30 minutes, the mixture was cooled to −78 °C before the dropwise addition of 2.1 mL (40.0 mmol, 1 equiv) of 2-butanone in 10 mL of diethyl ether. After one hour, 3.6 mL (48.0 mmol, 1.2 equiv) of acetone was added dropwise. The mixture was stirred for three more hours. Then, 50 mL of aqueous NH_4Cl was poured into the reaction and the system was brought back to room temperature. The aqueous layer was extracted with 2 × 40 mL of diethyl ether. The organic phase was dried over magnesium sulfate, filtered and concentrated. Purification over silica gel provided 1.20 g (9.2 mmol, 23%) of **17** as a colorless oil. Data matched with the literature [1]. R_f = 0.37 (PE/EtOAc 2:1). ^{1}H NMR (300 MHz) δ = 3.89 (s, 1H), 2.58 (s, 2H), 2.43 (q, *J* = 7.3 Hz, 2H), 1.23 (s, 6H), 1.04 (t, *J* = 7.3 Hz, 3H) ppm. ^{13}C NMR (75 MHz) δ = 213.7, 69.7, 52.7, 37.8, 29.4, 7.5 ppm.

Methyl 2-(2-ethyl-1,3-dioxolan-2-yl)acetate (63)

In a 250 mL flask equipped with a Dean–Stark apparatus, 3.76 mL (30.0 mmol, 1 equiv) of methyl 3-oxopentanoate, 3.3 mL (60.0 mmol, 2 equiv) of ethylene glycol and 57 mg (0.01 mmol, 0.01 equiv) of PTSA were dissolved in 150 mL of benzene. After 18 hours under reflux, the reaction was brought back to room temperature before the addition of 100 mL of aqueous Na_2CO_3. The organic phase was washed with 70 mL of water, dried over magnesium sulfate, filtered and concentrated to furnish 4.44 g (25.6 mmol, 85%) of methyl 2-(2-ethyl-1,3-dioxolan-2-yl)acetate (**63**) as a colorless oil. Data matched with the literature [2]. R_f = 0.60 (PE/EtOAc 2:1). ^{1}H NMR (300 MHz) δ = 3.97 (m, 4H), 3.68 (s, 3H), 2.65 (s, 2H), 1.81 (q, J = 7.4 Hz, 2H), 0.93 (t, J = 7.4 Hz, 3H) ppm. ^{13}C NMR (75 MHz) δ = 170.2, 109.8, 65.3, 51.9, 42.2, 30.7, 7.9 ppm.

2-(2-Ethyl-1,3-dioxolan-2-yl)ethanol (64)

To a solution of 1.20 g (31.6 mmol, 1.25 equiv) of $LiAlH_4$ in 150 mL of diethyl ether, cooled to 0 °C, were added dropwise 4.40 g (25.3 mmol, 1 equiv) of methyl 2-(2-ethyl-1,3-dioxolan-2-yl)acetate (**63**) in 40 mL of diethyl ether. After 18 hours at room temperature, 1.2 mL of water, 3.8 mL of aqueous NaOH and 16 g of Na_2SO_4 were added. After 10 minutes, the solid was filtered and washed with 20 mL of diethyl ether. After concentration, 3.48 g (23.8 mmol, 94%) of 2-(2-ethyl-1,3-dioxolan-2-yl)ethanol (**64**) were obtained as a colorless oil. Data matched with the literature [2]. R_f = 0.22 (PE/EtOAc 2:1). ^{1}H NMR (300 MHz) δ = 3.98 (m, 4H), 3.73 (q, J = 5.6 Hz, 2H), 2.83 (t, J = 5.6 Hz, 1H), 1.91 (t, J = 5.6 Hz, 2H), 1.66 (q, J = 7.5 Hz, 2H), 0.90 (t, J = 7.5 Hz, 3H) ppm. ^{13}C NMR (75 MHz) δ = 112.7, 65.0, 59.0, 37.7, 30.0, 8.2 ppm.

1-Hydroxypentan-3-one (18)

To a suspension of 27.1 g of silica in 230 mL of dichloromethane was added 4.2 mL (4.7 mmol, 0.2 equiv, 10% in water) of oxalic acid. Once the aqueous layer vanished, 3.40 g (23.3 mmol, 1 equiv) of 2-(2-ethyl-1,3-dioxolan-2-yl)ethanol (**64**) in 7 mL of dichloromethane was added to the reaction. After 5 hours, 800 mg of $NaHCO_3$ was added and the mixture was filtered. The solid was washed several times with diethyl ether. After concentration, 1.64 g (16.1 mmol, 69%) of **18** was obtained as a colorless oil. Data matched with the literature [2]. R_f = 0.22 (PE/EtOAc 2:1). ^{1}H NMR (300 MHz) δ = 3.85 (t, J = 5.5 Hz, 2H), 2.67 (t, J = 5.4 Hz, 3H), 2.47 (q, J = 7.3 Hz, 2H), 1.06 (t, J = 7.3 Hz, 3H) ppm. ^{13}C NMR (75 MHz) δ = 212.5, 58.0, 44.0, 36.6, 7.7 ppm.

Methyl 2-(2-isopropyl-1,3-dioxolan-2-yl)acetate (65)

In a 250 mL flask equipped with a Dean–Stark apparatus, 4.28 mL (30.0 mmol, 1 equiv) of methyl 4-methyl-3-oxopentanoate, 3.3 mL (60.0 mmol, 2 equiv) of ethylene glycol and 57 mg (0.01 mmol, 0.01 equiv) of PTSA were dissolved in 150 mL of benzene. After 18 hours under reflux, the reaction was brought back to room temperature before the addition of 100 mL of aqueous Na_2CO_3. The organic phase was washed with 70 mL of water, dried over magnesium sulfate, filtered and concentrated to furnish 4.23 g (22.7 mmol, 75%) of methyl 2-(2-isopropyl-1,3-dioxolan-2-yl)acetate (**65**) as a colorless oil. Data matched with the literature [3]. R_f= 0.69 (PE/EtOAc 2:1). ^{1}H NMR (300 MHz) δ = 3.97 (m, 4H), 3.66 (s, 3H), 2.66 (s, 2H), 2.08 (hept, J = 6.8 Hz, 1H), 0.94 (d, J = 6.9 Hz, 6H) ppm. ^{13}C NMR (75 MHz) δ = 170.5, 111.8, 65.6, 51.8, 40.1, 35.5, 17.1 ppm.

2-(2-Isopropyl-1,3-dioxolan-2-yl)ethanol (66)

To a solution of 1.06 g (27.9 mmol, 1.25 equiv) of $LiAlH_4$ in 130 mL of diethyl ether, cooled to 0 °C, was added dropwise 4.20 g (22.3 mmol, 1 equiv) of methyl 2-(2-isopropyl-1,3-dioxolan-2-yl)acetate (**65**) in 30 mL of diethyl ether. After 18 hours at room temperature, 1.2mL of water, 3.8 mL of aqueous NaOH and 16 g of Na_2SO_4 were respectively added. After 10 minutes, the solid was filtered and washed with 20 mL of diethyl ether. After concentration, 3.50 g (21.8 mmol, 98%) of 2-(2-isopropyl-1,3-dioxolan-2-yl)ethanol (**66**) were obtained as a colorless oil. Data matched with the literature [2]. R_f = 0.28 (PE/EtOAc 2:1). ^{1}H NMR (300 MHz) δ = 4.00 (m, 4H), 3.74 (q, J = 5.5 Hz, 2H), 2.84 (t, J = 5.6 Hz, 1H), 1.97 (m, 1H), 1.91 (m, 2H), 0.93 (d, J = 6.9 Hz, 6H) ppm. ^{13}C NMR (75 MHz) δ = 114.8, 65.2, 58.8, 34.4, 34.4, 17.3 ppm.

1-Hydroxy-4-methylpentan-3-one (19)

To a suspension of 27.0 g of silica in 230 mL of dichloromethane was added 4.2 mL (4.7 mmol, 0.2 equiv, 10% in water) of oxalic acid. Once the aqueous layer vanished, 3.70 g (23.1 mmol, 1 equiv) of 2-(2-isopropyl-1,3-dioxolan-2-yl)ethanol (**66**) in 7 mL of dichloromethane were added to the reaction. After 5 hours, 800 mg of $NaHCO_3$ was added and the mixture was filtered. The solid was washed several times with diethyl ether. After concentration, 1.96 g (16.9 mmol, 73%) of **19** was obtained as a colorless oil. Data matched with the literature [2]. R_f = 0.22 (PE/EtOAc 2:1). ^{1}H NMR (300 MHz) δ = 3.84 (t, J = 5.4 Hz, 2H), 2.81 (s, 1H), 2.71 (t, J = 5.4 Hz, 2H), 2.60 (hept, J = 6.9 Hz, 1H), 1.11 (d, J = 6.9 Hz, 6H) ppm. ^{13}C NMR (75 MHz) δ = 215.9, 58.1, 42.0, 41.4, 18.2 ppm.

Ethyl 2-(2-phenyl-1,3-dioxolan-2-yl)acetate (67)

In a 250 mL flask equipped with a Dean–Stark apparatus, 3.76 mL (30.0 mmol, 1 equiv) of ethyl 3-oxo-3-phenylpropanoate, 3.3 mL (60.0 mmol, 2 equiv) of ethylene glycol and 57 mg (0.01 mmol, 0.01 equiv) of PTSA were dissolved in 150 mL of benzene. After 18 hours under reflux, the reaction was brought back to room temperature before the addition of 100 mL of aqueous Na_2CO_3. The organic phase was washed with 70 mL of water, dried over magnesium sulfate, filtered and concentrated. Purification over silica gel provided 3.27 g (13.8 mmol, 46%) of ethyl 2-(2-phenyl-1,3-dioxolan-2-yl)acetate (**67**) as a colorless oil. Data matched with the literature [4]. R_f = 0.63 (PE/EtOAc 2:1). ^{1}H NMR (300 MHz) δ = 7.50 (m, 2H), 7.34 (m, 3H), 4.07 (m, 4H), 3.82 (m, 2H), 2.96 (s, 2H), 1.15 (t, J = 7.1 Hz, 3H) ppm. ^{13}C NMR (75 MHz) δ = 168.7, 141.5, 128.4, 128.2, 125.7, 108.0, 65.0, 60.6, 46.0, 14.2 ppm.

2-(2-Phenyl-1,3-dioxolan-2-yl)ethanol (68)

To a solution of 0.66 g (17.2 mmol, 1.25 equiv) of $LiAlH_4$ in 80 mL of diethyl ether, cooled to 0 °C, were added dropwise 3.25 g (13.8 mmol, 1 equiv) of ethyl 2-(2-phenyl-1,3-dioxolan-2-yl)acetate (**67**) in 20 mL of diethyl ether. After 18 hours at room temperature, 0.7 mL of water, 2.0 mL of aqueous NaOH and 9 g of Na_2SO_4 were added. After 10 minutes, the solid was filtered and washed with 20 mL of diethyl ether. After concentration, 2.54 g (13.1 mmol, 95%) of 2-(2-phenyl-1,3-dioxolan-2-yl)ethanol (**68**) was obtained as a colorless oil. Data matched with the literature [5]. R_f = 0.30 (PE/EtOAc 2:1). ^{1}H NMR (300 MHz) δ = 7.45 (m, 2H), 7.31 (m, 3H), 4.07 (m, 2H), 3.78 (m, 2H), 3.73 (q, J = 5.3 Hz, 2H), 2.87 (t, J = 5.7 Hz, 1H), 2.17 (t, J = 5.3 Hz, 2H) ppm. ^{13}C NMR (75 MHz) δ = 141.9, 128.4, 128.3, 125.6, 111.0, 64.4, 58.7, 42.0 ppm.

3-Hydroxy-1-phenylpropan-1-one (20)

To a suspension of 15.0 g of silica in 130 mL of dichloromethane was added 2.3 mL (2.6 mmol, 0.2 equiv, 10% in water) of oxalic acid. Once the aqueous layer had vanished, 2.50 g (12.9 mmol, 1 equiv) of 2-(2-phenyl-1,3-dioxolan-2-yl)ethanol (**68**) in 4 mL of dichloromethane was added to the reaction. After 5 hours, 440 mg of $NaHCO_3$ was added and the mixture was filtered. The solid was washed several times with diethyl ether. After concentration, 1.35 g (9.0 mmol, 70%) of **20** was obtained as a colorless oil. Data matched with the literature [6]. R_f = 0.22 (PE/EtOAc 2:1). ^{1}H NMR (300 MHz) δ = 7.96 (m, 2H), 7.58 (m, 1H), 7.47 (m, 2H), 4.03 (t, J = 5.3 Hz, 2H), 3.23 (t, J = 5.7 Hz, 2H), 2.93 (s, 1 H) ppm. ^{13}C NMR (75 MHz) δ = 200.6, 136.7, 133.7, 128.8, 128.2, 58.2, 40.5 ppm.

5-Hydroxy-2,5-dimethylhexan-3-one (21)

To a solution of 3.1 mL (22.0 mmol, 1.1 equiv) of diisopropylamine in 60 mL of diethyl ether, cooled to 0 °C, were added 8.8 mL (22.0 mmol, 1.1 equiv, 2.5 M in hexane) of *n*-BuLi. After 30 minutes, the mixture was cooled to −78 °C before a dropwise addition of 2.2 mL (20.0 mmol, 1 equiv) of 3-methyl-2-butanone. After one hour, 1.8 mL (24.0 mmol, 1.2 equiv) of acetone were added dropwise. The mixture was stirred for three more hours. Then, 20 mL of aqueous NH_4Cl was poured into the reaction and the system was brought back to room temperature. The aqueous layer was extracted with 2 × 20 mL of diethyl ether. The organic phase was dried over magnesium sulfate, filtered and concentrated. Purification over silica gel provided 1.97 g (13.7 mmol, 68%) of **21** as a colorless oil. Data matched with the literature [7]. R_f = 0.46 (PE/EtOAc 2:1). ^{1}H NMR (300 MHz) δ = 4.09 (s, 1H), 2.60 (s, 2H), 2.54 (hept, J = 6.9 Hz, 1H), 1.21 (s, 6H), 1.07 (d, J = 6.9 Hz, 6H) ppm. ^{13}C NMR (75 MHz) δ = 217.2, 69.7, 50.6, 42.2, 29.4, 17.8 ppm.

1-(1-(Hydroxymethyl)cyclohexyl)ethanone (22)

To a solution of 1.85 g (14.7 mmol, 1 equiv) of cyclohexylmethylketone in 2.3 mL (29.3 mmol, 2 equiv) of TFA was added 0.44 g (14.7 mmol, 1 equiv) of paraformaldehyde. The mixture was heated to 90 °C for 10 hours. The mixture was cooled to room temperature. Then, 15 mL of aqueous $NaHCO_3$ was carefully added to the reaction. The aqueous layer was extracted with 3 × 10 mL of dichloromethane. The organic phase was dried over magnesium sulfate, filtered and concentrated. Purification over silica gel provided 280 mg (1.8 mmol, 12%) of **22** as an orange oil. Data matched with the literature [8,9]. R_f = 0.27 (PE/EtOAc 2:1). ^{1}H NMR (300 MHz) δ = 3.66 (d, J = 4.1 Hz, 2H), 2.18 (s, 3H), 2.05 (broad, 1H), 1.90 (m, 2H), 1.45 (m, 8H) ppm. ^{13}C NMR (75 MHz) δ = 214.5, 67.3, 53.8, 29.8, 26.1, 26.0, 22.3 ppm.

4-Hydroxy-3,3-dimethylbutan-2-one (23)

To a solution of 5.4 mL (50.0 mmol, 1 equiv) of 3-methyl-2-butanone in 7.7 mL (100.0 mmol, 2 equiv) of TFA were added 1.50 g (50.0 mmol, 1 equiv) of paraformaldehyde. The mixture was heated to 90 °C for 10 hours. The mixture was cooled to room temperature. Then, 30 mL of aqueous $NaHCO_3$ were carefully added in the reaction. The aqueous layer was extracted with 3 × 20 mL of dichloromethane. The organic phase was dried over magnesium sulfate, filtered and concentrated. Purification over silica gel provided 760 mg (6.5 mmol, 13%) of **23** as a yellow oil. Data matched with the literature [10]. R_f = 0.25 (PE/EtOAc 2:1). ^{1}H NMR (300 MHz) δ = 3.53 (s, 2H), 2.49 (broad, 1H), 2.14 (s, 3H), 1.14 (s, 6 H) ppm. ^{13}C NMR (75 MHz) δ = 215.4, 69.5, 49.3, 25.7, 21.7 ppm.

3-(Hydroxymethyl)but-3-en-2-one (24)

To a solution of 3.3 mL (40.0 mmol, 4.4 equiv) of but-3-ene-2-one and 3.0 mL (48.0 mmol, 1.2 equiv, 37% in water) of formaldehyde in 20 mL of THF, cold to 0 °C, was added 450 mg (4.0 mmol, 0.1 equiv) of DABCO. The mixture was brought back to room temperature and stirred for 20 hours before the addition of 20 mL of brine. The aqueous layer was extracted with 3 × 20 mL of diethyl ether. The organic phase was dried over magnesium sulfate, filtered and concentrated. Purification over silica gel provided 1.72 g (17.2 mmol, 43%) of 3-(hydroxymethyl)but-3-en-2-one **24** as a colorless oil. Data matched with the literature [11]. R_f = 0.16 (PE/EtOAc 2:1). ^{1}H NMR (300 MHz) δ = 6.10 (s, 1H), 6.03 (t, *J* = 1.4 Hz, 1H), 4.28 (s, 2H), 2,70 (broad, 1H), 2.34 (s, 3H) ppm. ^{13}C NMR (75 MHz) δ = 200.5, 147.3, 126.2, 62.2, 26.0 ppm.

(*Z*)-2-(Chloromethylene)-3-ethyl-5,5-dimethyltetrahydrofuran-3-ol (25)

To a solution of 4.6 mL (32.6 mmol, 4.4 equiv) of diisopropylamine in 30 mL of THF, cooled to 0 °C, were added 13.1 mL (32.6 mmol, 4.4 equiv, 2.5 M in hexane) of *n*-BuLi. The mixture was cooled to −78 °C before the addition of 1.3 mL (16.3 mmol, 2.2 equiv) of 1,1-dichloroethylene. The mixture was brought back to 0 °C and 965 mg (7.4 mmol, 1 equiv) of hydroxyketone **17** were added. After one hour, 30 mL of aqueous NH_4Cl were poured into the reaction. The aqueous layer was extracted with 3 × 20 mL of diethyl ether. The organic phase was dried over magnesium sulfate, filtered and concentrated. The crude mixture was dissolved in 30 mL of THF and cooled to 0 °C before the addition of 832 mg (7.4 mmol, 1 equiv) of *t*-BuOK. After one hour, 20 mL of water was poured into the reaction and the pH was brought back to 7 by the addition of diluted sulfuric acid. The aqueous layer was extracted with 2 × 20 mL of dichloromethane. The organic phase was dried over magnesium sulfate, filtered and concentrated. Purification over silica gel provided 550 mg (2.9 mmol, 39%) of **25** as a yellow oil. R_f = 0.57 (PE/EtOAc 2:1). ^{1}H NMR (300 MHz) δ = 5.16 (s, 1H), 2.08 (d, *J* = 13.4 Hz, 1H), 1.97 (d, *J* = 13.4 Hz, 1H), 1.78 (m, 1H), 1,66 (m, 1H), 1.60 (s, 1H), 1.52 (s, 3H), 1.43 (s, 3H), 0.99 (t, *J* = 7.4 Hz, 3H) ppm. ^{13}C NMR (75 MHz) δ = 161.8, 87.9, 85.6, 82.0, 49.3, 33.3, 29.4, 28.6, 8.9 ppm. MS (CI): *m*/*z* = 190.1. IR (neat): ν 3429, 2974, 2937, 2881, 1666. HRMS (CI): Calcd for $C_9H_{16}ClO_2$; 191.0839. Found: 191.0836.

(*Z*)-2-(Chloromethylene)-3-ethyltetrahydrofuran-3-ol (26)

To a solution of 1.0 mL (12.5 mmol, 2.5 equiv) of 1,1-dichloroethylene in 30 mL of THF, cooled to 0 °C, was added 2.81 g (25.0 mmol, 5 equiv) of *t*-BuOK. After 15 minutes, 510 mg (5.0 mmol, 1 equiv) of hydroxyketone **18** was added to the reaction. After one hour, 20 mL of water was poured in the reaction and the pH was brought back to 7 by the addition of diluted sulfuric acid. The aqueous layer was extracted with 2 × 30 mL of dichloromethane. The organic phase was dried over magnesium sulfate, filtered and concentrated. Purification over silica gel provided 650 mg (4.0 mmol, 80%) of **26** as a yellow oil. R_f = 0.48 (PE/EtOAc 2:1). ^{1}H NMR (300 MHz) δ = 5.22 (s, 1H), 4.29 (m, 2H), 2.10 (m, 2H), 1.79 (m, 1H), 1.69 (m, 2H), 1.02 (t, *J* = 7.4 Hz, 3H) ppm. ^{13}C NMR (75 MHz) δ = 161.0, 88.3, 80.1, 69.3, 37.8, 31.1, 8.7 ppm. MS (CI): *m*/*z* = 162.0. IR (neat): ν 3387, 2968, 2934, 2905, 1674. HRMS (CI): Calcd for $C_7H_{12}ClO_2$; 163.0526. Found: 163.0527.

(*Z*)-2-(Chloromethylene)-3-isopropyltetrahydrofuran-3-ol (27)

To a solution of 1.0 mL (12.5 mmol, 2.5 equiv) of 1,1-dichloroethylene in 30 mL of THF, cooled to 0 °C, was added 2.81 g (25.0 mmol, 5 equiv) of *t*-BuOK. After 15 minutes, 580 mg (5.0 mmol, 1 equiv) of hydroxyketone **19** in 5 mL of THF was added to the reaction. After one hour, 20 mL of water was poured in the reaction and the pH was brought back to 7 by the addition of diluted sulfuric acid. The aqueous layer was extracted with 2 × 30 mL of dichloromethane. The organic phase was dried over magnesium sulfate, filtered and concentrated. Purification over silica gel provided 645 mg (3.7 mmol, 73%) of **27** as a yellow oil. R_f = 0.56 (PE/EtOAc 2:1). ^{1}H NMR (300 MHz) δ = 5.21 (s, 1H), 4.29 (m, 2H), 2.18 (m, 1H), 1.95 (m, 2H), 1.64 (s, 1H), 1.07 (d, *J* = 6.8 Hz, 3H), 0.96 (d, *J* = 6.8 Hz, 3H) ppm. ^{13}C NMR (75 MHz) δ = 160.6, 88.7, 83.3, 69.5, 34.8, 34.0, 18.5, 17.3 ppm. MS (CI): *m*/*z* = 176.1. IR (neat): ν 3381, 2980, 2962, 2951, 2939, 2918, 2876, 1668. HRMS (CI): Calcd for $C_8H_{14}ClO_2$; 177.0682. Found: 177.0686.

(*Z*)-2-(Chloromethylene)-3-phenyltetrahydrofuran-3-ol (28) using the *t*-BuOK procedure

To a solution of 1.0 mL (12.5 mmol, 2.5 equiv) of 1,1-dichloroethylene in 30 mL of THF, cooled to 0 °C, was added 2.81 g (25.0 mmol, 5 equiv) of *t*-BuOK. After 15 minutes, 750 mg (5.0 mmol, 1 equiv) of hydroxyketone **20** in 5 mL of THF was added to the reaction. After one hour, 20 mL of water was poured into the reaction and the pH was brought back to 7 by the addition of diluted sulfuric acid. The aqueous layer was extracted with 2 × 30 mL of dichloromethane. The organic phase was dried over magnesium sulfate, filtered and concentrated. Purification over silica gel provided 400 mg (1.9 mmol, 38%) of **28** as an orange oil. R_f = 0.63 (PE/EtOAc 2:1). ^{1}H NMR (300 MHz) δ = 7.52 (d, *J* = 7.2 Hz, 2H), 7.33 (m, 3H), 4.94 (s, 1H), 4.47 (m, 2H), 2.42 (m, 2H), 2.13 (s, 1H) ppm. ^{13}C NMR (75 MHz) δ = 162.6, 141.3, 128.4, 128.1, 125.9, 91.3, 81.6, 70.0, 43.5 ppm. MS (CI): *m*/*z* = 210.1. IR (neat): ν 3425, 3105, 3011, 2986, 2959, 2916, 1674. HRMS (CI): Calcd for $C_{11}H_{12}ClO_2$; 211.0526. Found: 211.0521.

(*Z*)-2-(Chloromethylene)-3-phenyltetrahydrofuran-3-ol (28) using LDA procedure

To a solution of 1.7 mL (12.3 mmol, 4.4 equiv) of diisopropylamine in 15 mL of THF, cooled to 0 °C, was added 4.9 mL (12.3 mmol, 4.4 equiv, 2.5 M in hexane) of *n*-BuLi. The mixture was cooled to −78 °C before the addition of 0.50 mL (6.2 mmol, 2.2 equiv) of 1,1-dichloroethylene. The mixture was brought back to 0 °C and 420 mg (2.8 mmol, 1 equiv) of hydroxyketone **20** was added. After two hours, 15 mL of aqueous NH_4Cl were poured in the reaction. The aqueous layer was extracted with 2 × 20 mL of diethyl ether. The organic phase was dried over magnesium sulfate, filtered and concentrated. The crude mixture was dissolved in 15 mL of THF and cooled to 0 °C before the addition of 314 mg (2.8 mmol, 1 equiv) of *t*-BuOK. After one hour, 10 mL of water was poured into the reaction and the pH was brought back to 7 by the addition of diluted sulfuric acid. The aqueous layer was extracted with 2 × 15 mL of dichloromethane. The organic phase was dried over magnesium sulfate, filtered and concentrated. Purification over silica gel provided 277 mg (1.3 mmol, 47%) of **28** as a yellow oil.

(*Z*)-2-(Chloromethylene)-3-isopropyl-5,5-dimethyltetrahydrofuran-3-ol (29)

To a solution of 8.0 mL (57.1 mmol, 4.4 equiv) of diisopropylamine in 50 mL of THF, cooled to 0 °C, was added 23.0 mL (57.1 mmol, 4.4 equiv, 2.5 M in hexane) of *n*-BuLi. The mixture was cooled to −78 °C before the addition of 2.3 mL (28.5 mmol, 2.2 equiv) of 1,1-dichloroethylene. The mixture was brought back to 0 °C and 1.87 g (13.0 mmol, 1 equiv) of hydroxyketone **21** in 8 mL of THF was added. After one hour, 50 mL of aqueous NH_4Cl was poured into the reaction. The aqueous layer was extracted with 3 × 30 mL of diethyl ether. The organic phase was dried over magnesium sulfate, filtered and concentrated. The crude mixture was dissolved in 60 mL of THF and cooled to 0 °C before the addition of 1.46 g (13.0 mmol, 1 equiv) of *t*-BuOK. After one hour, 20 mL of water was poured into the reaction and the pH was brought back to 7 by the addition of diluted sulfuric acid. The aqueous layer was extracted with 3 × 30 mL of dichloromethane. The organic phase was dried over magnesium sulfate, filtered and concentrated. Purification over silica gel provided 1.06 g (5.2 mmol, 40%) of **29** as a white solid. R_f = 0.69 (PE/EtOAc 2:1). ^{1}H NMR (300 MHz) δ = 5.15 (s, 1H), 2.00 (d, *J* = 13.5 Hz, 1H), 1.87 (d, *J* = 13.8 Hz, 1H), 1.87 (m, 1H), 1,66 (s, 1H), 1.50 (s, 3H), 1.43 (s, 3H), 1.01 (d, *J* = 6.6 Hz, 3H), 0.92 (d, *J* = 6.6 Hz, 3H) ppm. ^{13}C NMR (75 MHz) δ = 161.4, 88.2, 85.6, 85.3, 45.0, 36.4, 29.1, 28.7, 18.7, 17.2 ppm. MS (CI): *m*/*z* = 204.1. IR (neat): ν 3501, 2976, 2935, 2874, 1666. HRMS (CI): Calcd for $C_{10}H_{18}ClO_2$; 205.0995. Found: 205.0991. mp 59°C.

(*Z*)-3-(Chloromethylene)-4-methyl-2-oxaspiro[4.5]decan-4-ol (30)

To a solution of 0.32 mL (4.0 mmol, 2.5 equiv) of 1,1-dichloroethylene in 9 mL of THF, cooled to 0 °C, was added 900 mg (8.0 mmol, 5 equiv) of *t*-BuOK. After 15 minutes, 250 mg (1.6 mmol, 1 equiv) of hydroxyketone **22** in 2 mL of THF was added to the reaction. After one hour, 5 mL of water was poured into the reaction and the pH was brought back to 7 by the addition of diluted sulfuric acid. The aqueous layer was extracted with 30 mL of dichloromethane. The organic phase was dried over magnesium sulfate, filtered and concentrated. Purification over silica gel provided 145 mg (0.7 mmol, 42%) of **30** as a yellow oil. R_f = 0.66 (PE/EtOAc 2:1). ^{1}H NMR (300 MHz) δ = 5.25 (s, 1H), 4.18 (s, 2H), 1.71 (m, 4H), 1.53 (s, 1H), 1.31 to 1.45 (m, 2H), 1.29 (s, 3H), 1.10 to 1.26 (m, 4H) ppm. ^{13}C NMR (75 MHz) δ = 162.0, 88.2, 80.2, 75.7, 48.3, 29.0, 27.1, 26.0, 23.1, 23.0, 20.4 ppm. MS (CI): *m*/*z* = 216.1. IR (neat): ν 3421, 2918, 2854, 1672. HRMS (CI): Calcd for $C_{11}H_{18}ClO_2$; 217.0995. Found: 217.0988.

(*Z*)-2-(Chloromethylene)-3,4,4-trimethyltetrahydrofuran-3-ol (31)

To a solution of 1.20 mL (15.0 mmol, 2.5 equiv) of 1,1-dichloroethylene in 40 mL of THF, cooled to 0 °C, was added 3.37 g (30.0 mmol, 5 equiv) of *t*-BuOK. After 15 minutes, 700 mg (6.0 mmol, 1 equiv) of hydroxyketone **23** in 2 mL of THF was added to the reaction. After one hour, 20 mL of water was poured in the reaction and the pH was brought back to 7 by the addition of diluted sulfuric acid. The aqueous layer was extracted with 30 mL of dichloromethane. The organic phase was dried over magnesium sulfate, filtered and concentrated. Purification over silica gel provided 820 mg (4.6 mmol, 77%) of **31** as a white solid. R_f = 0.62 (PE/EtOAc 2:1). ^{1}H NMR (300 MHz) δ = 5.27 (s, 1H), 4.11 (d, *J* = 8.0 Hz, 1H), 3.88 (d, *J* = 8.1 Hz, 1H), 1.58 (s, 1H), 1.29 (s, 3H), 1.06 (s, 3H), 0.96 (s, 3H) ppm. ^{13}C NMR (75 MHz) δ = 161.9, 88.5, 80.6, 79.7, 44.8, 22.1, 19.9, 17.7 ppm. MS (CI): *m*/*z* = 176.1. IR (neat): ν 3460, 2966, 2867, 1666. HRMS (CI): Calcd for $C_8H_{14}ClO_2$; 177.0682. Found: 177.0683. mp 64 °C.

(*Z*)-2-(Chloromethylene)-3-methyl-4-methylenetetrahydrofuran-3-ol (32)

To a solution of 5.3 mL (37.4 mmol, 4.4 equiv) of diisopropylamine in 35 mL of THF, cooled to 0 °C, was added 15.0 mL (37.4 mmol, 4.4 equiv, 2.5 M in hexane) of *n*-BuLi. The mixture was cooled to −78 °C before the addition of 1.5 mL (18.7 mmol, 2.2 equiv) of 1,1-dichloroethylene. The mixture was brought back to 0 °C and 850 mg (13.0 mmol, 1 equiv) of hydroxyketone **24** in 7 mL of THF was added. After one hour, 40 mL of aqueous NH_4Cl was poured in the reaction. The aqueous layer was extracted with 2 × 30 mL of diethyl ether. The organic phase was dried over magnesium sulfate, filtered and concentrated. The crude mixture was dissolved in 40 mL of THF and cooled to −24 °C before the addition of 950 mg (8.5 mmol, 1 equiv) of *t*-BuOK. After one hour, 20 mL of water were poured in the reaction and the pH was brought back to 7 by the addition of diluted sulfuric acid. The aqueous layer was extracted with 2 × 30 mL of dichloromethane. The organic phase was dried over magnesium sulfate, filtered and concentrated. Purification over silica gel provided 835 mg (5.2 mmol, 61%) of **32** as a white solid. R_f = 0.52 (PE/EtOAc 2:1). ^{1}H NMR (300 MHz) δ = 5.37 (s, 1H), 5.35 (t, *J* = 2.1 Hz, 1H), 5.13 (t, *J* = 1.6 Hz, 1H), 4.80 (m, 2H), 2,41 (s, 1H), 1.50 (s, 3H) ppm. ^{13}C NMR (75 MHz) δ = 161.5, 150.2, 107.1, 89.2, 76.2, 72.0, 27.4 ppm. MS (EI): *m*/*z* = 160.0. IR (neat): ν 3383, 3117, 2976, 2934, 2881, 1684, 1661. HRMS (EI): Calcd for $C_7H_9ClO_2$; 160.0286. Found: 160.0288. mp 44 °C.

2-(Chloromethyl)-3-methyltetrahydrofuran-2,3-diol (34)

To a solution of 450 mg (3.0 mmol, 1 equiv) of chloromethylenefuran **13** in 15 mL of dichloromethane was added 1.3 mL (15.0 mmol, 5 equiv, 36% in water) of HCl. After 2 minutes, the pH was brought back to 7 with aqueous Na_2CO_3 before the addition of 10 mL of water. The aqueous layer was extracted with 2 × 15 mL of dichloromethane. The organic phase was dried over magnesium sulfate, filtered and concentrated. Purification over silica gel provided 260 mg (1.6 mmol, 52%) of **34** (1:1 mixture) as a yellow oil. R_f = 0.24 (PE/EtOAc 2:1). ^{1}H NMR (300 MHz) δ = 4.22 (s, 1H), 4.00 (m, 3H), 3.79 (m, 3H), 3.63 (s, 2H), 3.48 (s, 1H), 3.08 (s, 1H), 2.49 (s, 1H), 2.27 (m, 2H), 1.95 (m, 2H), 1.38 (s, 3H), 1.30 (s, 3H) ppm. ^{13}C NMR (75 MHz) δ = 104.1, 101.6, 81.1, 78.9, 64.9, 64.5, 47.8, 47.5, 39.3, 39.0, 21.9, 21.4 ppm. MS (CI): *m*/*z* = 166.0. IR (neat): ν 3391, 2984, 2945, 2901. HRMS (CI): Calcd for $C_6H_{10}ClO_2$; 149.0369. Found: 149.0364.

4a,8a-Bis(chloromethyl)-3a,7a-dimethyloctahydrodifuro[2,3-*b*:2',3'-*e*][1,4]dioxine (35) and (36)

To a solution of 450 mg (3.0 mmol, 1 equiv) of chloromethylenefuran **13** in 15 mL of dichloromethane were added 57 mg (0.3 mmol, 0.1 equiv) of *para*-toluenesulfonic acid monohydrate. After 2 hours under reflux, the pH was brought back to 7 with aqueous Na_2CO_3 before the addition of 30 mL of water. The aqueous layer was extracted with 2 × 30 mL of dichloromethane. The organic phase was dried over magnesium sulfate, filtered and concentrated. Purification over silica gel provided 52 mg (0.18 mmol, 11%) of the *anti* isomer **35** as a white solid and 243 mg (0.82 mmol, 54%) of the *syn* isomer **36** as a white solid.

For the *anti* isomer **35**: R_f = 0.73 (PE/EtOAc 2:1). ^{1}H NMR (300 MHz) δ = 4.06 (m, 2H), 3.90 (t, *J* = 8.0 Hz, 2H), 3.52 (d, *J* = 11.8 Hz, 2H), 3.44 (d, *J* = 11.8 Hz, 2H), 2.21 (m, 2H), 2.08 (dd, *J* = 13.0 Hz, *J* = 5.4 Hz, 2H), 1.48 (s, 6 H) ppm. ^{13}C NMR (75 MHz) δ = 103.9, 77.5, 65.4, 48.1, 40.4, 20.9 ppm. MS (CI): *m*/*z* = 296.1. IR (neat): ν 2970, 2901. HRMS (CI): Calcd for $C_{12}H_{19}Cl_2O_4$; 297.0660. Found: 297.0661. mp 153 °C.

For the *syn* isomer **36**: R_f = 0.66 (PE/EtOAc 2:1). ^{1}H NMR (300 MHz) δ = 4.16 (dd, *J* = 16.1 Hz, *J* = 8.1 Hz, 2H), 3.95 (m, 2H), 3.63 (d, *J* = 12.0 Hz, 2H), 3.56 (d, *J* = 12.0 Hz, 2H), 2.64 (m, 2H), 2.12 (m, 2H), 1.44 (s, 6H) ppm. ^{13}C NMR (75 MHz) δ = 101.7, 79.8, 65.8, 47.4, 39.1, 22.5 ppm. MS (CI): *m/z* = 296.1. IR (neat): ν 2964, 2905. HRMS (CI): Calcd for $C_{12}H_{19}Cl_2O_4$; 297.0660. Found: 297.0660. mp 92 °C.

4a,8a-Bis(chloromethyl)-3a,7a-diethyloctahydrodifuro[2,3-*b*:2',3'-*e*][1,4]dioxine (37)

To a solution of 590 mg (3.6 mmol, 1 equiv) of chloromethylenefuran **26** in 18 mL of dichloromethane, cooled to 0 °C, was added 69 mg (0.4 mmol, 0.1 equiv) of *para*-toluenesulfonic acid monohydrate. After one hour, the pH was brought back to 7 with aqueous Na_2CO_3 before the addition of 25 mL of water. The aqueous layer was extracted with 2 × 20 mL of dichloromethane. The organic phase was dried over magnesium sulfate, filtered and concentrated. Purification over silica gel provided 230 mg (0.7 mmol, 39%) of **37** as a white solid. R_f = 0.65 (PE/EtOAc 2:1). ^{1}H NMR (300 MHz) δ = 4.16 (q, *J* = 8.1 Hz, 2H), 3.94 (m, 2H), 3.62 (d, *J* = 11.8 Hz, 2H), 3.56 (d, *J* = 11.8 Hz, 2H), 2.41 (m, 2H), 2.15 (m, 2H), 1.67 (m, 4H), 1.07 (t, *J* = 7.3 Hz, 6H) ppm. ^{13}C NMR (75 MHz) δ = 102.9, 82.0, 66.2, 47.9, 37.2, 29.1, 8.5 ppm. MS (CI): *m/z* = 324.1. IR (neat): ν 2964, 2943, 2901. HRMS (CI): Calcd for $C_{14}H_{23}Cl_2O_4$; 325.0973. Found: 325.0977. mp 72 °C.

4a,8a-Bis(chloromethyl)-3a,7a-diisopropyloctahydrodifuro[2,3-*b*:2',3'-*e*][1,4]dioxine (38)

To a solution of 600 mg (3.4 mmol, 1 equiv) of chloromethylenefuran **27** in 17 mL of dichloromethane, cooled to 0 °C, was added 65 mg (0.3 mmol, 0.1 equiv) of *para*-toluenesulfonic acid monohydrate. After one hour, the pH was brought back to 7 with aqueous Na_2CO_3 before the addition of 25 mL of water. The aqueous layer was extracted with 2 × 20 mL of dichloromethane. The organic phase was dried over magnesium sulfate, filtered and concentrated. Purification over silica gel provided 120 mg (0.3 mmol, 20%) of **38** as a white solid. R_f = 0.73 (PE/EtOAc 2:1). ^{1}H NMR (300 MHz) δ = 3.99 (m, 2H), 3.87 (m, 2H), 3.75 (d, *J* = 11.4 Hz, 2H), 3.69 (d, *J* = 11.7 Hz, 2H), 2.41 (m, 2H), 2.19 (m, 2H), 2.03 (hept, *J* = 6.6 Hz, 2H), 1.04 (d, *J* = 6.6 Hz, 6H), 1.00 (d, *J* = 6.6 Hz, 6H) ppm. ^{13}C NMR (75 MHz) δ = 104.6, 83.7, 65.9, 48.4, 40.4, 34.7, 19.5, 18.7 ppm. MS (CI): *m/z* = 352.1. IR (neat): ν 2980, 2899. HRMS (CI): Calcd for $C_{16}H_{27}Cl_2O_4$; 353.1286. Found: 353.1288. mp 90 °C.

4a,8a-Bis(chloromethyl)-2,2,3a,6,6,7a-hexamethyloctahydrodifuro[2,3-*b*:2',3'-*e*][1,4]dioxine (39) and (40); 3'-chloro-3a'-(chloromethyl)-3,5,5,5',5',6a'-hexamethylhexahydro-3*H*,3'*H*-spiro[furan-2,2'-furo[3,2-*b*]furan]-3-ol (41)

To a solution of 370 mg (2.1 mmol, 1 equiv) of chloromethylenefuran **6** in 10 mL of dichloromethane, cooled to 0 °C, was added 40 mg (0.2 mmol, 0.1 equiv) of *para*-toluenesulfonic acid monohydrate. After 2 hours, 80 mg (0.4 mmol, 0.2 equiv) of *para*-toluenesulfonic acid monohydrate was added. The mixture was stirred for 3 hours and the pH was brought back to 7 with aqueous Na_2CO_3 before the addition of 10 mL of water. The aqueous layer was extracted with 2 × 10 mL of dichloromethane. The organic phase was dried over magnesium sulfate, filtered and concentrated. Purification over silica gel provided 180 mg (0.5 mmol, 49%) of dioxanes **39** and **40** as a white solid (1:9 *anti*:*syn* mixture) and 63 mg (0.18 mmol, 17%) of spirocyclic adduct **41** as a colorless oil.

For *syn* dioxane **40**: R_f = 0.78 (PE/EtOAc 2:1). ^{1}H NMR (300 MHz) δ = 3.59 (d, *J* = 12.0 Hz, 2H), 3.52 (d, *J* = 11.9 Hz, 2H), 2.73 (d, *J* = 13.6 Hz, 2H), 1.92 (d, *J* = 13.6 Hz, 2H), 1.50 (s, 6H), 1.47 (s, 6H), 1.29 (s, 6H) ppm. ^{13}C NMR (75 MHz) δ = 102.9, 81.3, 79.6, 52.0, 48.1,

30.7, 30.2, 23.6 ppm. MS (CI): *m*/*z* = 352.1. IR (neat): ν 2988, 2966, 2939. HRMS (CI): Calcd for $C_{16}H_{27}Cl_2O_4$; 353.1286. Found: 353.1280. mp 92 °C.

For spirocyclic adduct **41**: R_f = 0.63 (PE/EtOAc 2:1). ^{1}H NMR (300 MHz) δ = 4.35 (s, 1H), 3.94 (d, *J* = 12.4 Hz, 1H), 3.63 (d, *J* = 12.4 Hz, 1H), 2.62 (s, 1H), 2.21 (d, *J* = 13.8 Hz, 1H), 2.02 (d, *J* = 12.9 Hz, 1H), 1.95 (d, *J* = 13.5 Hz, 1H), 1.90 (d, *J* = 14.1 Hz, 1H), 1.67 (s, 3H), 1.39 (s, 6H), 1.37 (s, 3H), 1.34 (s, 3H), 1.27 (s, 3H) ppm. ^{13}C NMR (75 MHz) δ = 112.7, 94.9, 93.0, 82.5, 79.9, 78.9, 67.1, 53.1, 51.8, 46.7, 31.8, 29.9, 29.6, 29.1, 24.7, 24.0 ppm. MS (CI): *m*/*z* = 352.1. IR (neat): ν 2976, 2933. HRMS (CI): Calcd for $C_{16}H_{27}Cl_2O_4$; 353.1286. Found: 353.1274.

4-Chloro-1-(chloromethyl)-3',8-diethyl-5',5',6,6-tetramethyl-5'*H*-2,7-dioxaspiro[bicyclo[3.2.1]octane-3,2'-furan] (52) and 3'-chloro-3a'-(chloromethyl)-3,6a'-diethyl-5,5,5',5'-tetramethylhexahydro-3*H*,3'*H*-spiro[furan-2,2'-furo[3,2-*b*]furan]-3-ol (53)

To a solution of 490 mg (2.57 mmol, 1 equiv) of chloromethylenefuran **25** in 13 mL of dichloromethane, cooled to 0 °C, was added 49 mg (0.26 mmol, 0.1 equiv) of *para*-toluenesulfonic acid monohydrate. After one hour, 98 mg (0.52 mmol, 0.2 equiv) of *para*-toluenesulfonic acid monohydrate was added and the reaction was heated under reflux. The mixture was stirred for 3 hours and the pH was brought back to 7 with aqueous Na_2CO_3 before the addition of 20 mL of water. The aqueous layer was extracted with 2 × 20 mL of dichloromethane. The organic phase was dried over magnesium sulfate, filtered and concentrated. Purification over silica gel provided 60 mg (0.17 mmol, 13%) of bridged adduct **52** as a yellow oil and 200 mg (0.52 mmol, 41%) of spirocyclic adduct **53** as an orange solid.

For bridged adduct **52**: R_f = 0.79 (PE/EtOAc 5:1). ^{1}H NMR (300 MHz) δ = 5.60 (t, *J* = 1.7 Hz, 1H), 4.32 (d, *J* = 11.9 Hz, 1H), 3.94 (d, *J* = 11.9 Hz, 1H), 3.52 (d, *J* = 11.9 Hz, 1H), 2.95 (m, 1H), 2.43 (m, 1H), 2.03 (m, 2H), 1.86 (m, 2H), 1.39 (s, 3H), 1.36 (s, 3H), 1.32 (s, 3H), 1.25 (s, 3H), 1.13 (m, 6H) ppm. ^{13}C NMR (75 MHz) δ = 139.8, 132.5, 111.7, 107.4, 88.3, 82.0, 59.7, 45.9, 44.1, 37.5, 31.1, 30.9, 28.9, 28.5, 26.6, 18.4, 17.9, 11.5 ppm. MS (CI): *m*/*z* = 362.1. IR (neat): ν 2962, 2928, 2878, 1684. HRMS (CI): Calcd for $C_{18}H_{29}Cl_2O_3$; 363.1494. Found: 363.1503.

For spirocyclic adduct **53**: R_f = 0.70 (PE/EtOAc 5:1). ^{1}H NMR (300 MHz) δ = 4.40 (s, 1H), 3.96 (d, *J* = 12.4 Hz, 1H), 3.63 (d, *J* = 12.4 Hz, 1H), 2.77 (s, 1H), 2.21 (m, 2H), 1.78 to 2.05 (m, 4H), 1.70 (m, 1H), 1.58 (m, 1H), 1.40 (s, 3H), 1.39 (s, 3H), 1.36 (s, 3H), 1.25 (s, 3H), 1.02 (m, 6H) ppm. ^{13}C NMR (75 MHz) δ = 113.9, 97.7, 93.2, 82.6, 81.0, 68.2, 50.4, 47.6, 46.2, 30.6, 30.0, 29.8, 29.7, 29.2, 28.3, 9.0, 8.3 ppm. MS (CI): *m*/*z* = 380.2. IR (neat): ν 2964, 2939, 2878. HRMS (CI): Calcd for $C_{18}H_{31}Cl_2O_4$; 381.1599. Found: 381.1588. mp 89 °C.

(*E*)-3-(Chloro(3-isopropyl-5,5-dimethylfuran-2(5*H*)-ylidene)methyl)-5-(chloromethyl)-4-isopropyl-2,2-dimethyl-2,3-dihydrofuran (54)

To a solution of 610 mg (3.0 mmol, 1 equiv) of chloromethylenefuran **29** in 15 mL of dichloromethane, was added 57 mg (0.3 mmol, 0.1 equiv) of *para*-toluenesulfonic acid monohydrate. After 2 hours under reflux, 114 mg (0.6 mmol, 0.2 equiv) of *para*-toluenesulfonic acid monohydrate was added. The mixture was stirred for 3 more hours under reflux and the pH was brought back to 7 with aqueous Na_2CO_3 before the addition of 20 mL of water. The aqueous layer was extracted with 2 × 20 mL of dichloromethane. The organic

phase was dried over magnesium sulfate, filtered and concentrated. Purification over silica gel provided 320 mg (0.9 mmol, 57%) of **54** as a yellow oil. R_f = 0.78 (PE/EtOAc 5:1). ^{1}H NMR (300 MHz) δ = 6.00 (s, 1H), 4.23 (d, *J* = 11.7 Hz, 1H), 4.07 (d, *J* = 12.0 Hz, 1H), 3.92 (s, 1H), 3.12 (hept, *J* = 6.6 Hz, 1H), 2.61 (hept, *J* = 6.9 Hz, 1H), 1.38 (s, 3H), 1.31 (s, 6H), 1.30 (s, 3H), 1.13 (d, *J* = 5.7 Hz, 3H), 1.10 (m, 6H), 1.05 (d, *J* = 6.9 Hz, 3H) ppm. ^{13}C NMR (75 MHz) δ = 154.6, 146.1, 143.1, 137.3, 119.8, 105.3, 87.1, 85.4, 53.5, 37.0, 28.6, 27.4, 27.0, 26.4, 26.3, 22.9, 22.7, 22.5, 22.1, 21.6 ppm. MS (CI): *m*/*z* = 372.2. IR (neat): ν 2961, 2930, 2871, 1678, 1628, 1616. HRMS (CI): Calcd for $C_{20}H_{31}Cl_2O_2$; 373.1701. Found: 373.1695.

4-((*E*)-Chloro(3-isopropyl-5,5-dimethylfuran-2(5*H*)-ylidene)methyl)-2-(chloromethyl)-3-isopropyl-5,5-dimethyltetrahydrofuran-2-ol (55)

To a solution of 210 mg (0.56 mmol, 1 equiv) of triene **54** in 5.6 mL of dichloromethane, was added 3 ml of THF and 0.24 mL (2.8 mmol, 5 equiv, 36% in water) of HCl. After 30 minutes, 0.48 mL (5.6 mmol, 10 equiv, 36% in water) of HCl was added. After 30 minutes, 10 mL of water was poured into the mixture before neutralization with aqueous $NaHCO_3$. The aqueous layer was extracted with 2 × 20 mL of dichloromethane. The organic phase was dried over magnesium sulfate, filtered and concentrated. Purification over silica gel provided 95 mg (0.24 mmol, 43%) of **55** as a yellow solid. 110 mg (0.29, 52%) of the starting material **54** were also recovered. R_f = 0.60 (PE/EtOAc 5:1). ^{1}H NMR (300 MHz) δ = 6.01 (d, *J* = 1.2 Hz, 1H), 3.80 (d, *J* = 11.4 Hz, 1H), 3.73 (d, *J* = 11.1 Hz, 1H), 3.62 (d, *J* = 11.1 Hz, 1H), 3.13 (heptd, *J* = 6.8 Hz, *J* = 1.0 Hz, 1H), 2.67 (d, *J* = 1.2 Hz, 1H), 2.47 (ddd, *J* = 11.4 Hz, *J* = 5.7 Hz, *J* = 1.2 Hz, 1H), 1.95 (oct, *J* = 6.9 Hz, 1H), 1.40 (s, 3H), 1.31 (s, 3H), 1.30 (s, 3H), 1.21 (s, 3H), 1.13 (d, *J* = 5.4 Hz, 3H), 1.11 (d, *J* = 5.4 Hz, 3H), 1.04 (d, *J* = 5.1 Hz, 3H), 1.01 (d, *J* = 5.4 Hz, 3H) ppm. ^{13}C NMR (75 MHz) δ = 155.7, 143.1, 137.5, 104.0, 103.6, 87.1, 84.5, 51.8, 51.8, 51.6, 31.3, 27.8, 27.4, 26.9, 26.5, 25.8, 22.9, 22.7, 20.2 ppm. MS (CI): *m*/*z* = 390.2. IR (neat): ν 3400, 2966, 2926, 2872, 1628, 1616. HRMS (CI): Calcd for $C_{20}H_{31}Cl_2O_2$; 373.1701. Found: 373.1703. mp 111 °C.

References

[1] Wasserman, H.; Hearn, M.; Cochoy, R. *J. Org. Chem.* **1980,** *45,* 2875. doi:10.1021/jo01302a023

[2] Hitchcock, S.; Perron, F.; Martin, V.; Albizati, K. *Synthesis* **1990,** 1059. doi:10.1055/s-1990-27093

[3] Einhorn, J.; Soulier, J.-L.; Bacquet, C.; Lelandais, D. *Can. J. Chem.* **1983,** *61,* 584. doi:10.1139/v83-104

[4] Ueki, T.; Doe, M.; Tanaka, R.; Morimoto, Y.; Yoshihara, K.; Kinoshita, T. *J. Heterocycl. Chem.* **2001,** *38,* 165. doi:10.1002/jhet.5570380124

[5] O'Neil, G.; Miller, M.; Carter, K. *Org. Lett.* **2010,** *12,* 5350. doi:10.1021/ol1024635

[6] Kim, K.; Song, Y.; Lee, N.; Hahn, C. *Tetrahedron Lett.* **1986,** *27,* 2875. doi:10.1016/S0040-4039(00)84667-5

[7] Schneider, C.; Hansch, M.; Weide, T. *Chem.–Eur. J.* **2005,** *11,* 3010. doi:10.1002/chem.200400951

[8] Lin, K.-W.; Tsai, C.-H.; Hsieh, I.-L.; Yan, T.-H. *Org. Lett.* **2008,** *10,* 1927. doi:10.1021/ol8004326

[9] Sulmon, P.; De Kimpe, N.; Schamp, N. *Org. Prep. Proced. Int.* **1989,** *21,* 91. doi:10.1080/00304948909356351

[10] Trost, B.; Yang, H.; Thiel, O.; Frontier, A.; Brindle, C. *J. Am. Chem. Soc.* **2007,** *129,* 2206. doi:10.1021/ja067305j

[11] Gerwick, B. C., III; Fields, S. C.; Graupner, P. R.; Schmitzer, P. R.; Brewster, W. K. Methylidene Mevalonates and their Use as Herbicides. US Patent *CA2527439A1*, May 19, 2006.

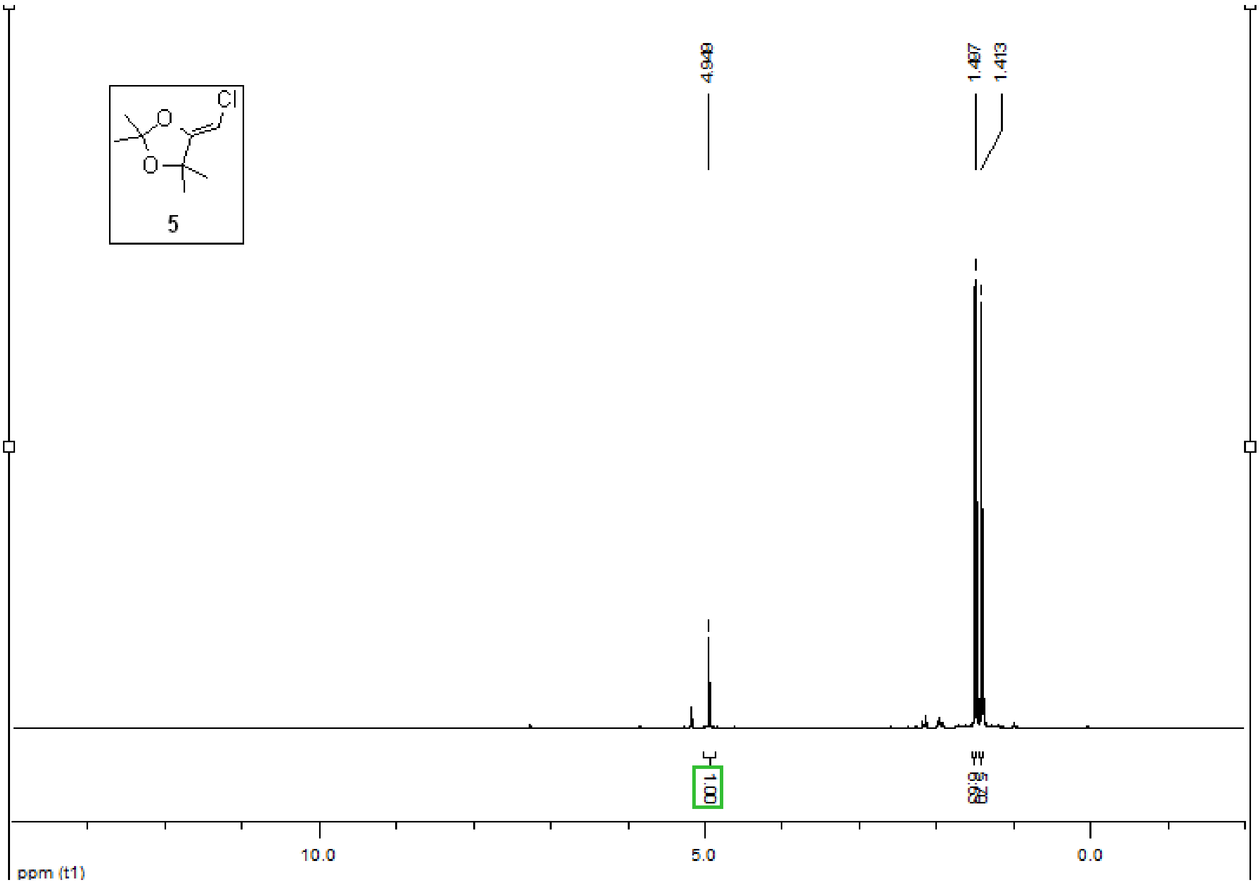
Cl
O
O
5
4.949
1.487
1.413
1.00
10.0
5.0
0.0
ppm (t1)

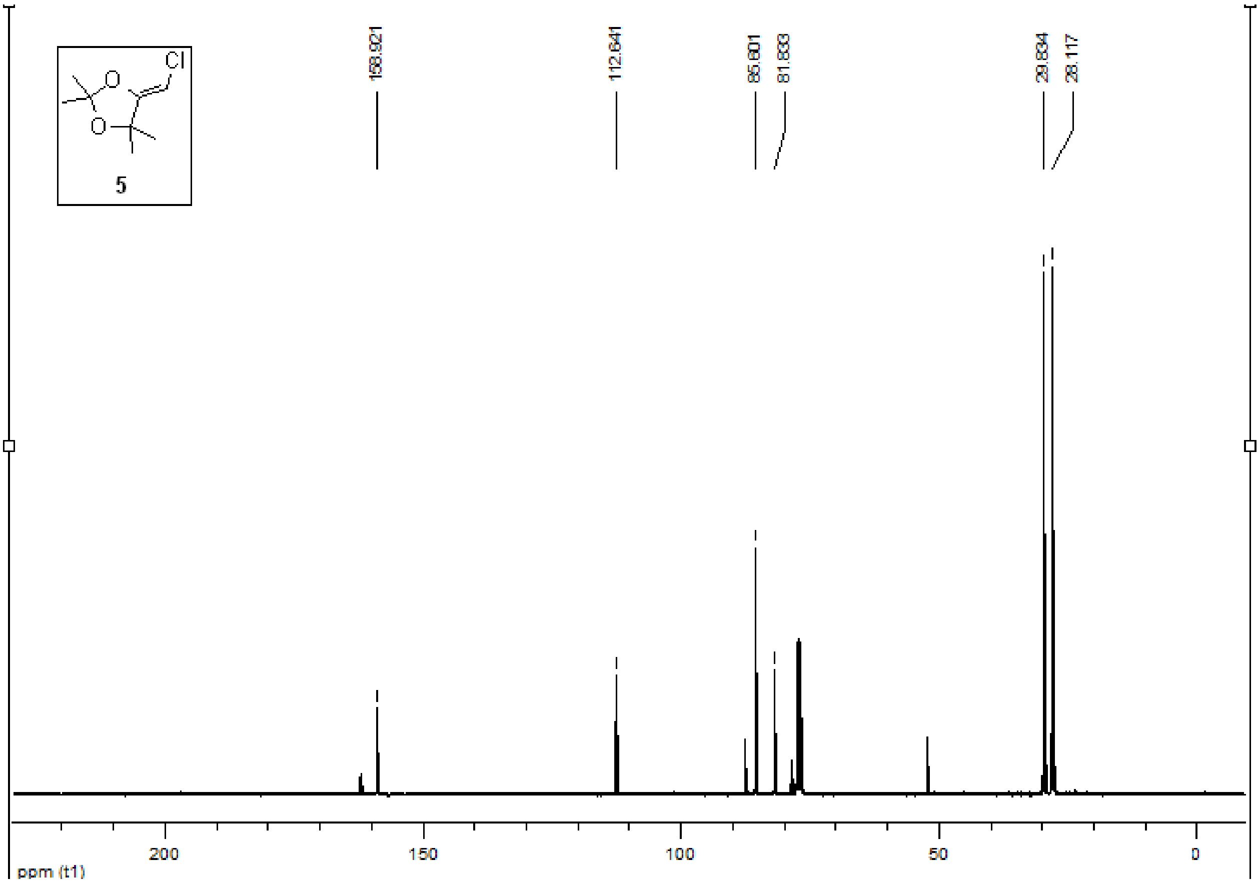
Cl
O
O
5
158.921
112.641
85.801
81.833
29.834
28.117
200
150
100
50
0
ppm (t1)

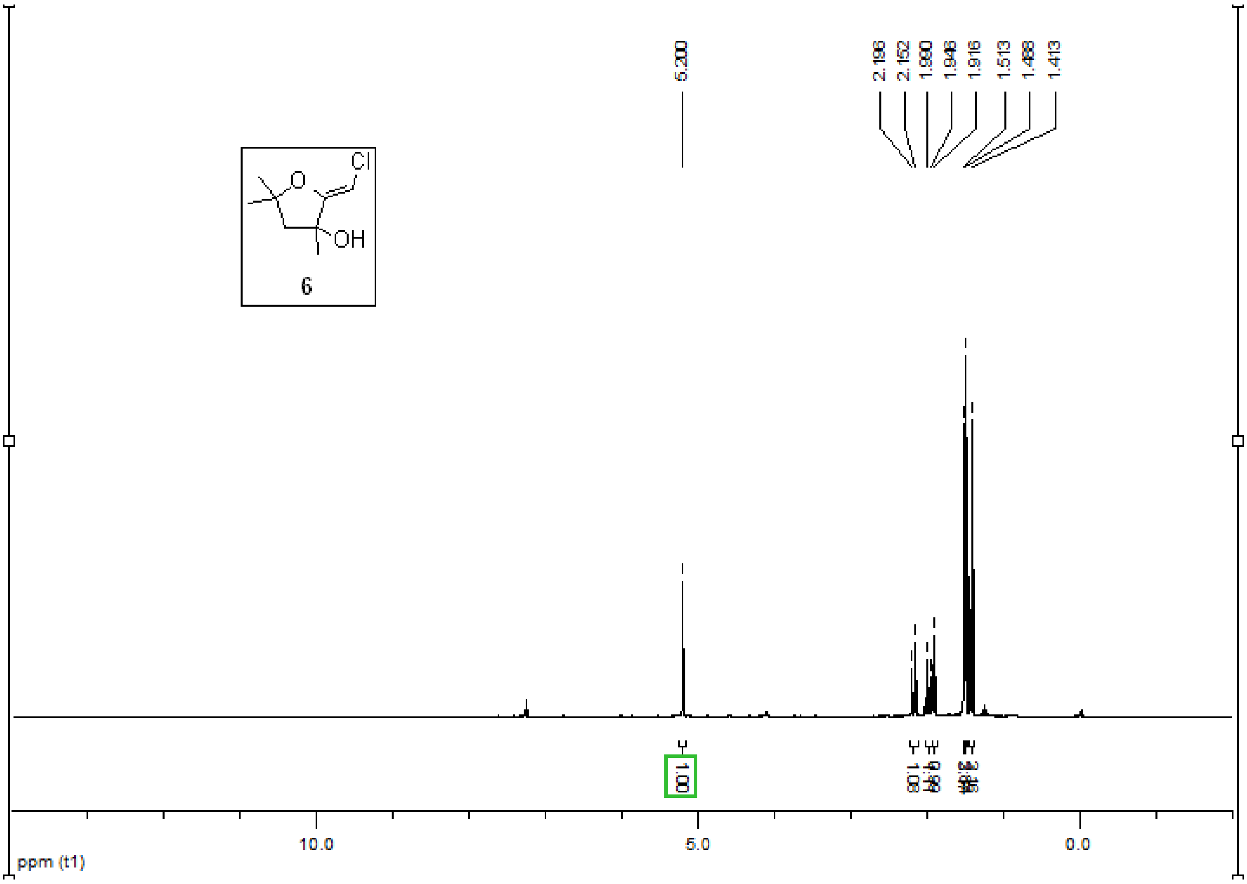
5.200
2.196
2.152
1.990
1.946
1.916
1.513
1.488
1.413
Cl
O
OH
6
1.00
1.08
10.0
5.0
0.0
ppm (t1)

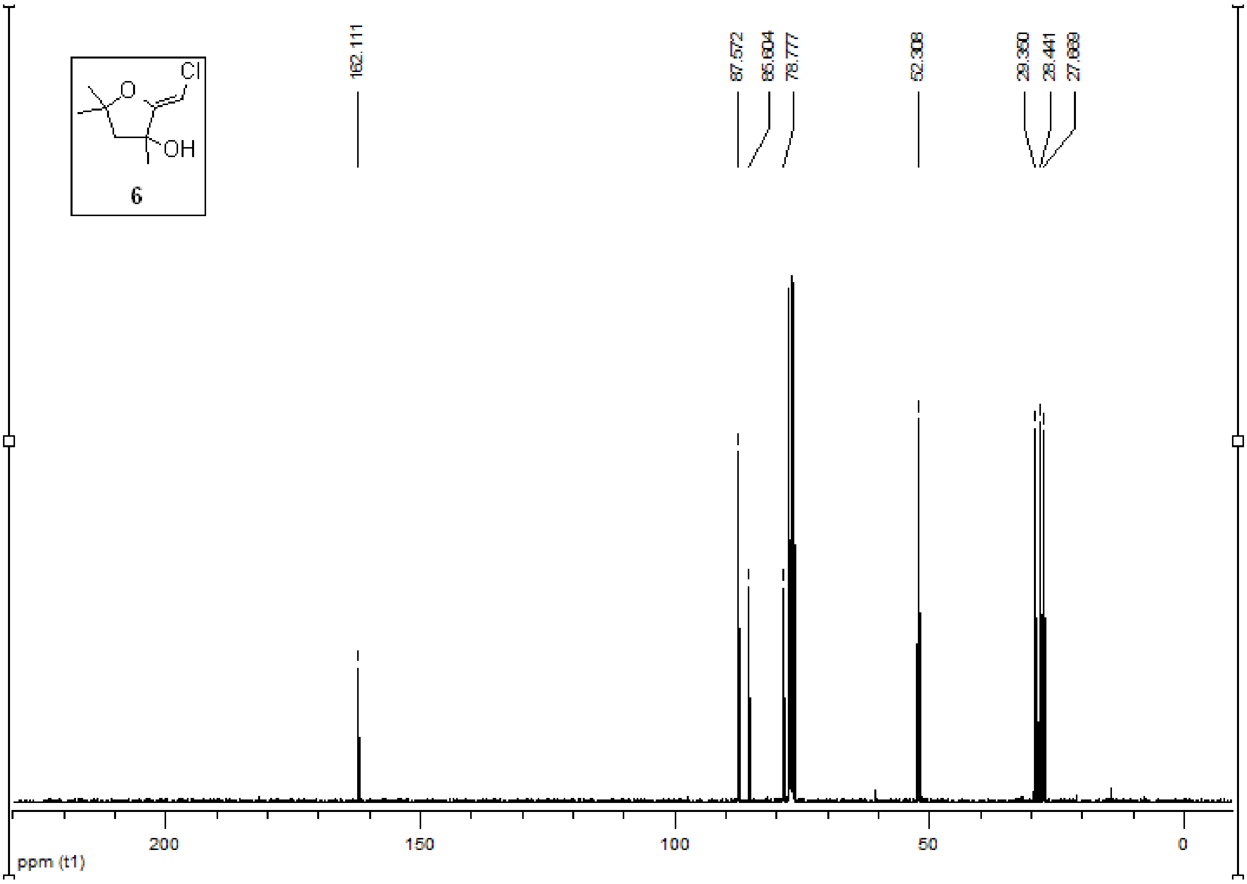
162.111
87.572
85.604
78.777
52.308
29.350
28.441
Cl
O
OH
6
200
150
100
50
0
ppm (t1)

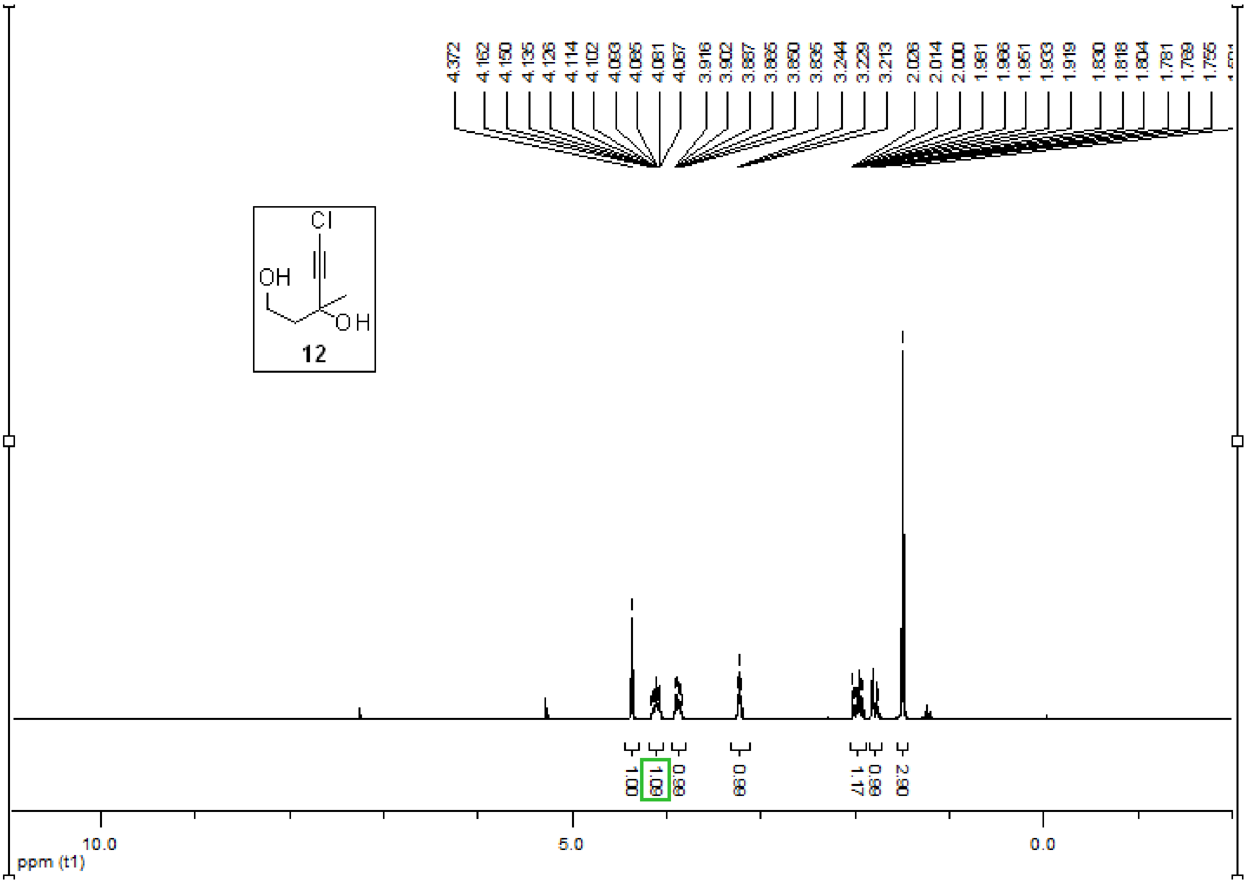

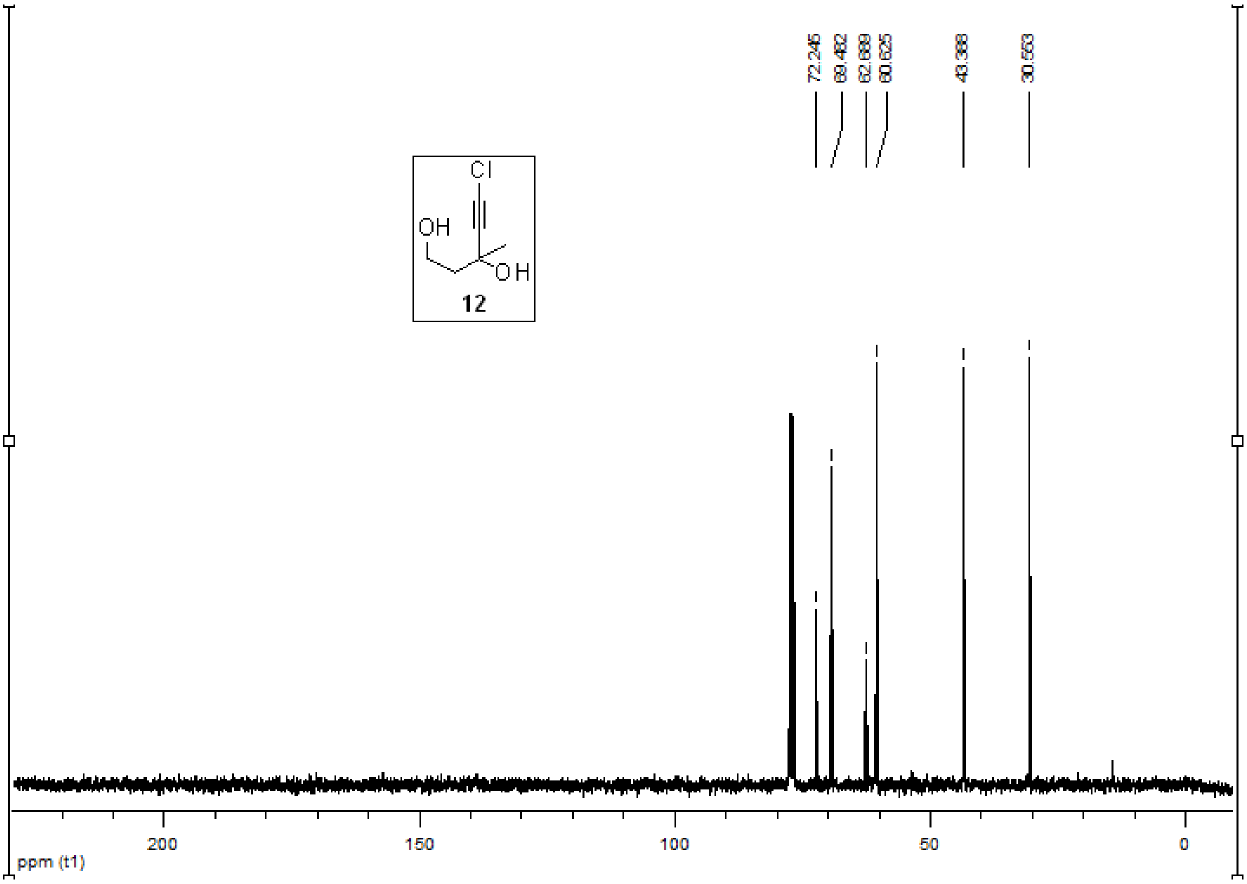

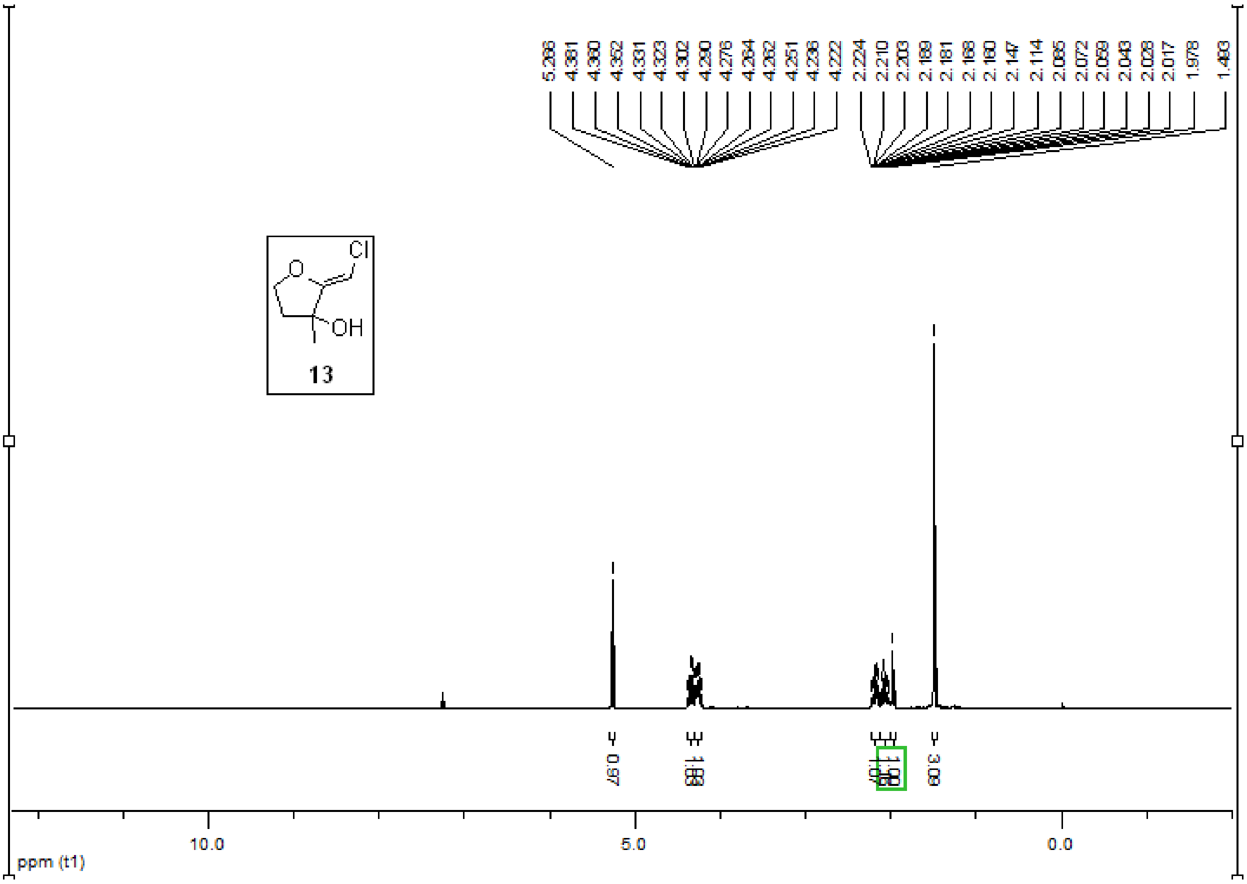

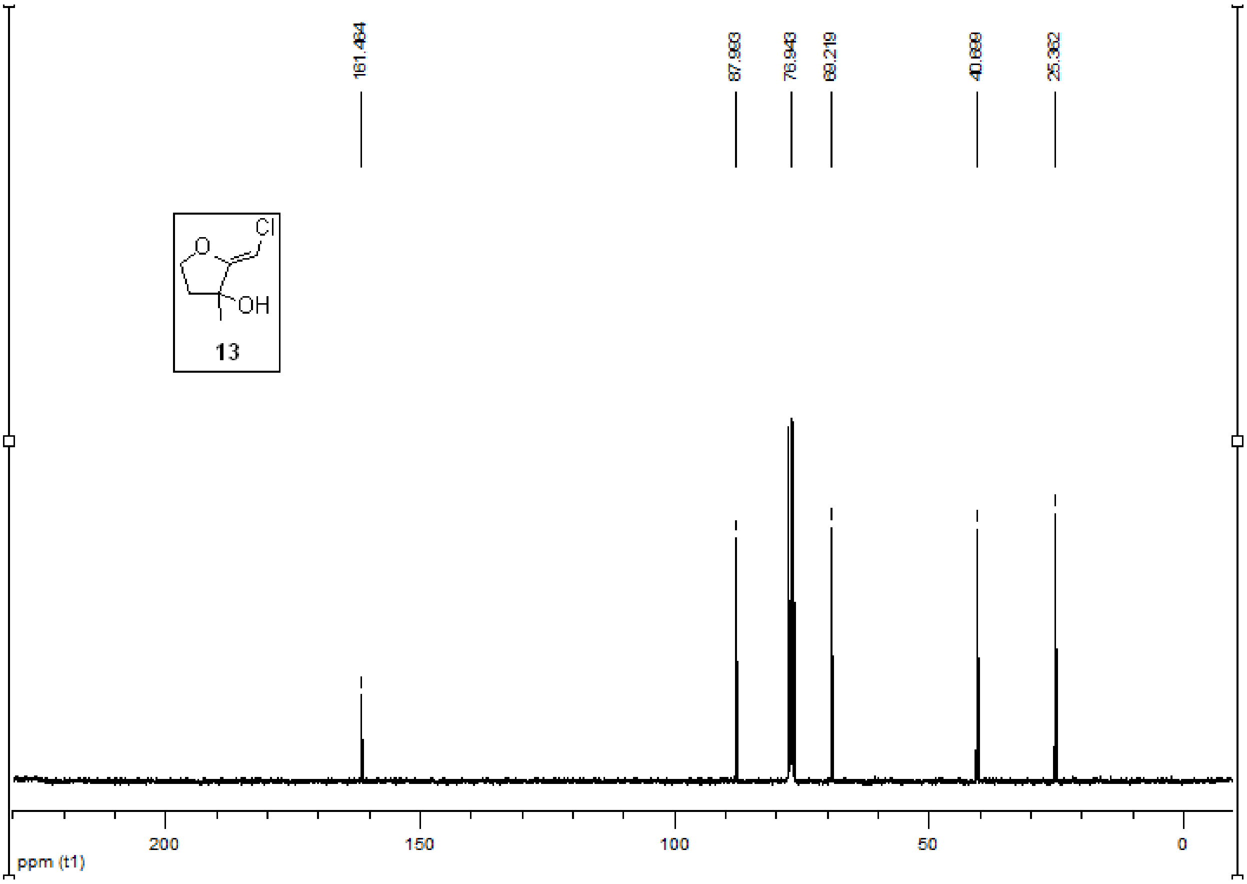

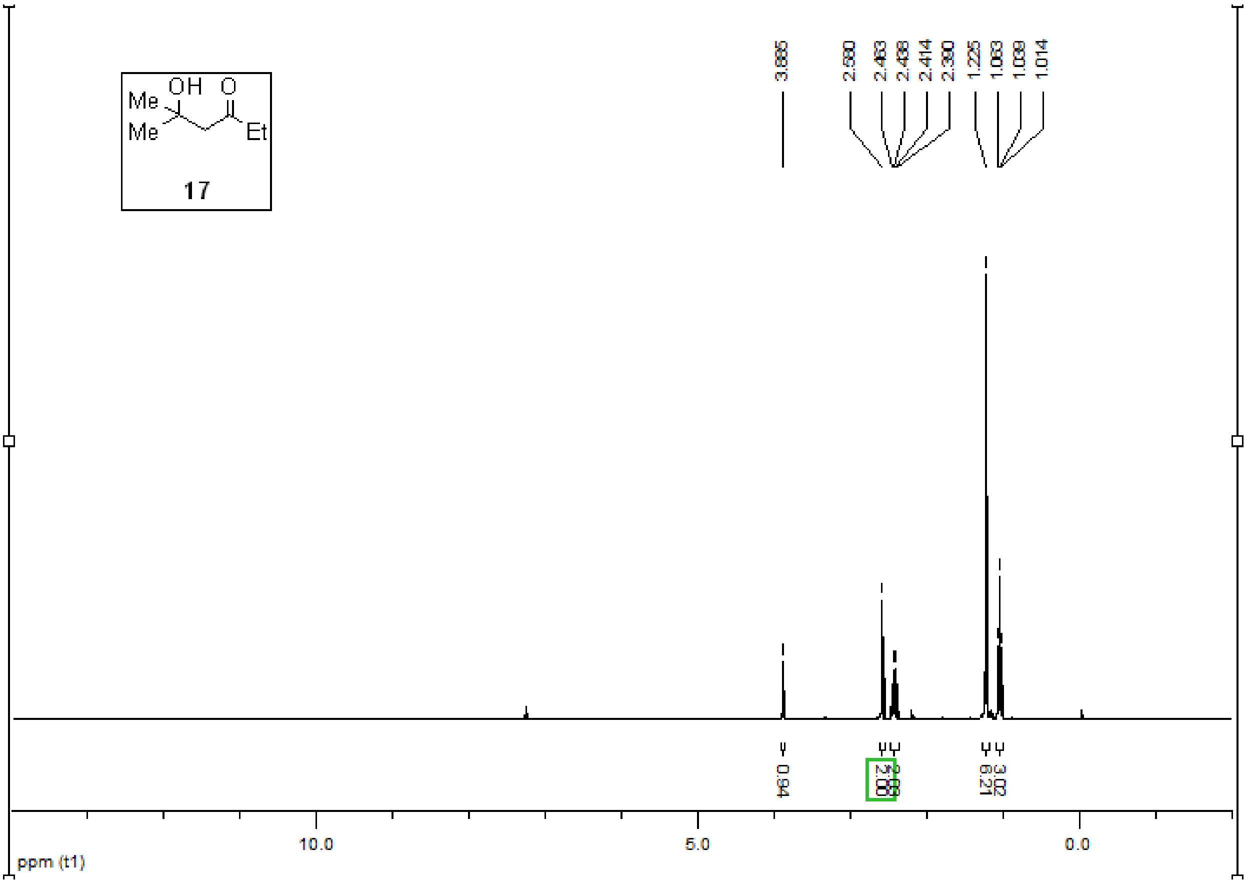
Me
OH
O
Me
Et
17
3.885
2.580
2.463
2.438
2.414
2.390
1.225
1.063
1.039
1.014
0.94
6.21
3.02
10.0
5.0
0.0
ppm (t1)

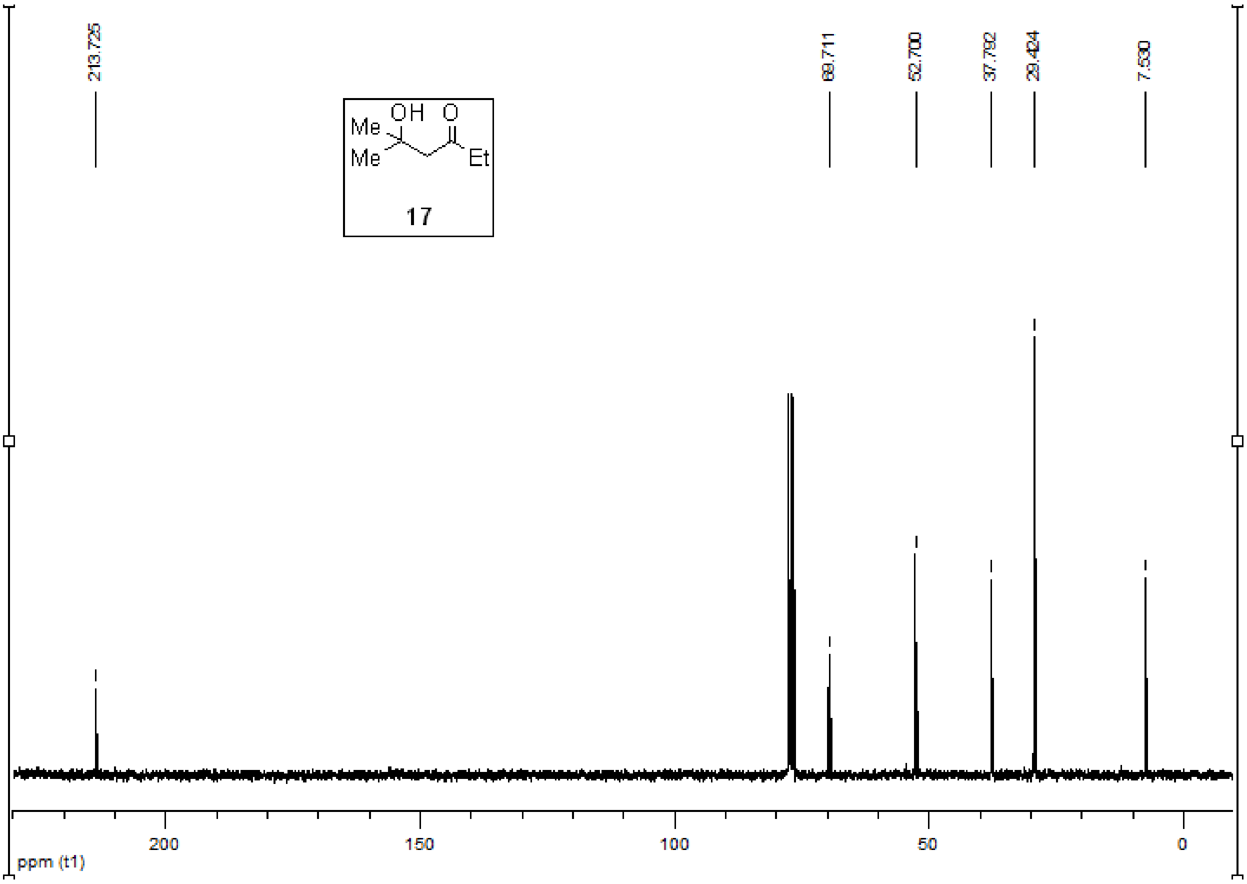
213.725
69.711
52.700
37.792
29.424
7.530
Me
OH
O
Me
Et
17
200
150
100
50
0
ppm (t1)

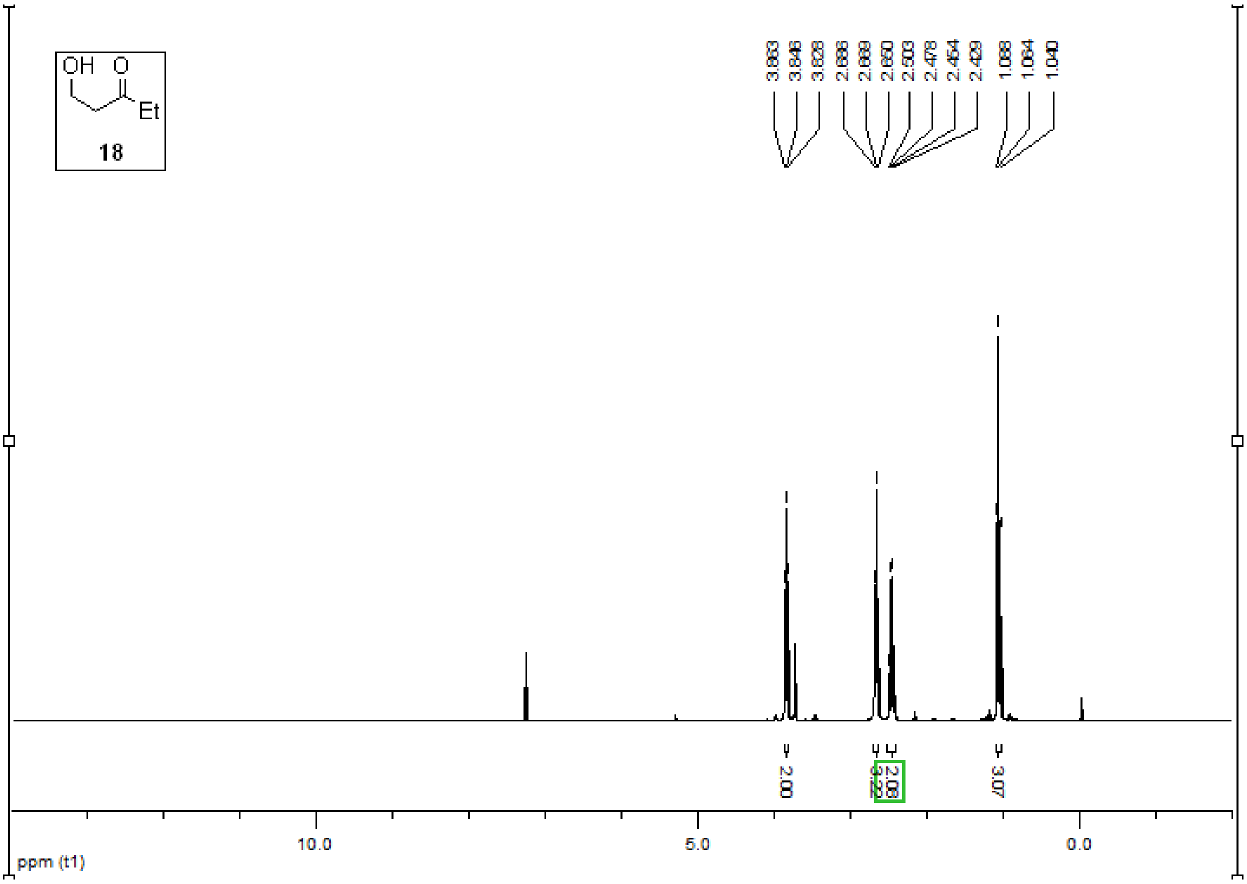
OH O
Et
18
3.863
3.846
3.828
2.686
2.669
2.650
2.503
2.478
2.454
2.429
1.088
1.064
1.040
2.00
2.08
3.07
10.0
5.0
0.0
ppm (t1)

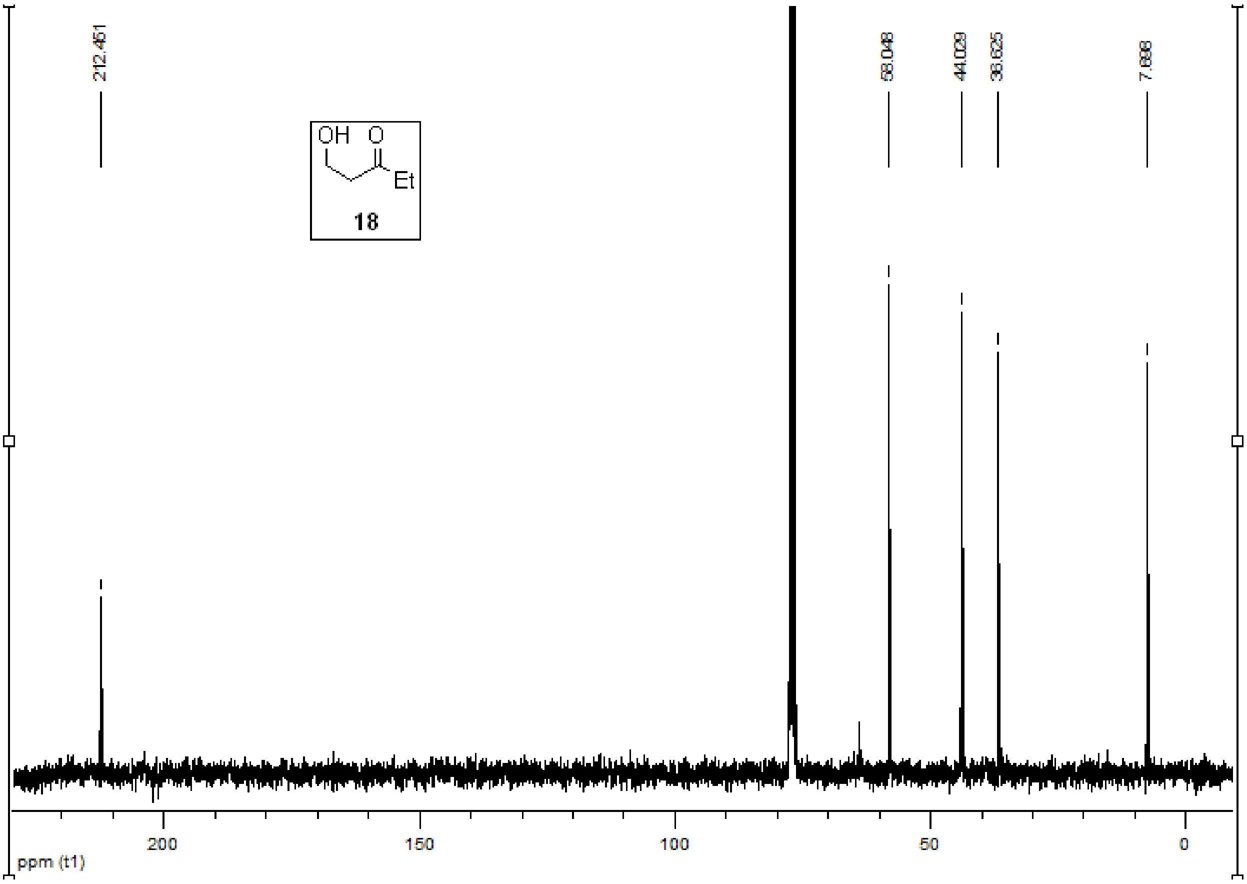
212.451
58.048
44.029
36.625
7.698
OH O
Et
18
200
150
100
50
0
ppm (t1)

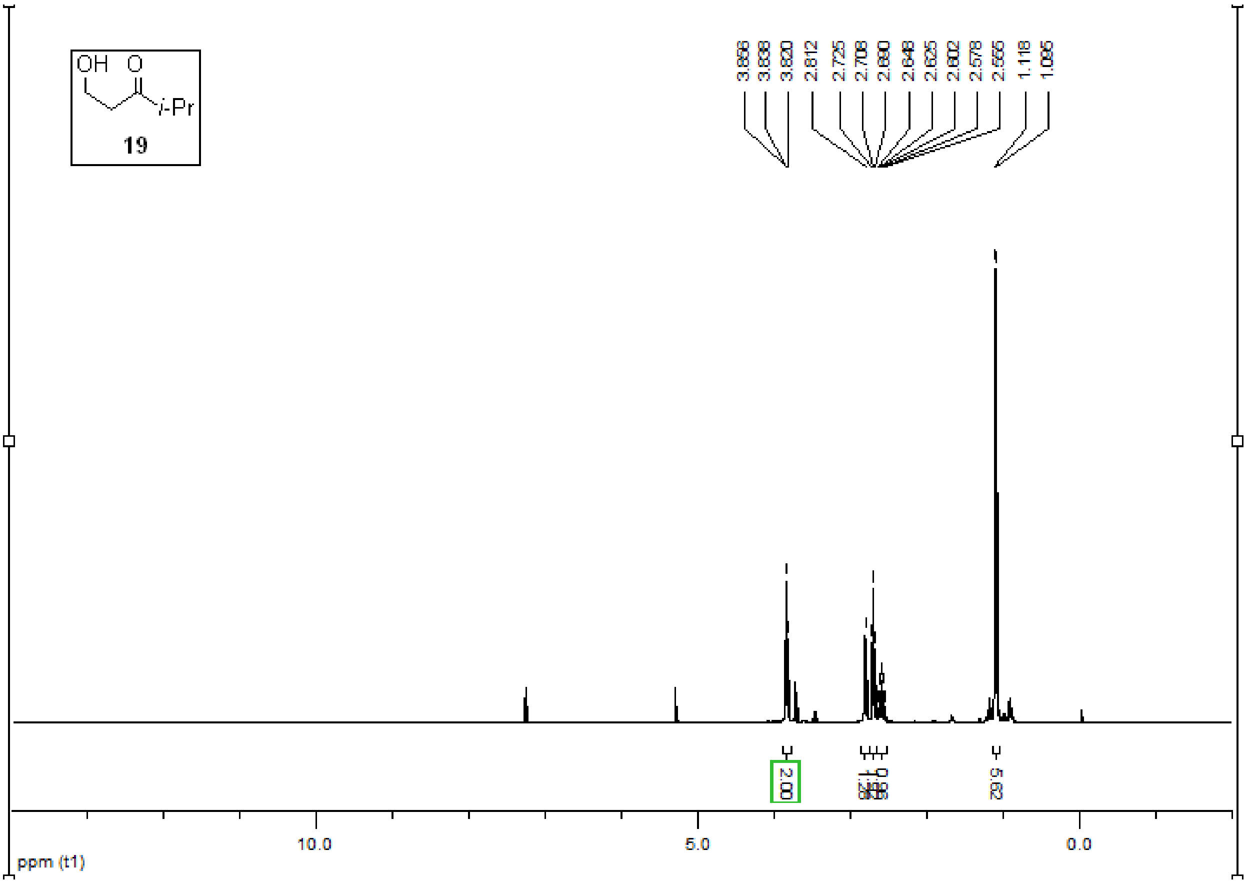
OH O
i-Pr
19
3.856
3.838
3.820
2.812
2.725
2.708
2.690
2.648
2.625
2.602
2.578
2.555
1.118
1.095
2.00
5.92
10.0
5.0
0.0
ppm (t1)

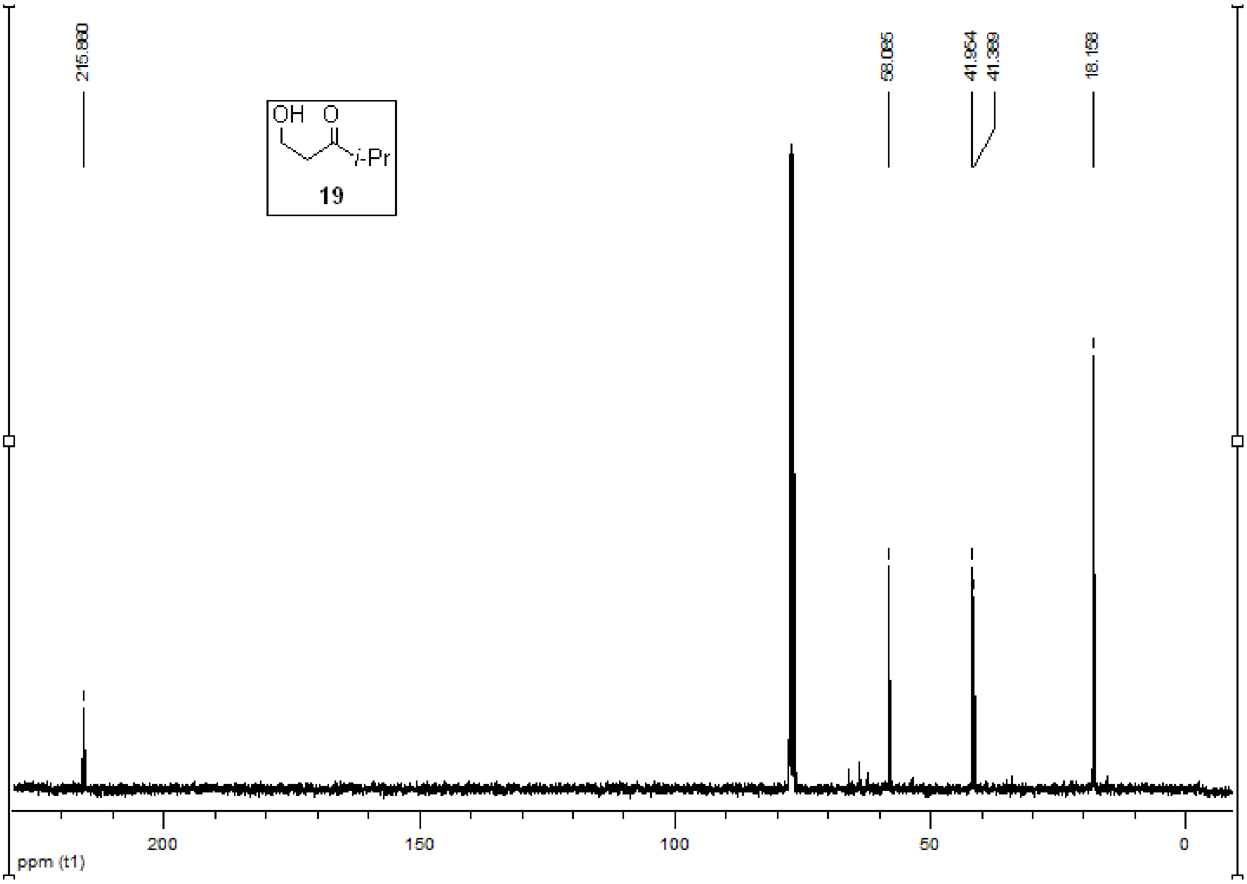
215.980
58.065
41.954
41.389
18.158
OH O
i-Pr
19
200
150
100
50
0
ppm (t1)

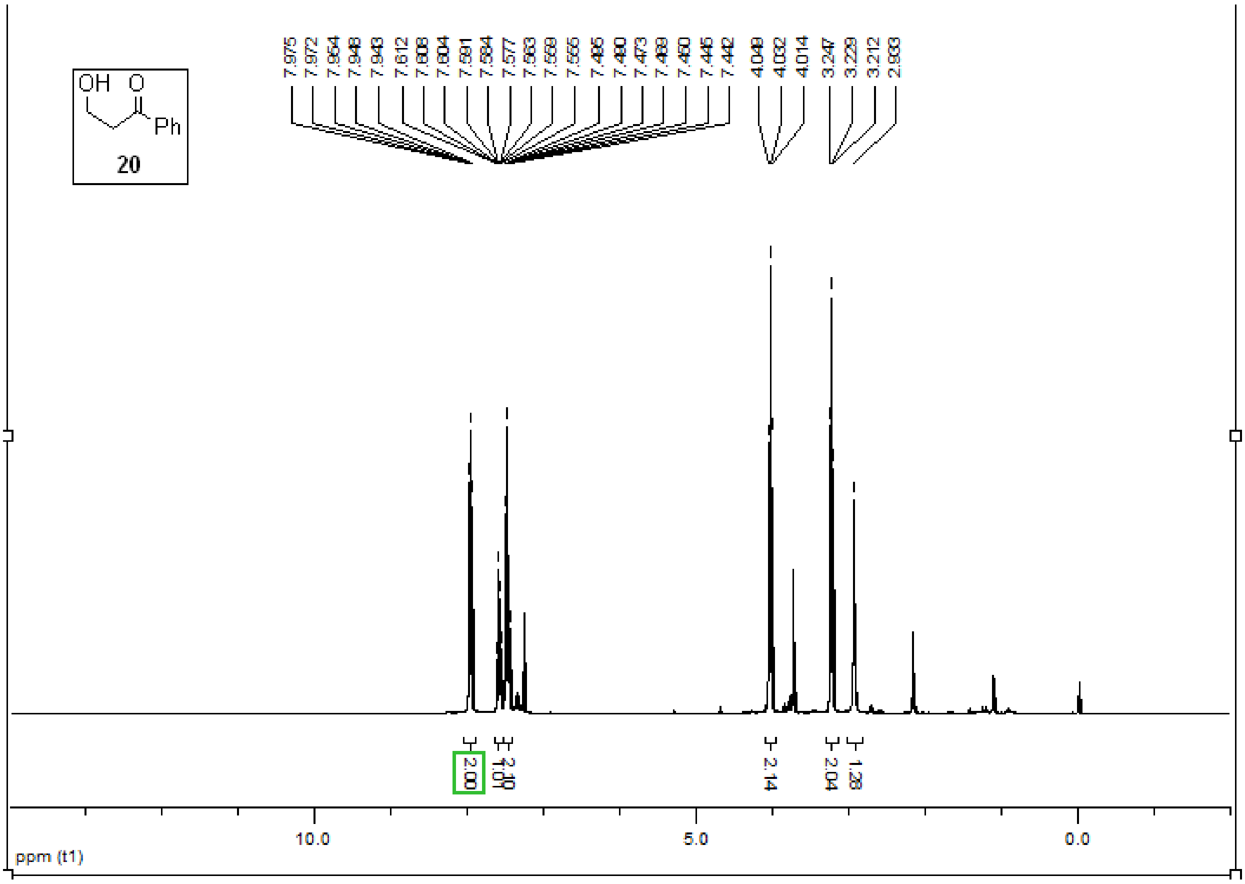

OH O
Ph
20
7.975
7.972
7.954
7.948
7.943
7.612
7.608
7.604
7.591
7.584
7.577
7.563
7.559
7.555
7.495
7.490
7.473
7.469
7.460
7.445
7.442
4.048
4.032
4.014
3.247
3.229
3.212
2.933
2.00
1.07
2.10
2.14
2.04
1.28
10.0
5.0
0.0
ppm (t1)

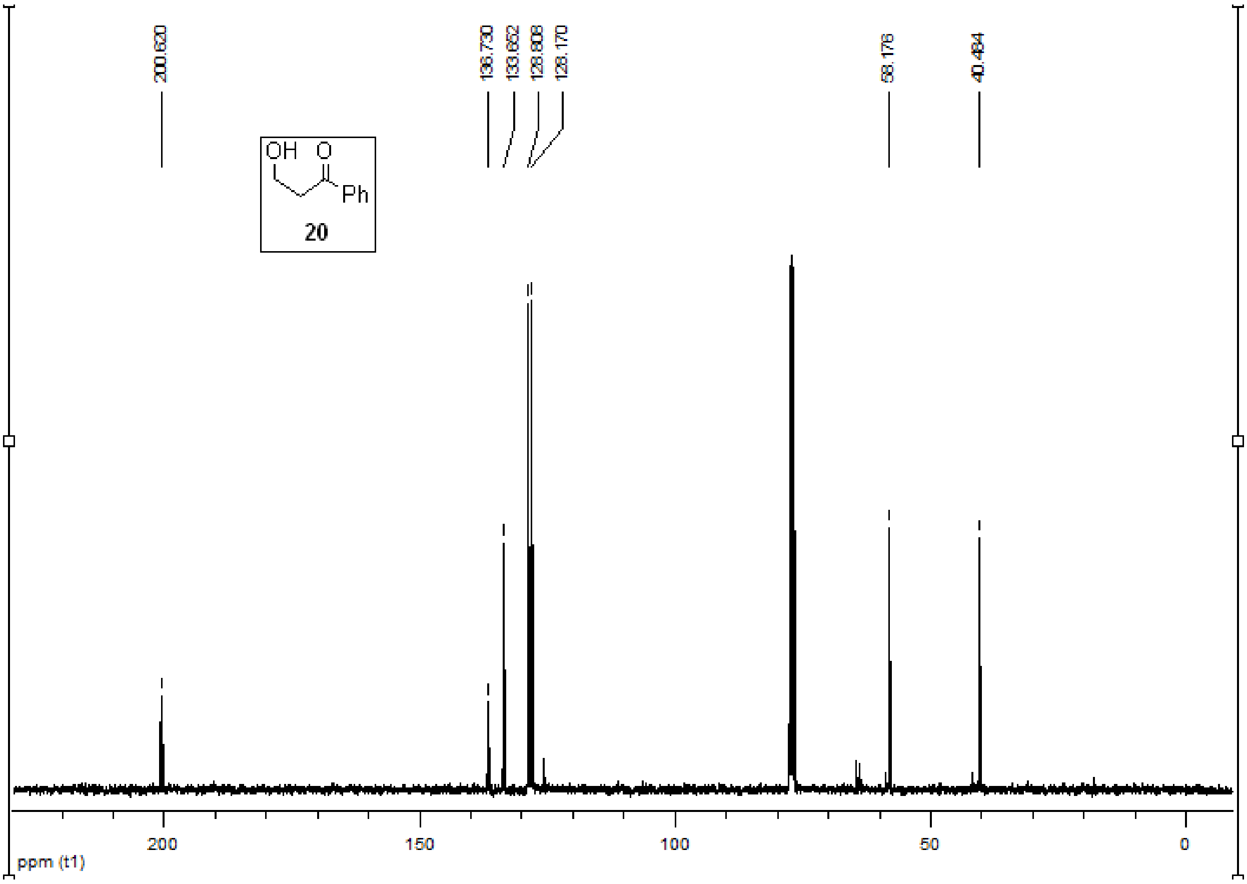

200.620
136.730
133.652
128.808
128.170
58.176
40.494
OH O
Ph
20
200
150
100
50
0
ppm (t1)

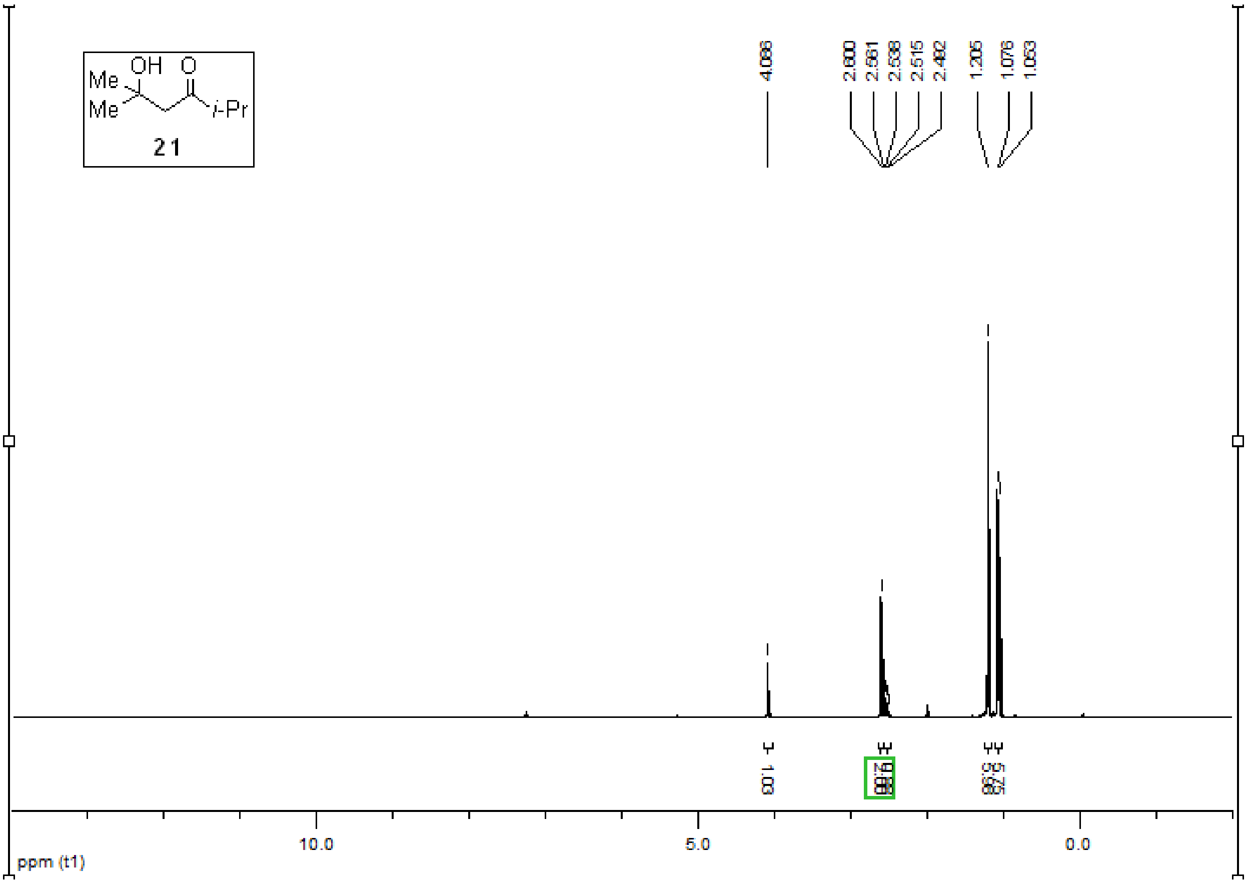
Me
OH
O
Me
i-Pr
21
4.086
2.600
2.561
2.538
2.515
2.492
1.205
1.076
1.053
1.03
10.0
5.0
0.0
ppm (t1)

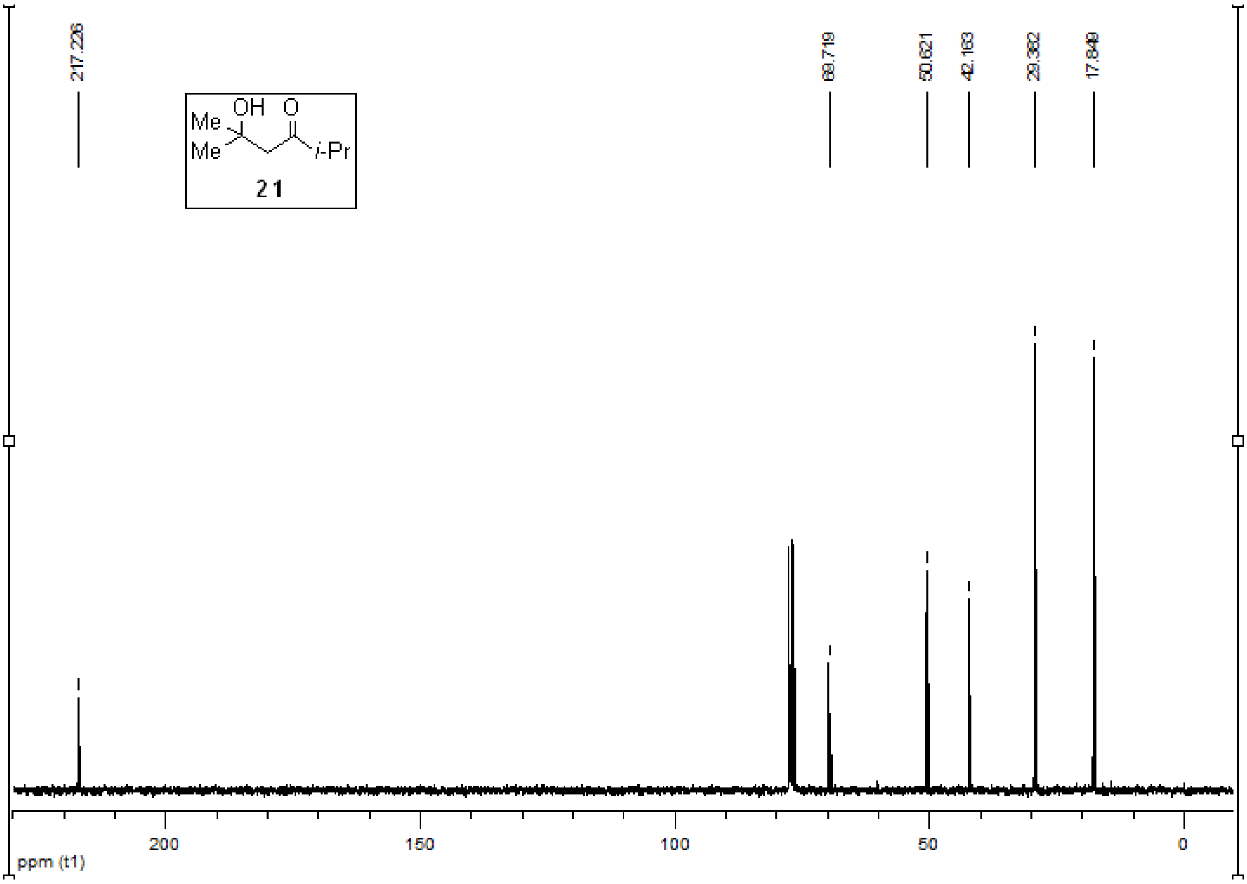
217.226
Me
OH
O
Me
i-Pr
21
69.719
50.621
42.163
29.382
17.849
200
150
100
50
0
ppm (t1)

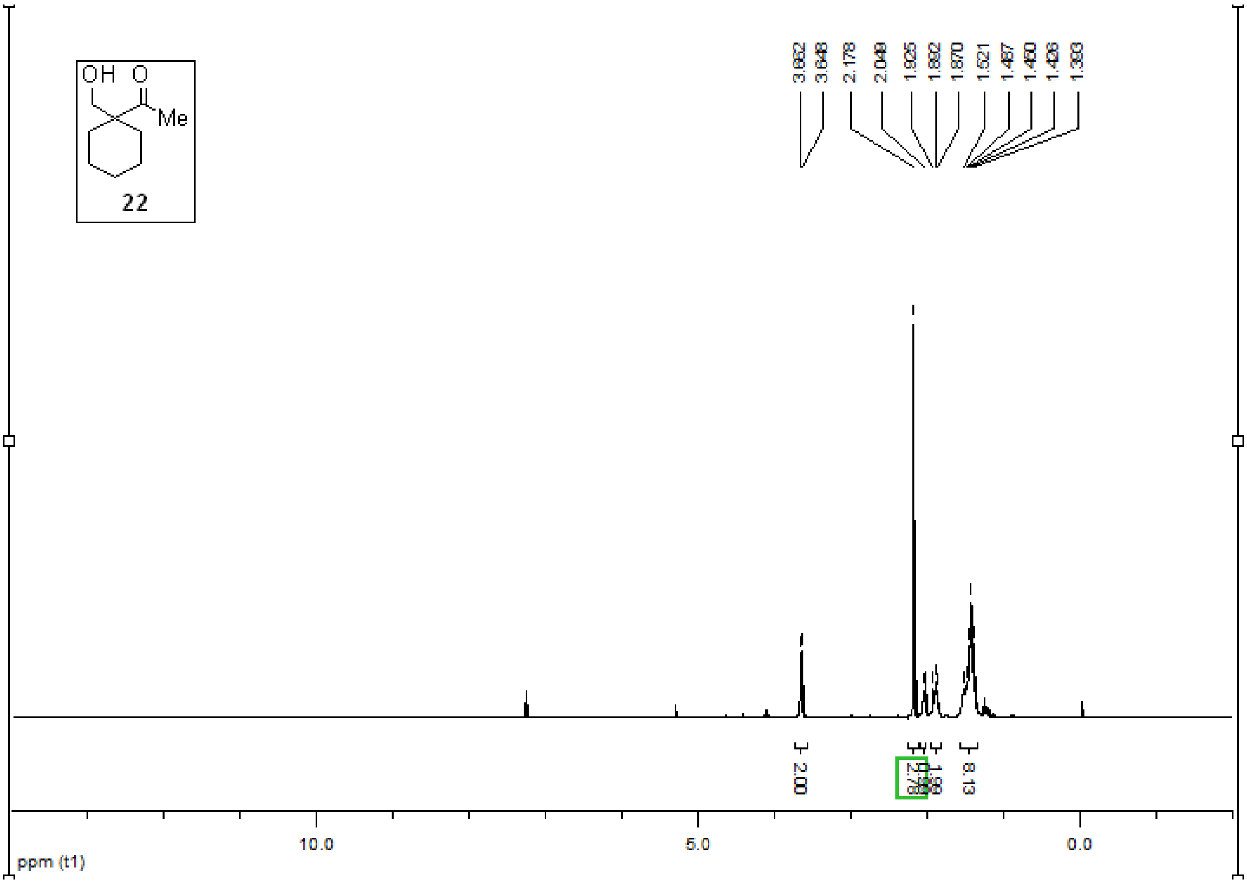
OH O
Me
22
3.662
3.648
2.178
2.049
1.925
1.892
1.870
1.521
1.487
1.450
1.426
1.393
2.00
1.88
8.13
10.0
5.0
0.0
ppm (t1)

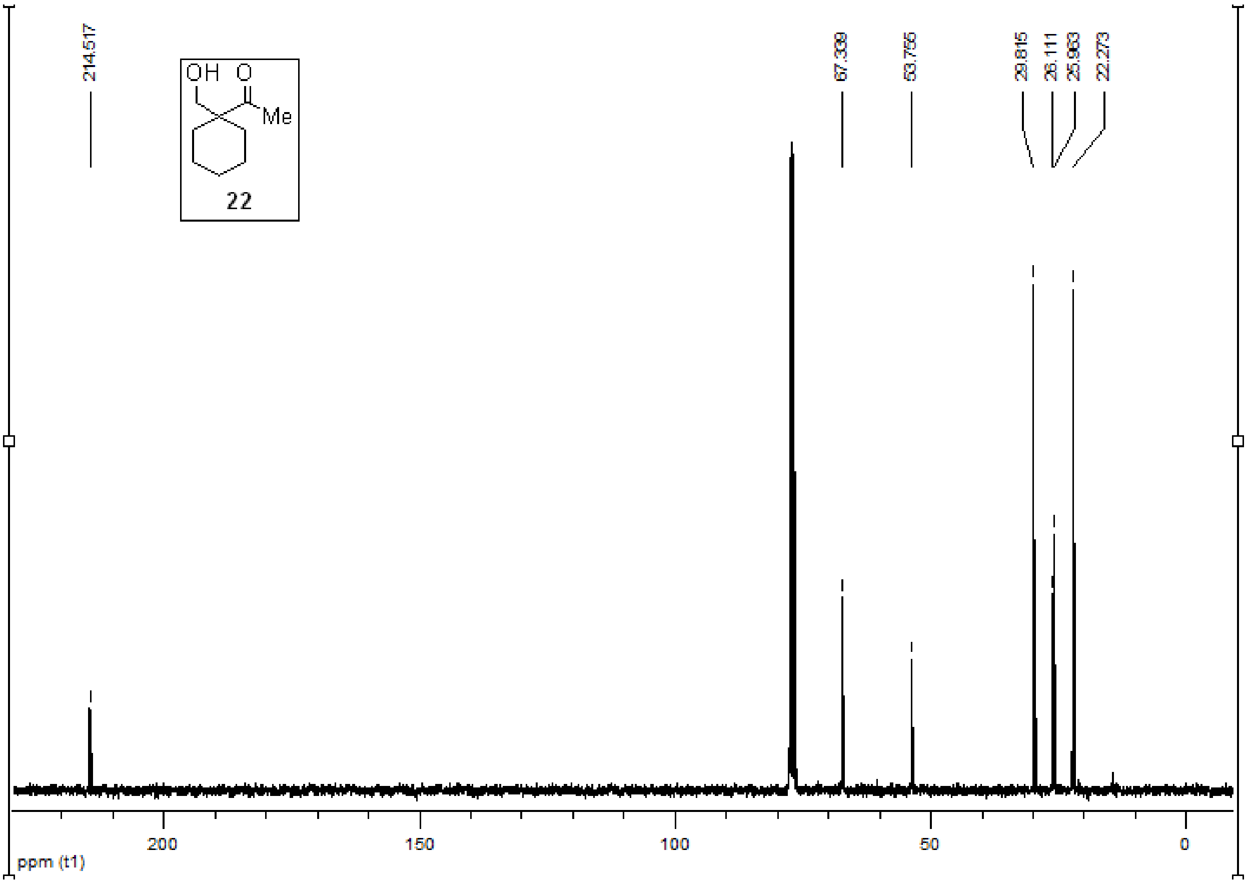
214.517
OH O
Me
22
67.339
53.765
29.815
26.111
25.963
22.273
200
150
100
50
0
ppm (t1)

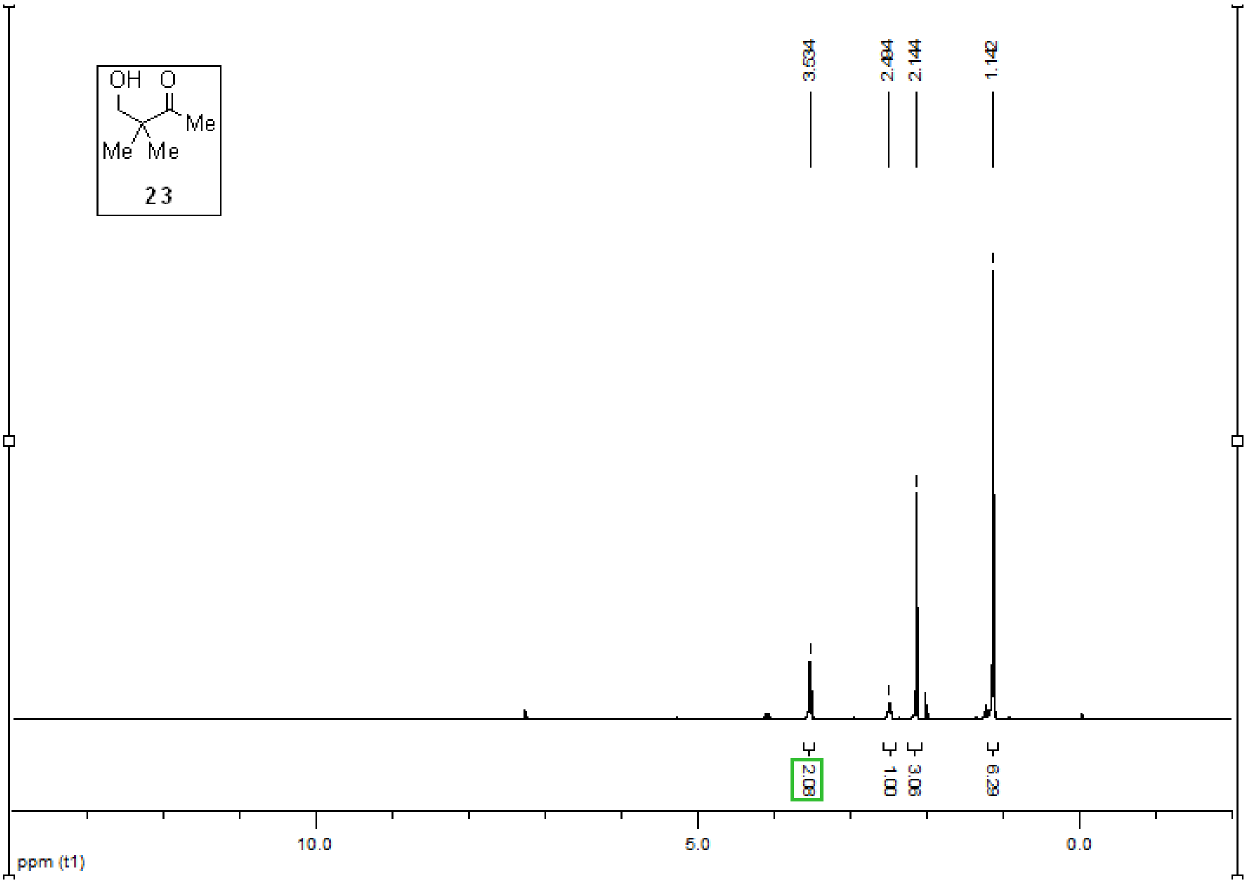
OH O
Me
Me Me
23
3.534
2.484
2.144
1.142
2.08
1.00
3.06
6.29
10.0
5.0
0.0
ppm (t1)

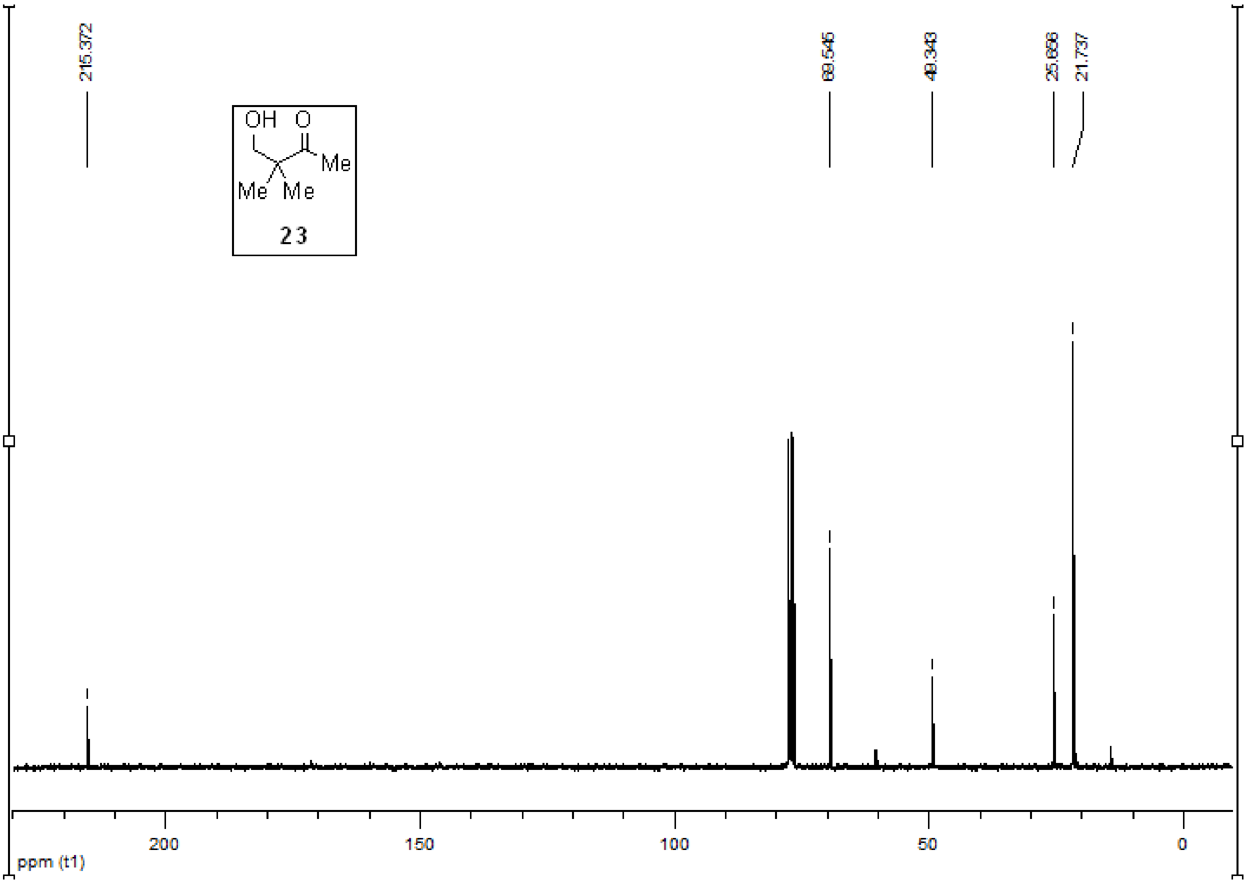
OH O
Me
Me Me
23
215.372
69.545
49.343
25.806
21.737
200
150
100
50
0
ppm (t1)

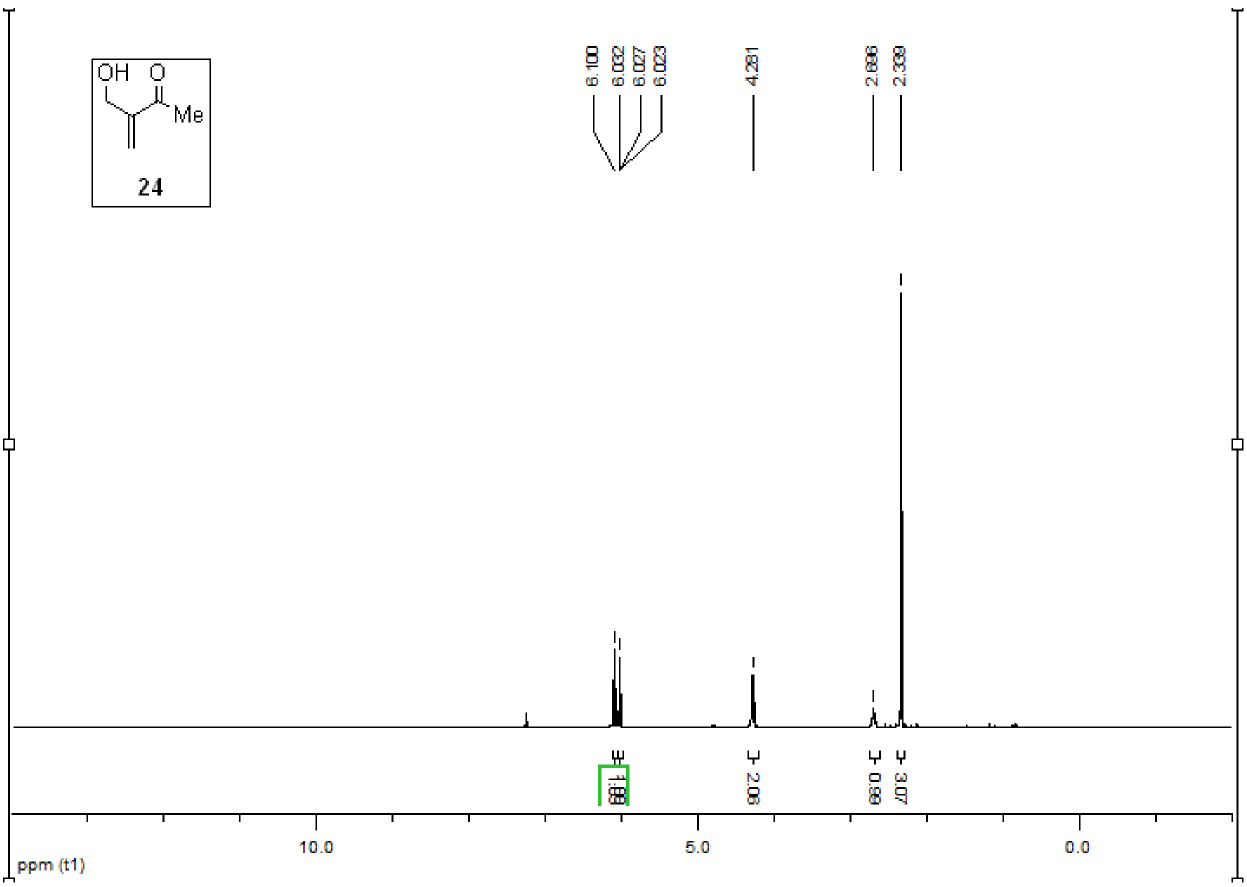

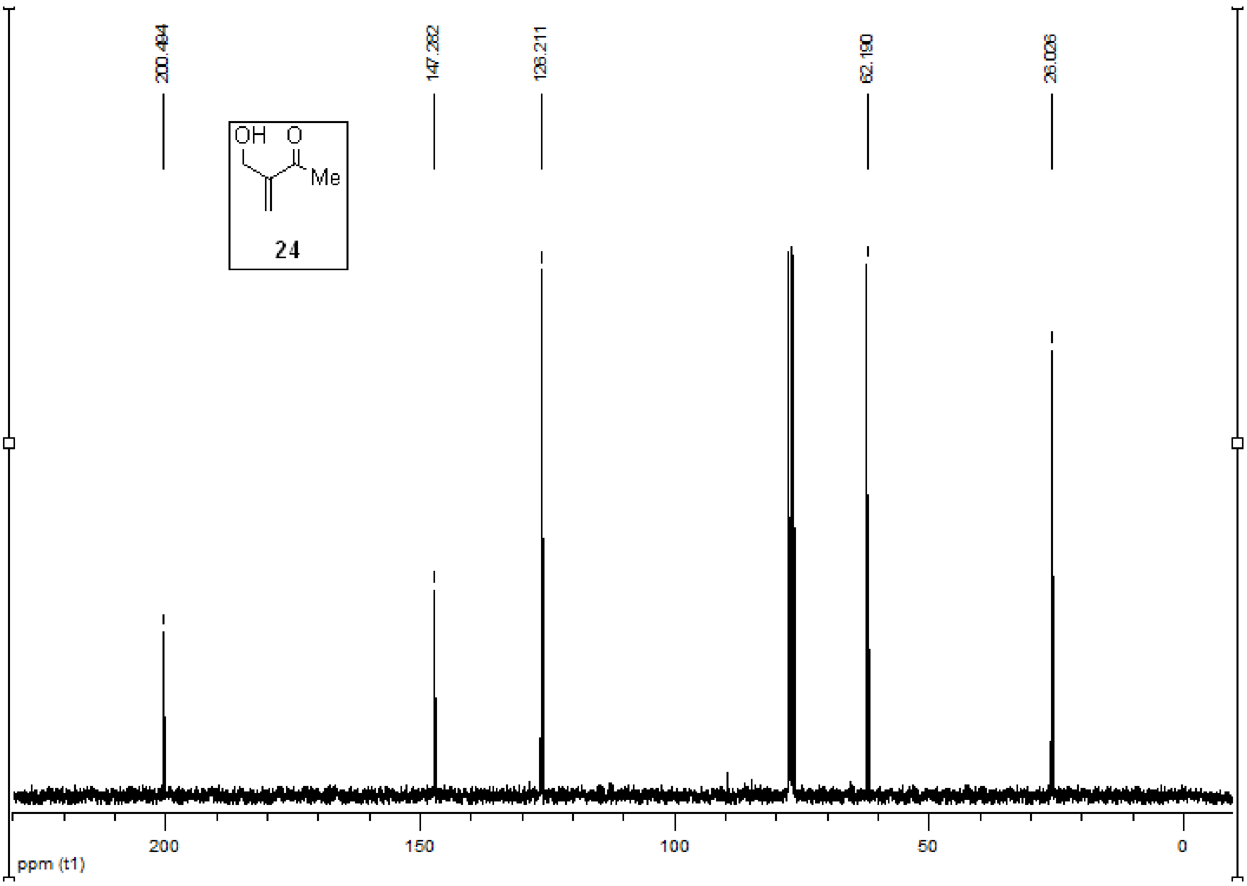

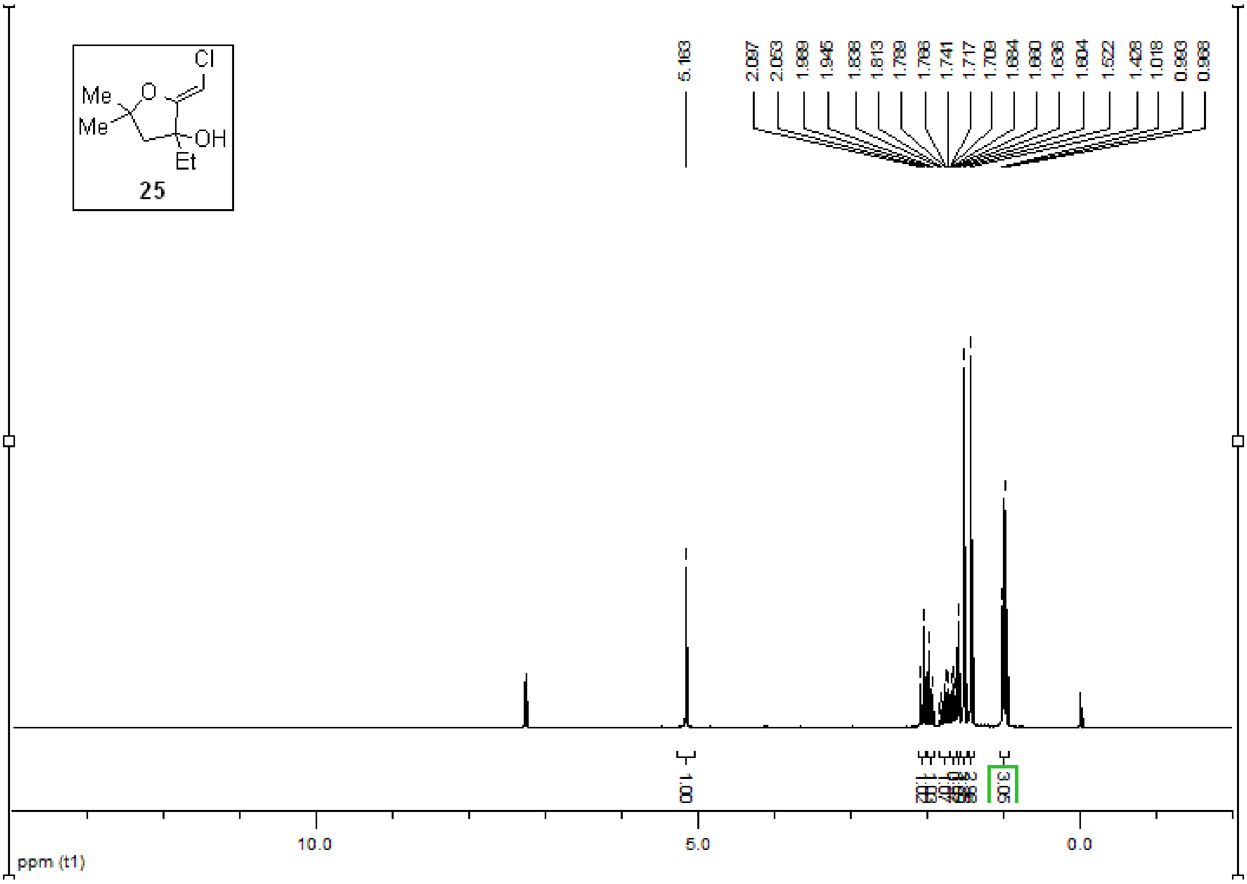
Cl
Me
O
Me
OH
Et
25
5.163
2.097
2.063
1.989
1.945
1.838
1.813
1.789
1.766
1.741
1.717
1.709
1.684
1.660
1.636
1.604
1.522
1.428
1.018
0.993
0.968
1.00
3.05
10.0
5.0
0.0
ppm (t1)

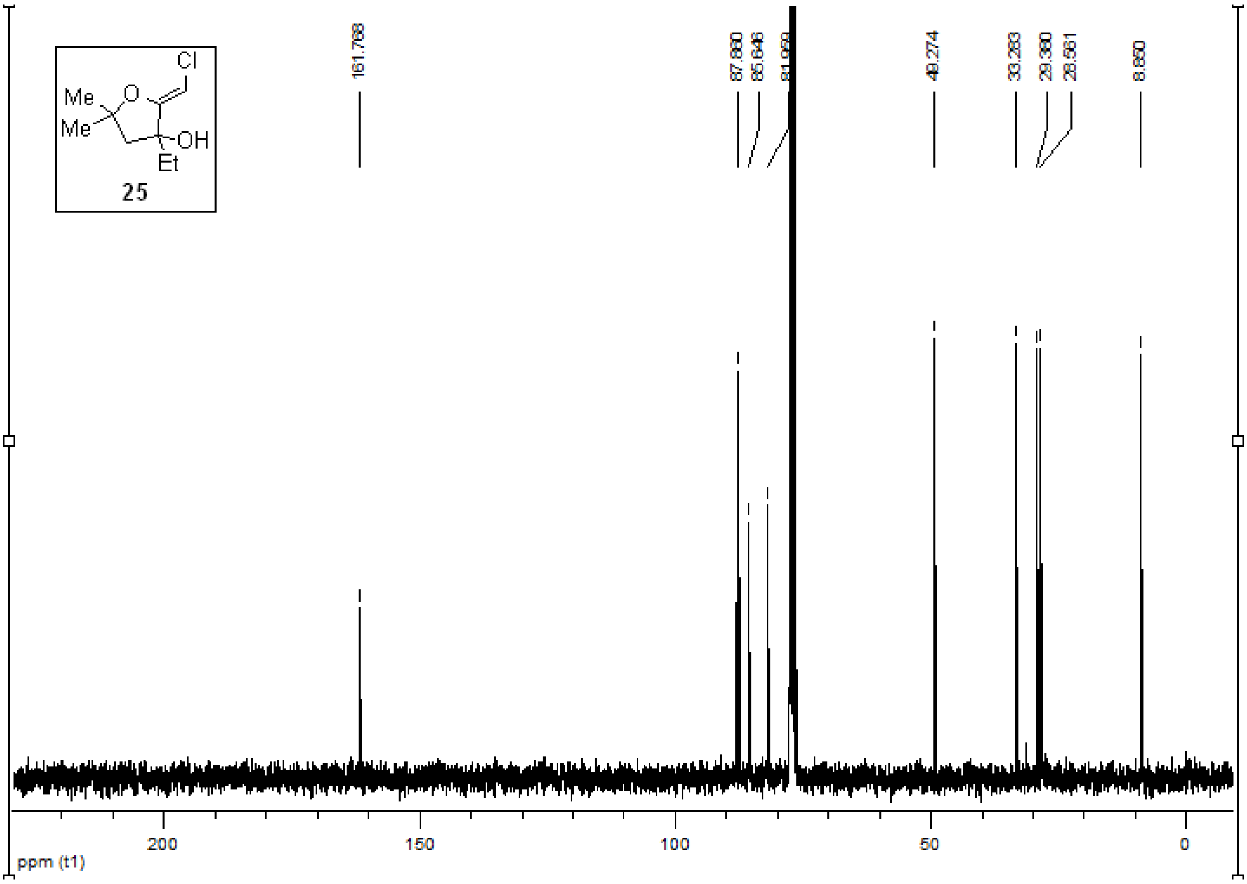
Cl
Me
O
Me
OH
Et
25
161.768
87.860
85.646
49.274
33.263
29.380
28.561
8.850
200
150
100
50
0
ppm (t1)

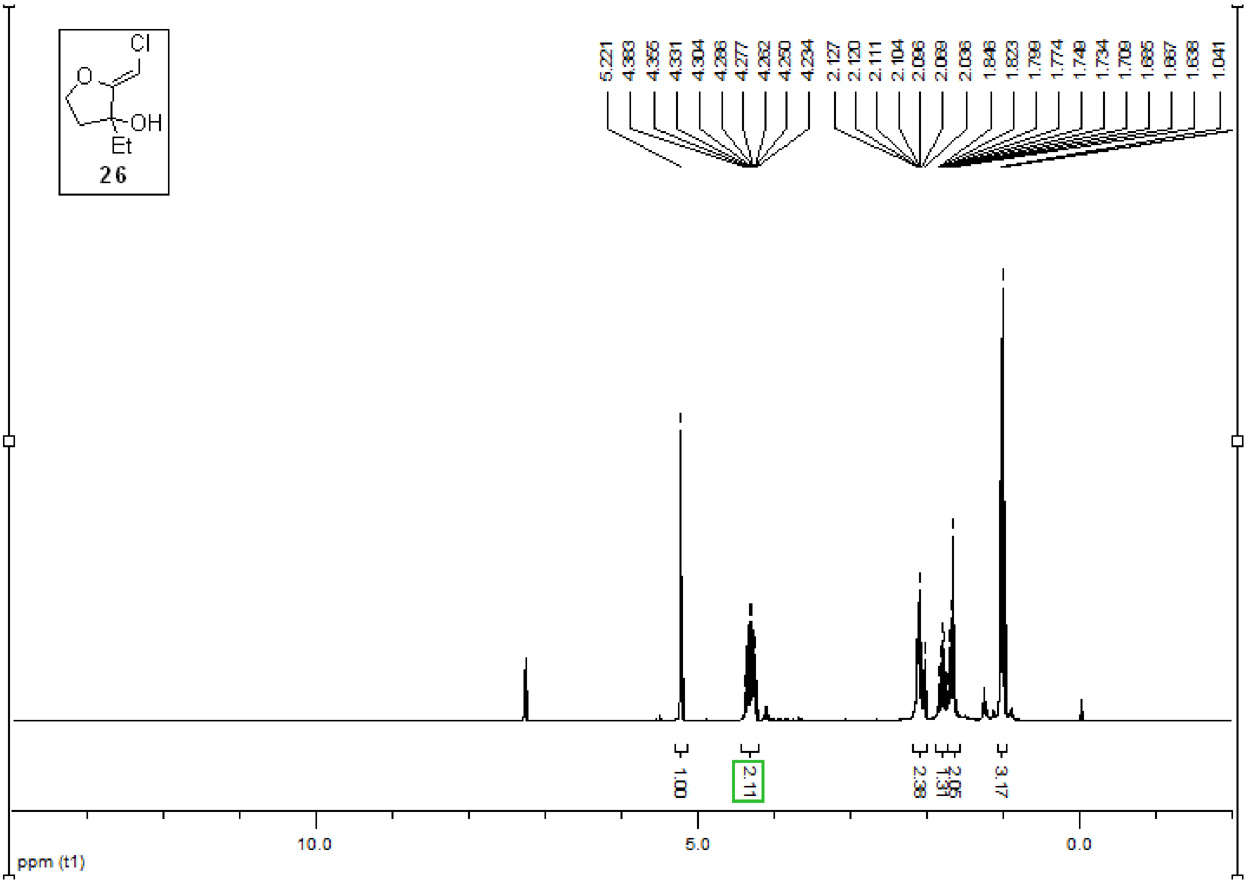

Cl
O
OH
Et
26
5.221
4.383
4.355
4.331
4.304
4.286
4.277
4.262
4.250
4.234
2.127
2.120
2.111
2.104
2.096
2.089
2.036
1.846
1.823
1.799
1.774
1.749
1.734
1.709
1.685
1.667
1.638
1.041
1.00
2.11
2.38
1.31
2.05
3.17
10.0
5.0
0.0
ppm (t1)

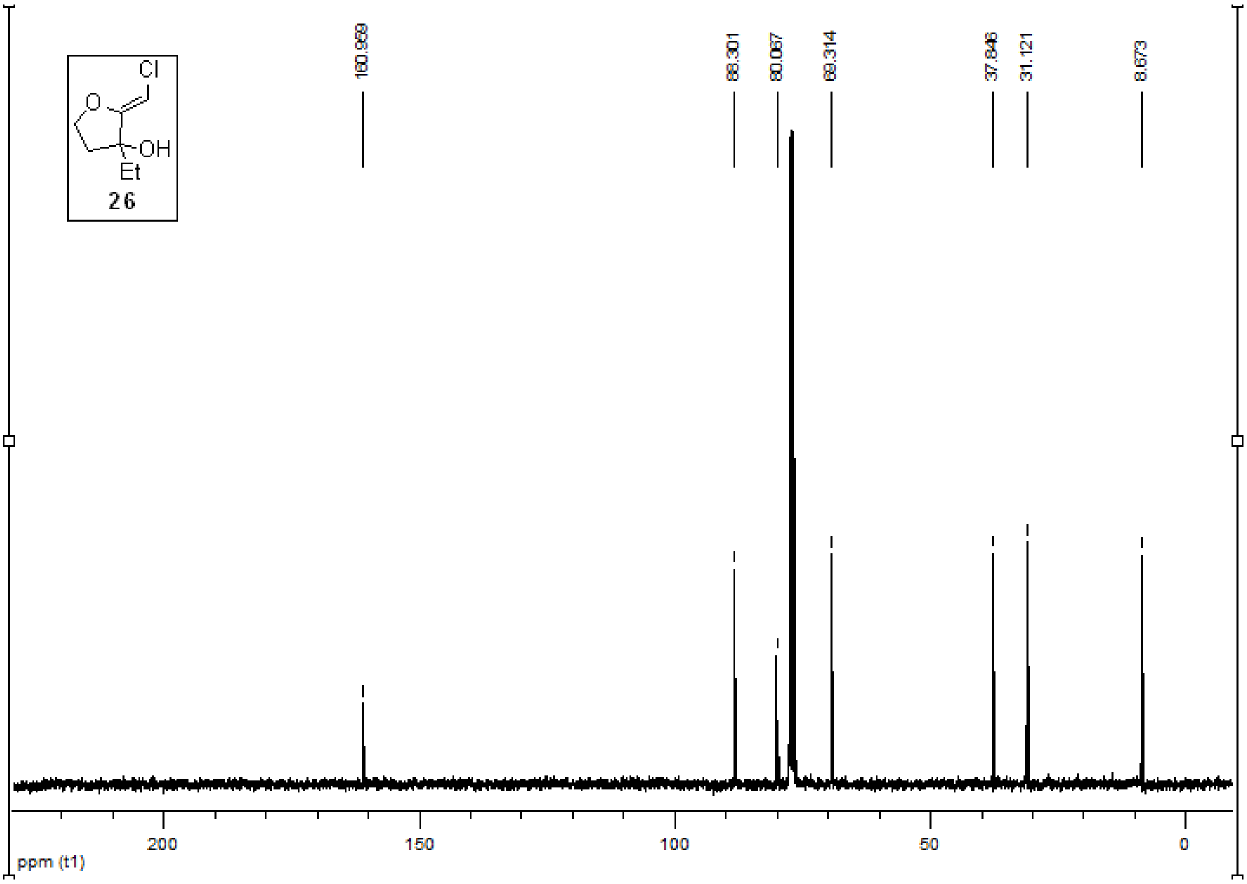

Cl
O
OH
Et
26
160.959
88.301
80.067
69.314
37.846
31.121
8.673
200
150
100
50
0
ppm (t1)

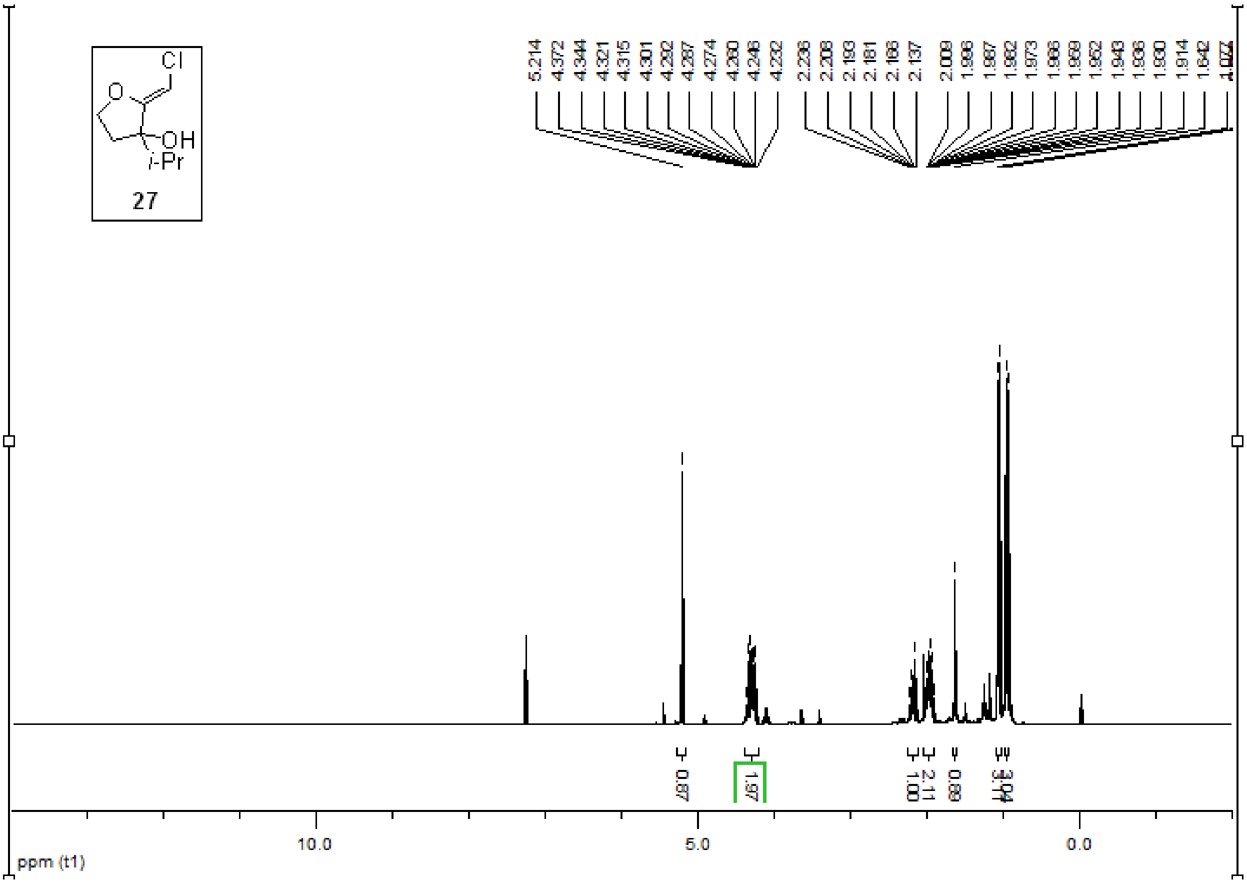

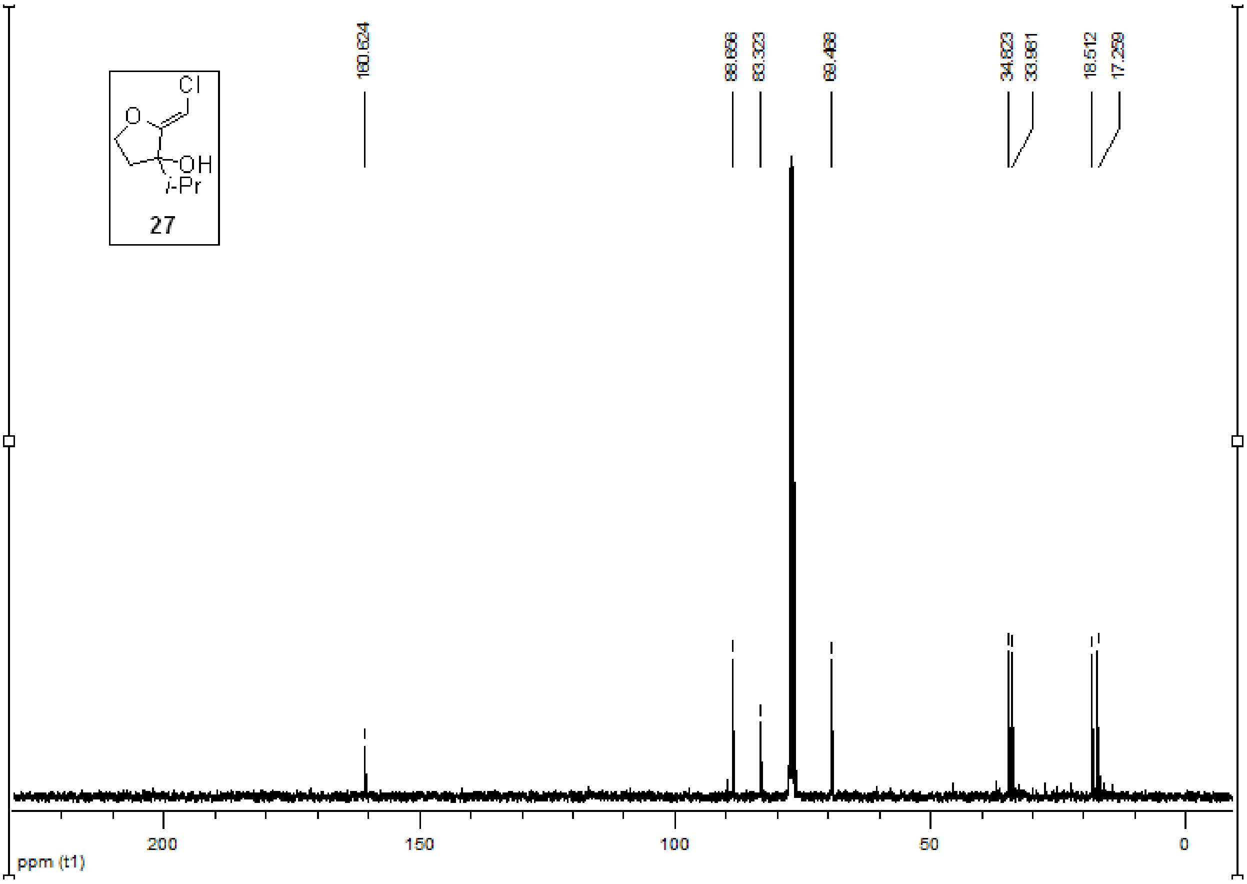

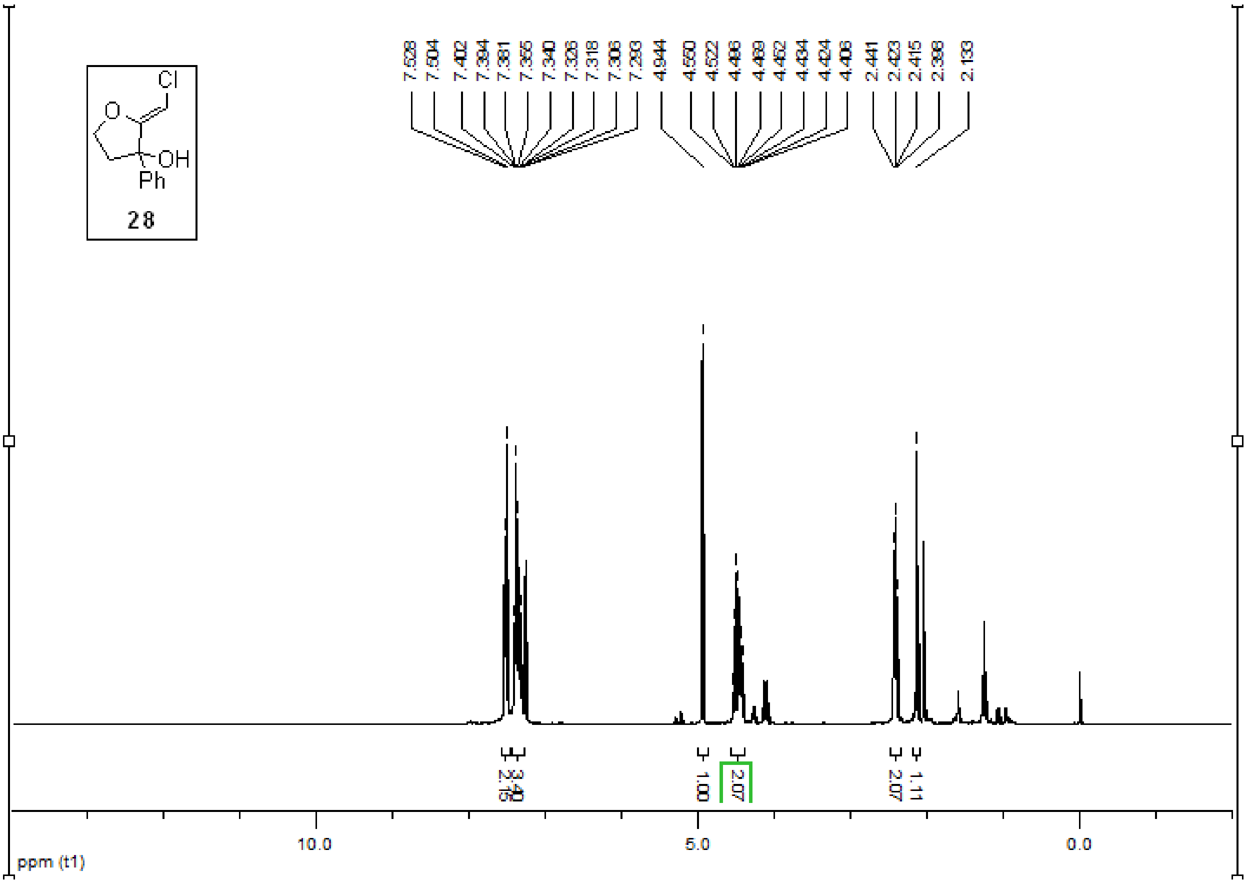

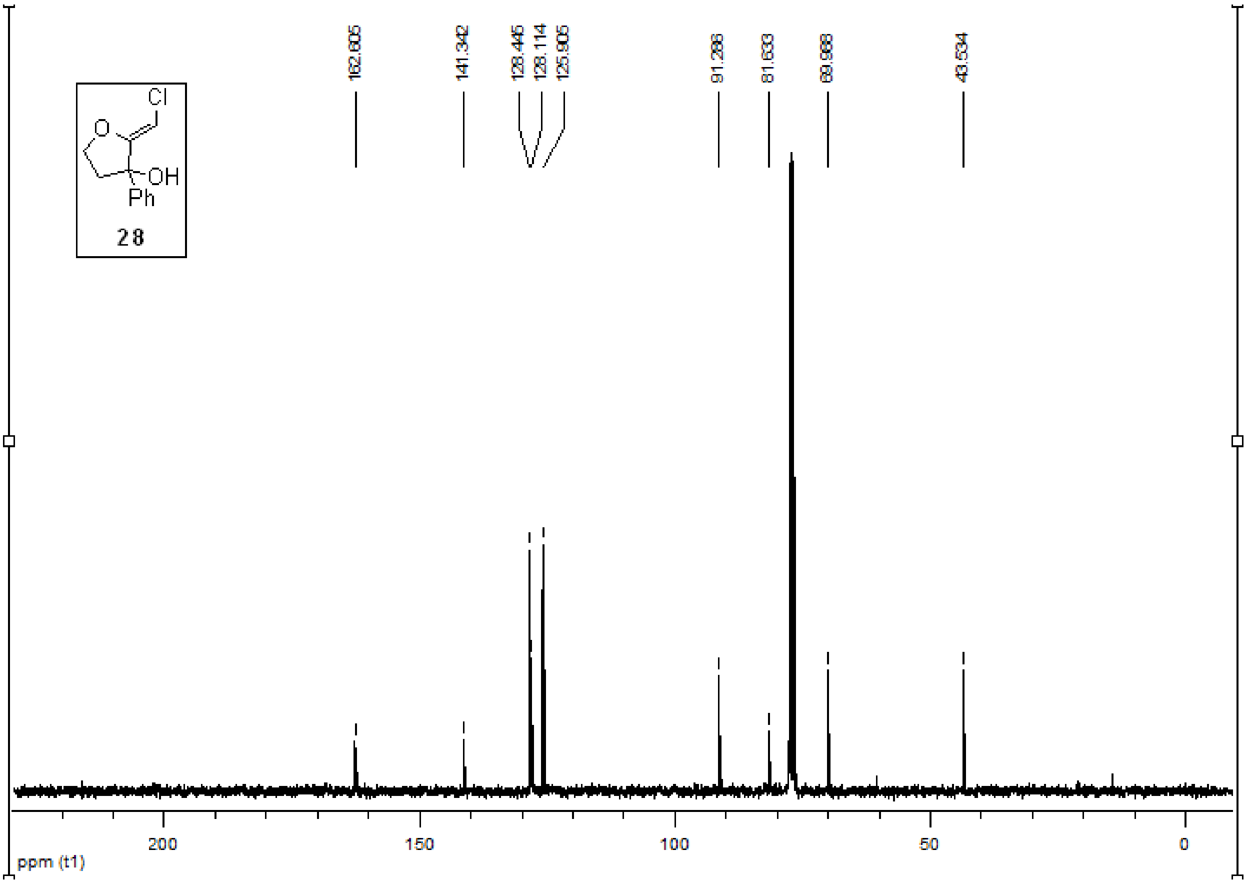

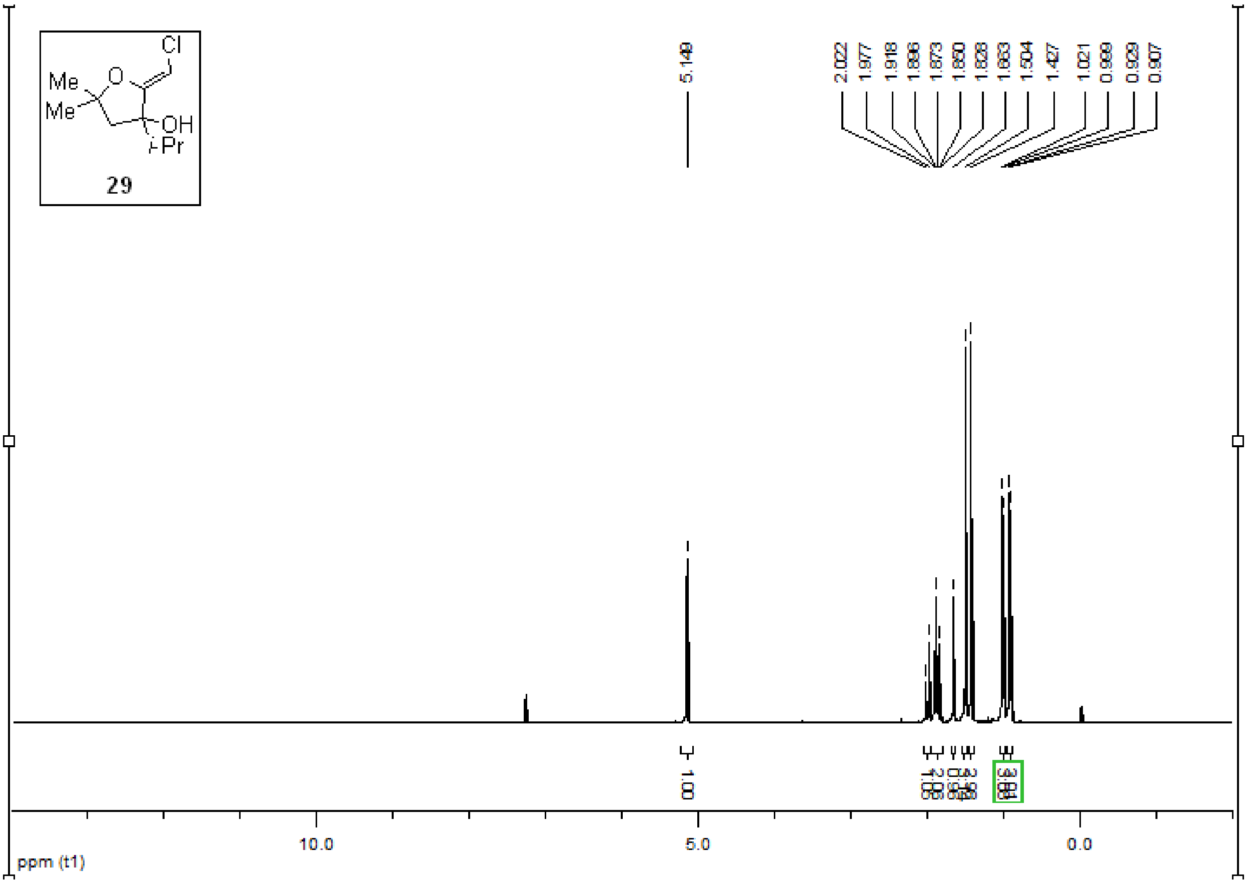

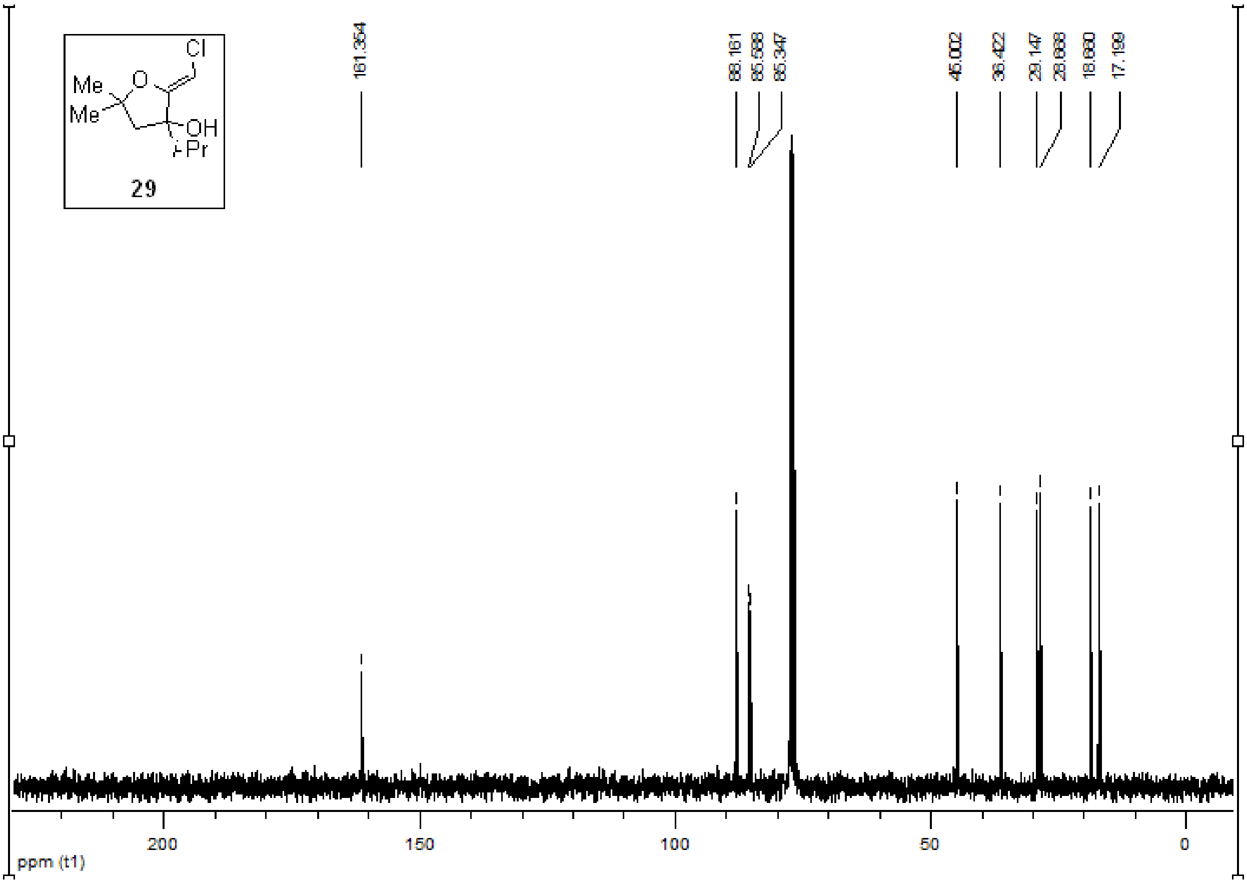

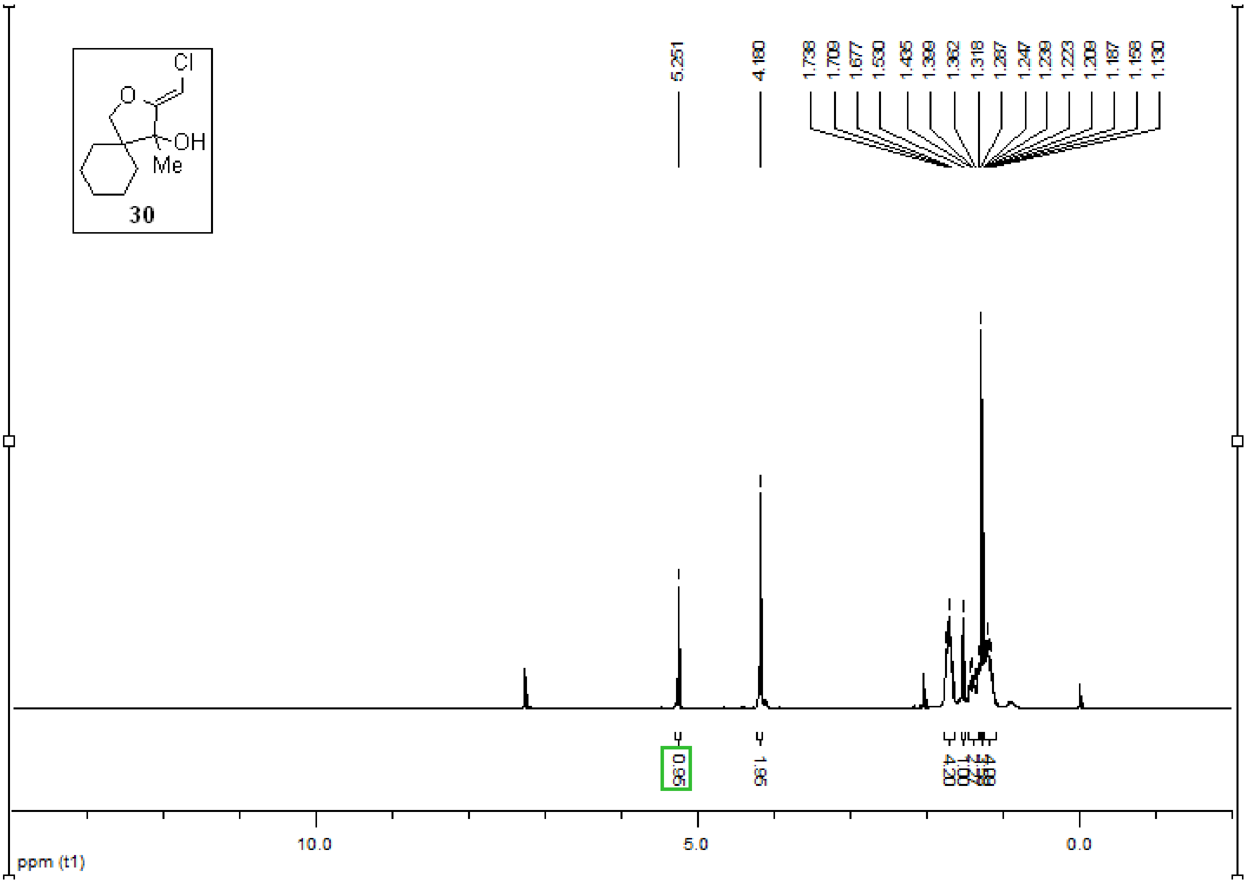
Cl
O
OH
Me
30
5.251
4.180
1.738
1.709
1.677
1.530
1.435
1.399
1.362
1.318
1.287
1.247
1.239
1.223
1.209
1.187
1.158
1.130
0.95
1.95
4.20
10.0
5.0
0.0
ppm (t1)

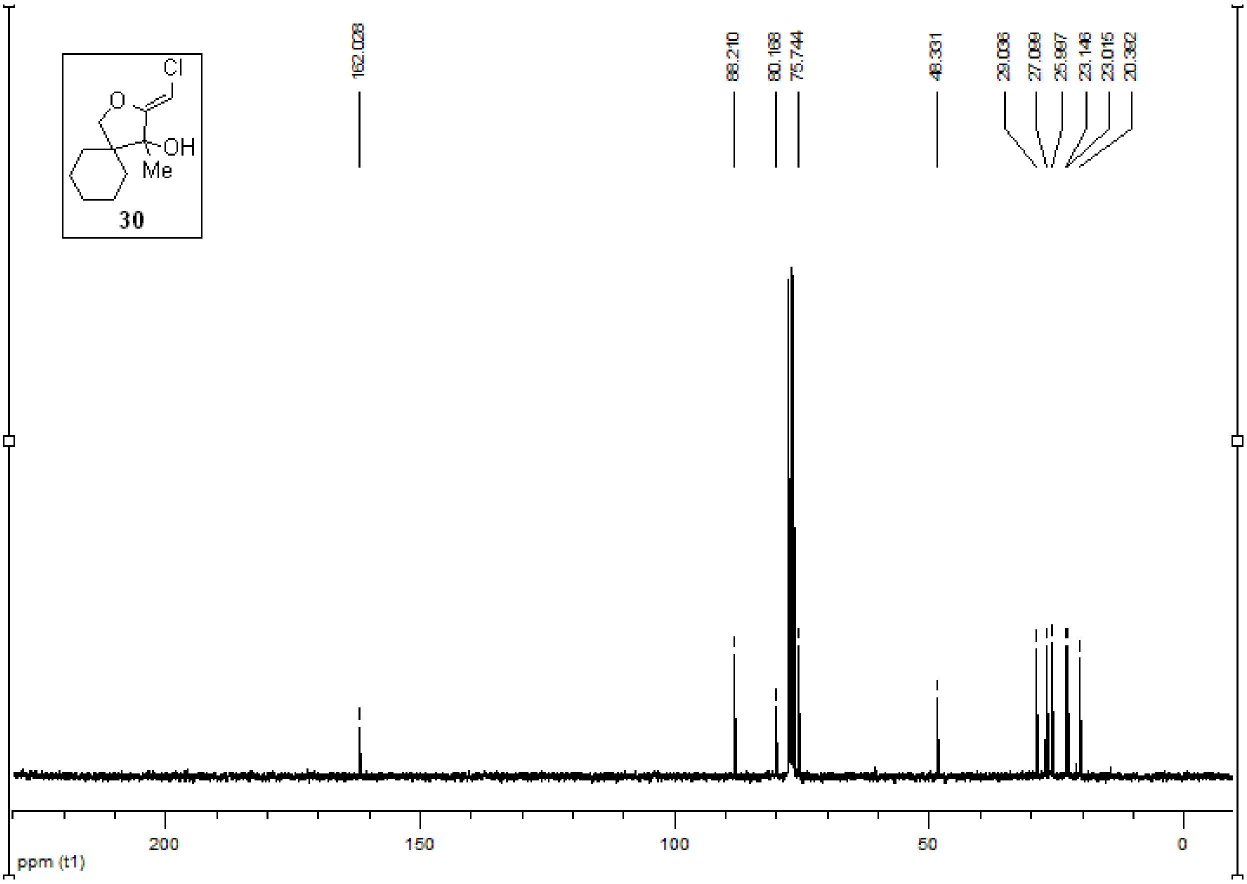
Cl
O
OH
Me
30
162.028
88.210
80.188
75.744
48.331
29.036
27.088
25.987
23.146
23.015
20.382
200
150
100
50
0
ppm (t1)

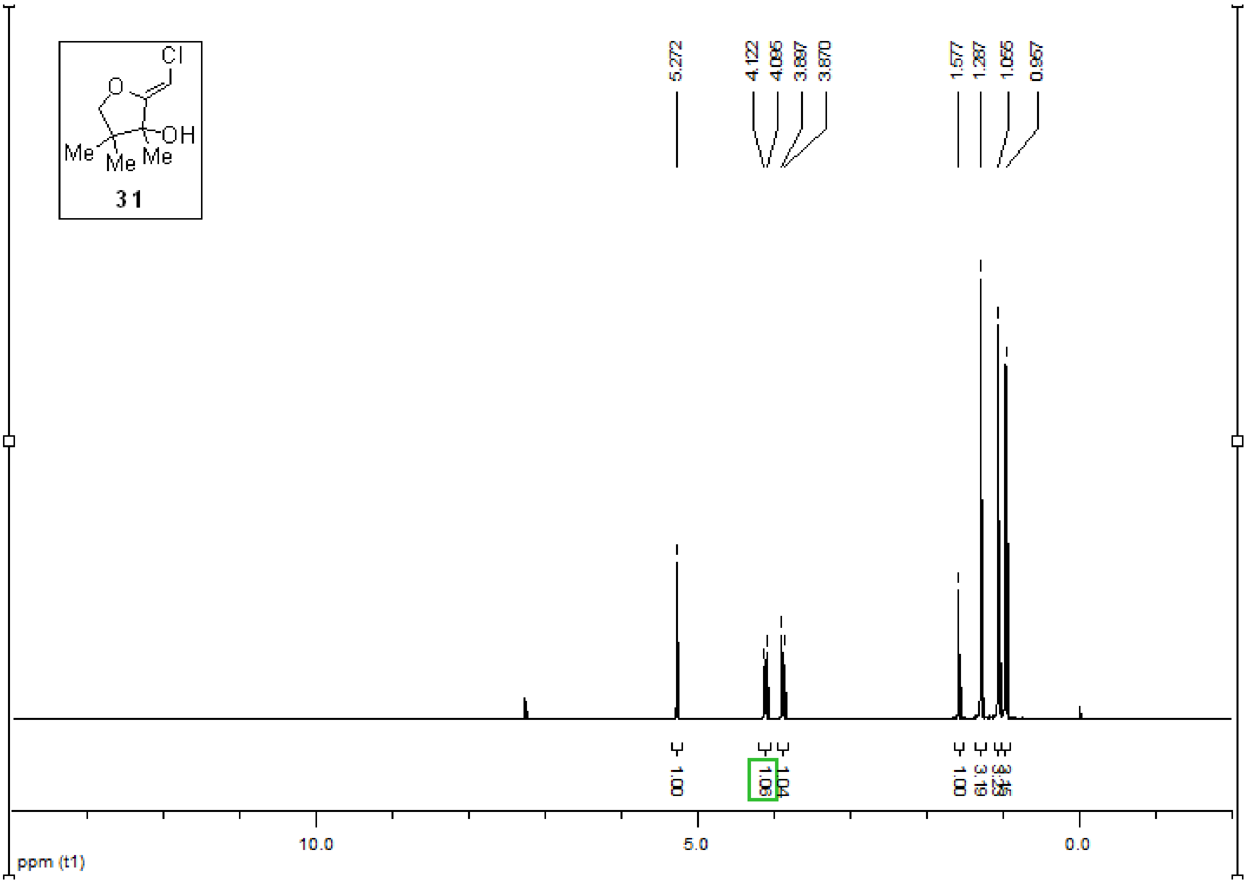

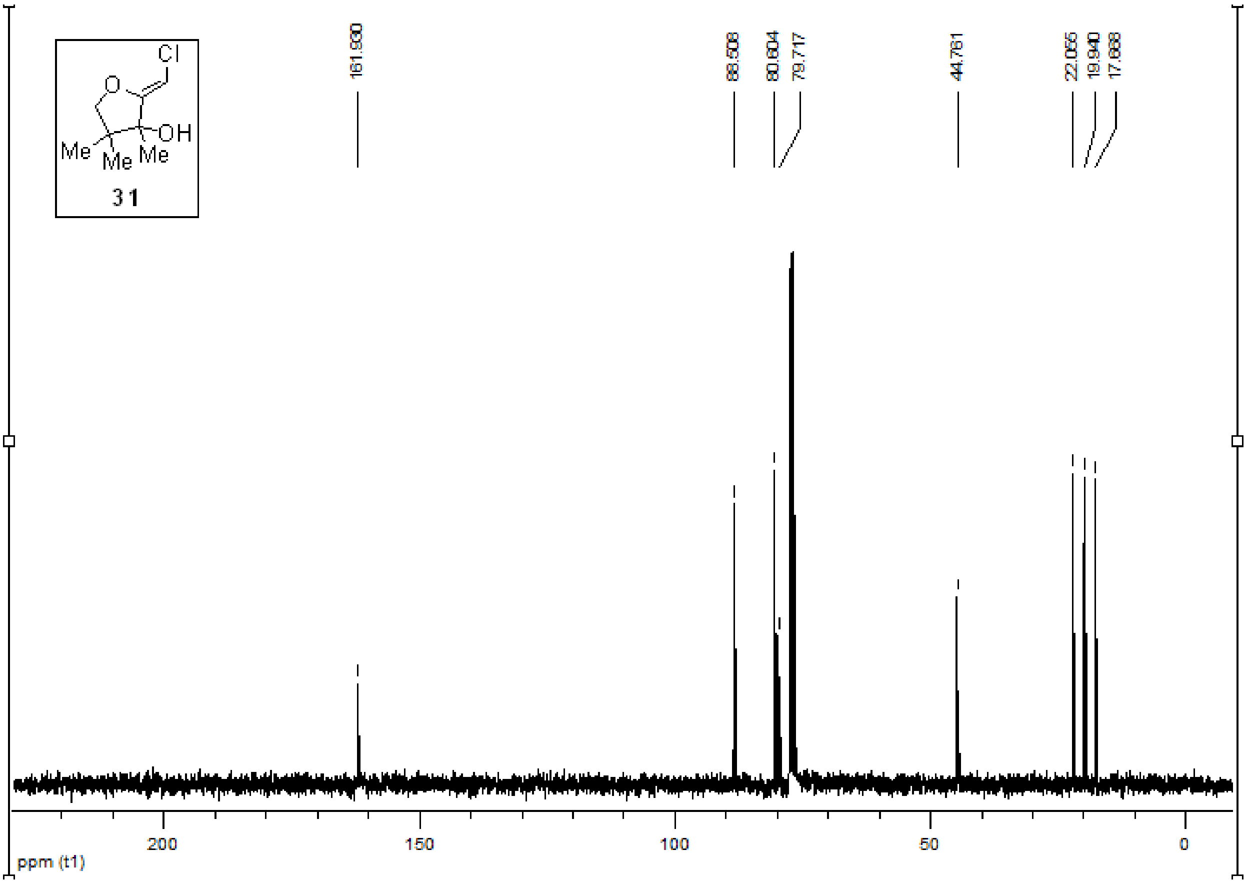

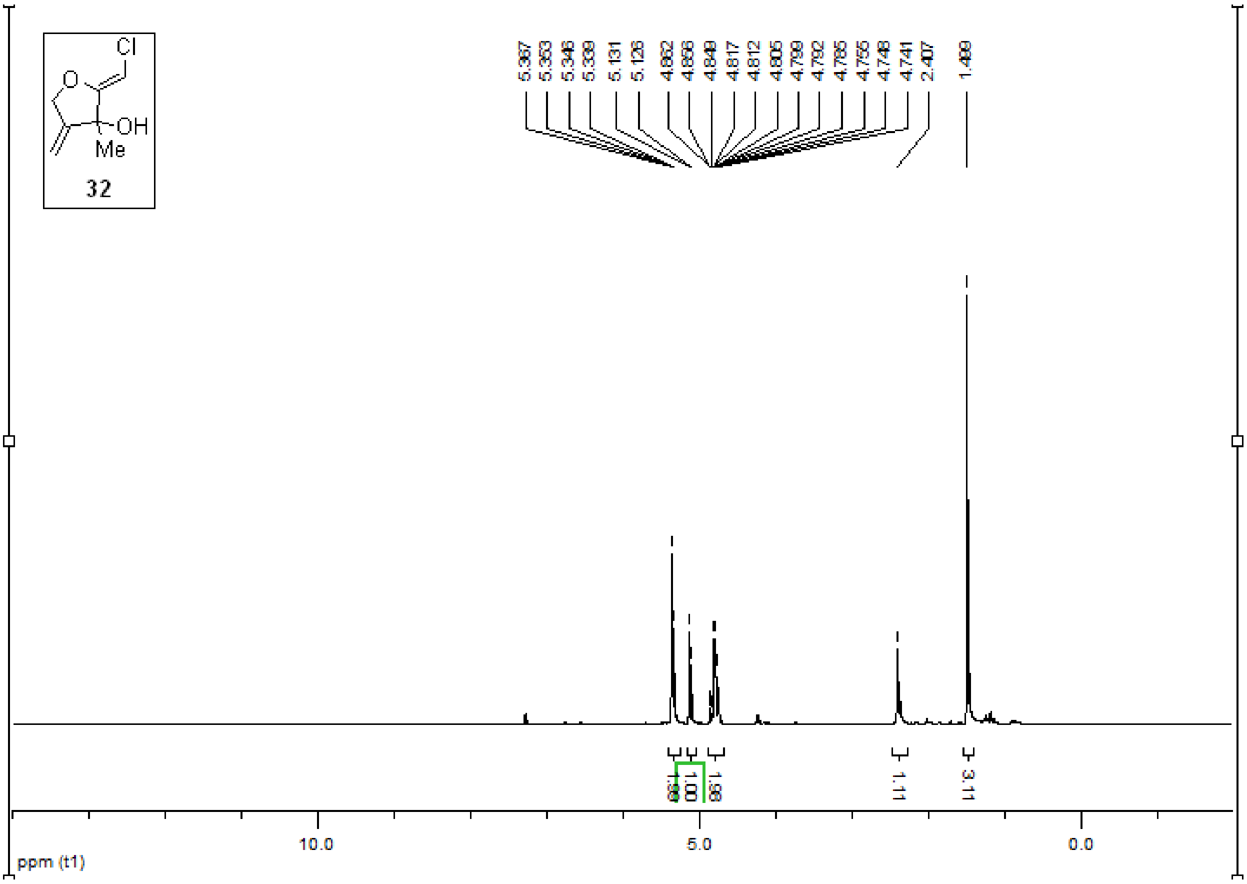
Cl
O
OH
Me
32
5.367
5.353
5.346
5.339
5.131
5.126
4.862
4.856
4.849
4.817
4.812
4.805
4.799
4.792
4.785
4.755
4.748
4.741
2.407
1.499
1.11
3.11
10.0
5.0
0.0
ppm (t1)

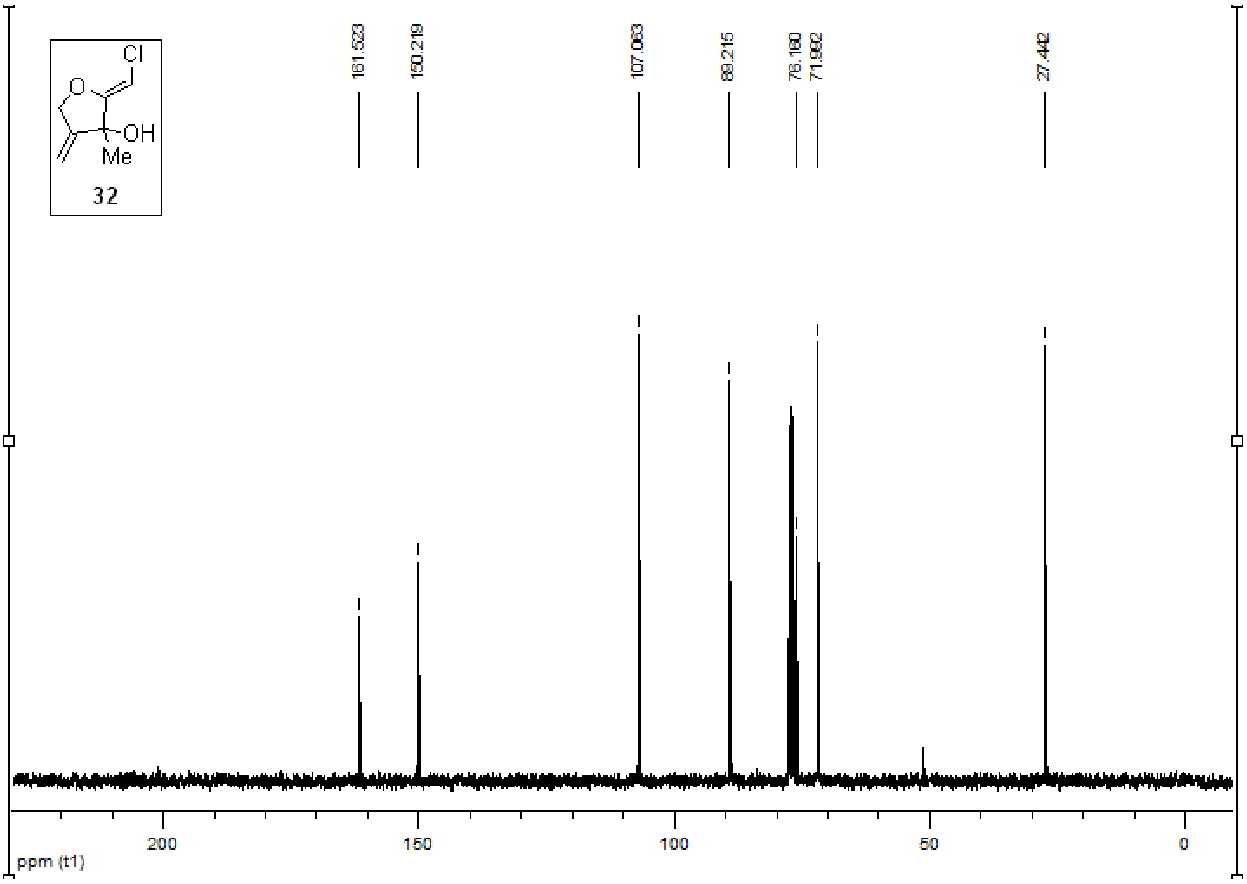
Cl
O
OH
Me
32
161.523
150.219
107.063
89.215
76.160
71.992
27.442
200
150
100
50
0
ppm (t1)

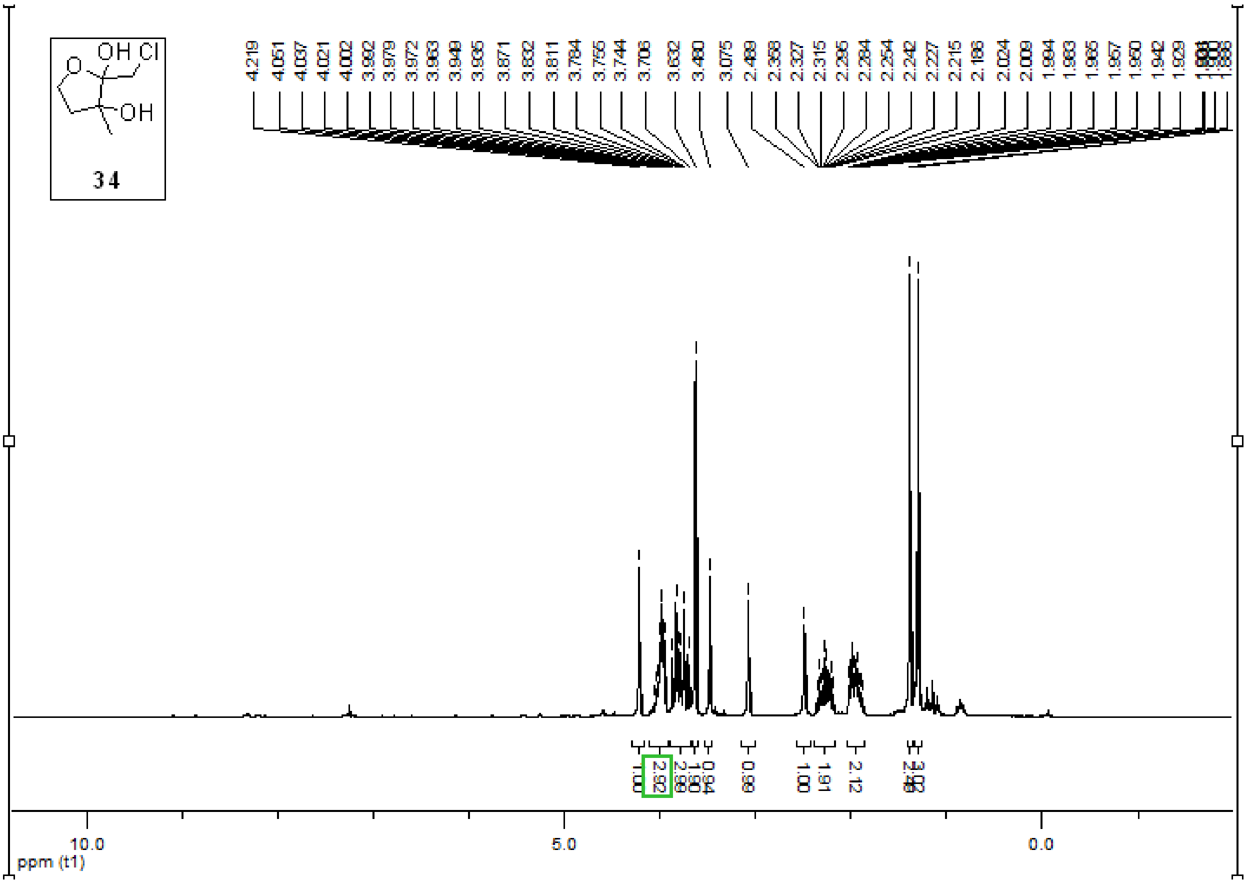

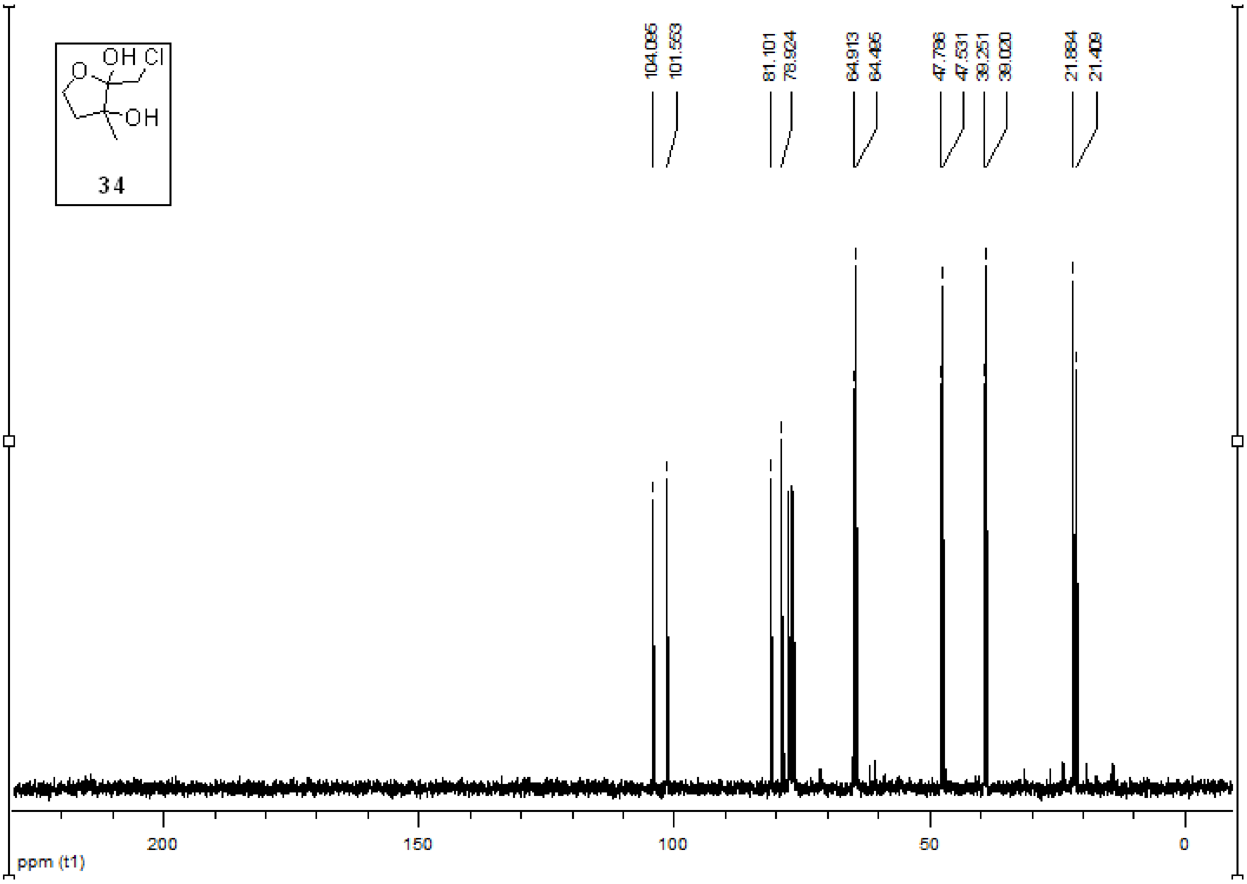

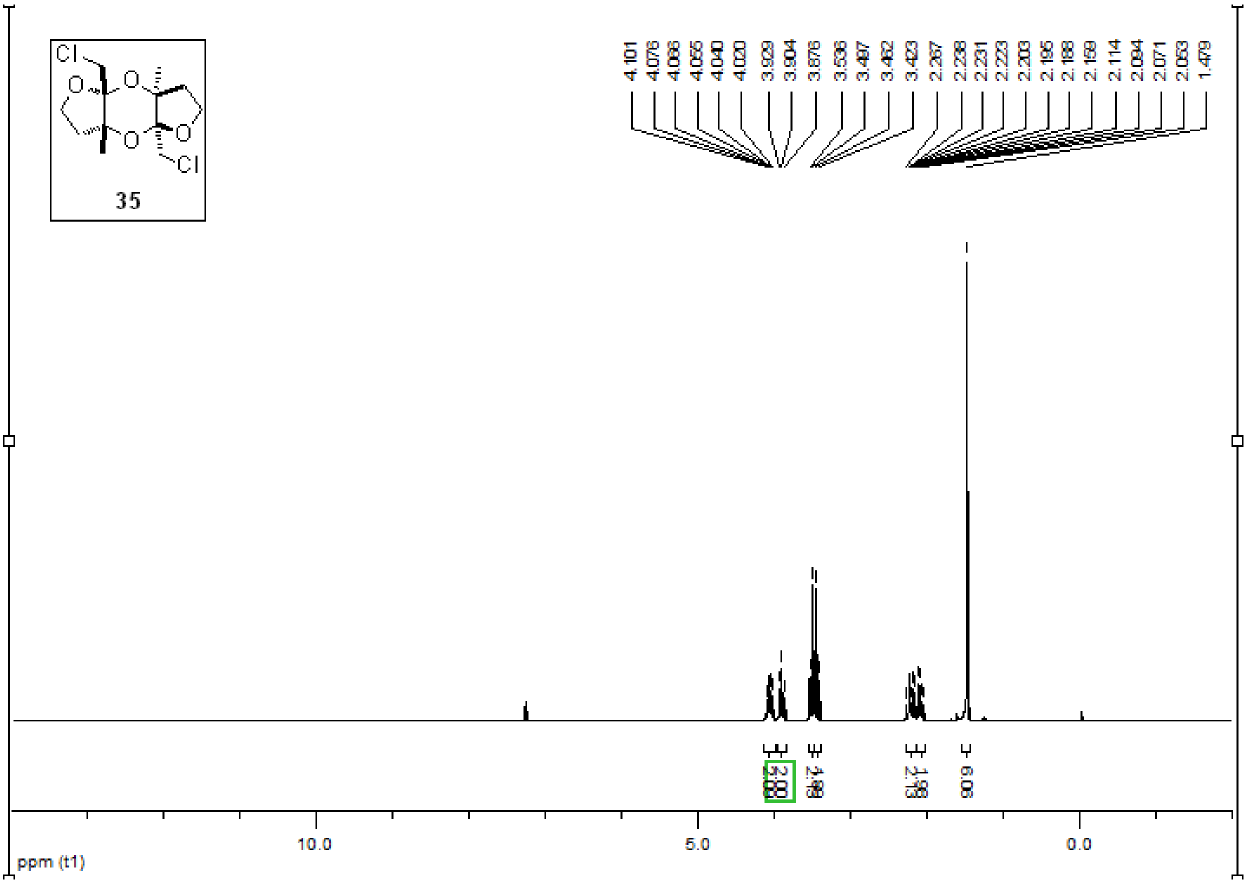

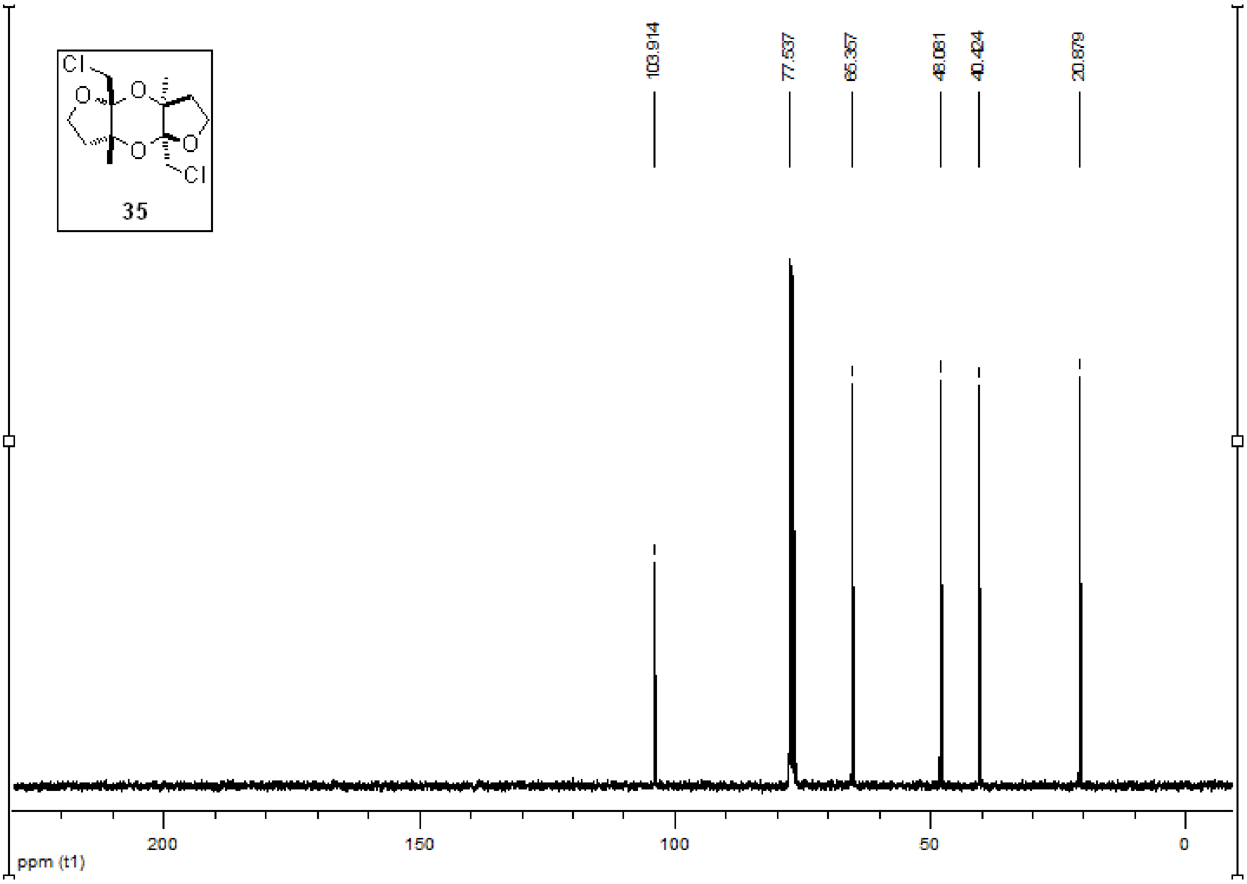

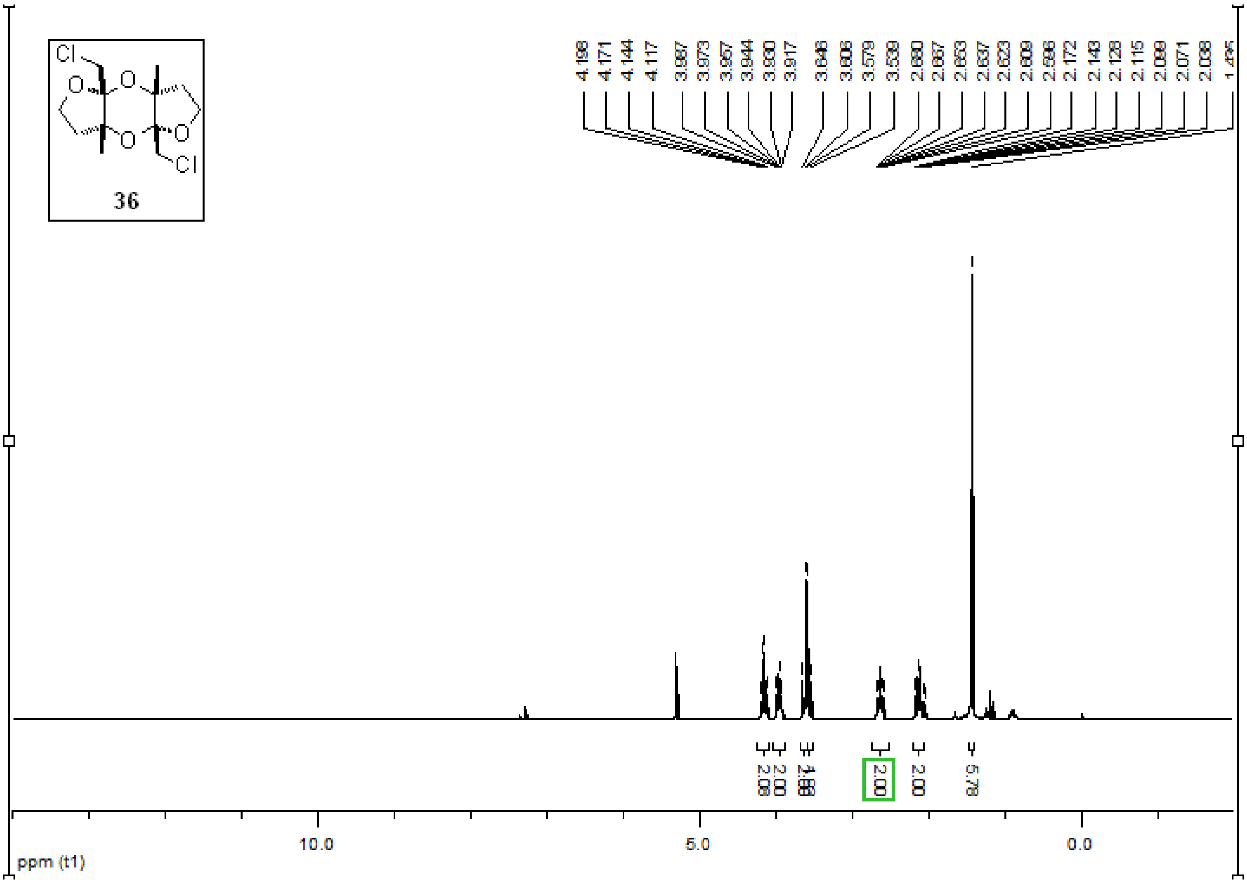
36
4.198 4.171 4.144 4.117 3.987 3.973 3.957 3.944 3.930 3.917 3.646 3.606 3.579 3.539 2.680 2.667 2.653 2.637 2.623 2.609 2.596 2.172 2.143 2.128 2.115 2.099 2.071 2.038
2.08 2.00 4.08 2.00 2.00 5.78
10.0
5.0
0.0
ppm (t1)

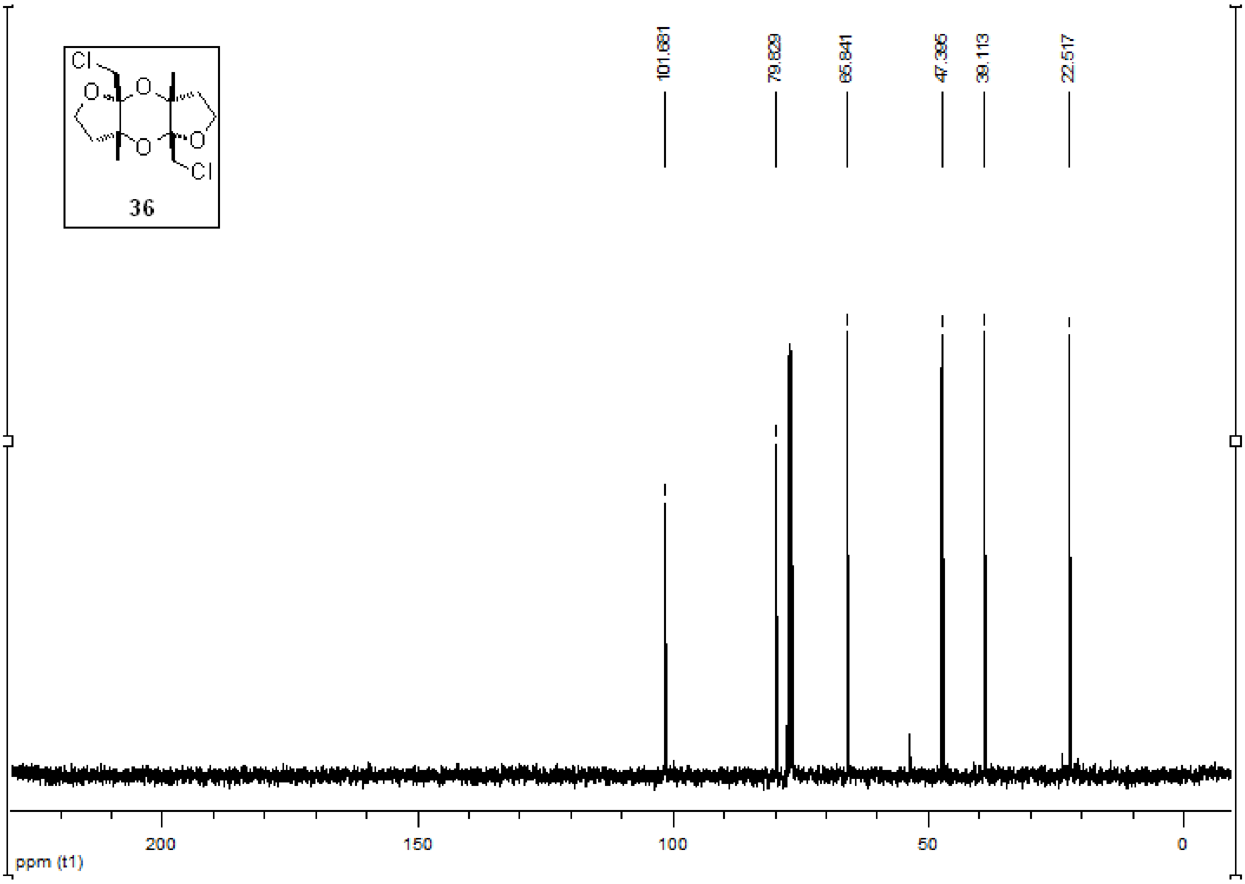
36
101.681
79.829
65.841
47.305
39.113
22.517
200
150
100
50
0
ppm (t1)

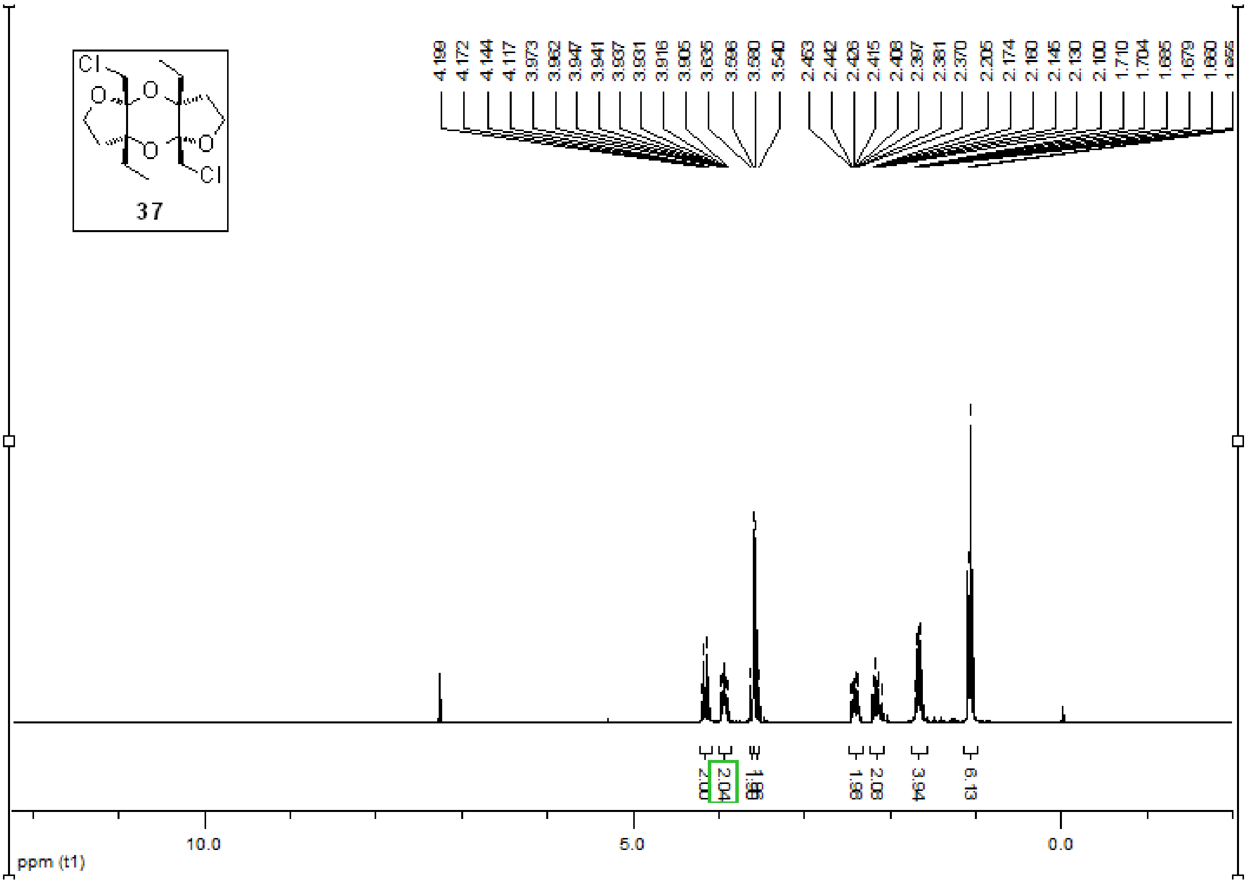
37
ppm (t1)
10.0
5.0
0.0

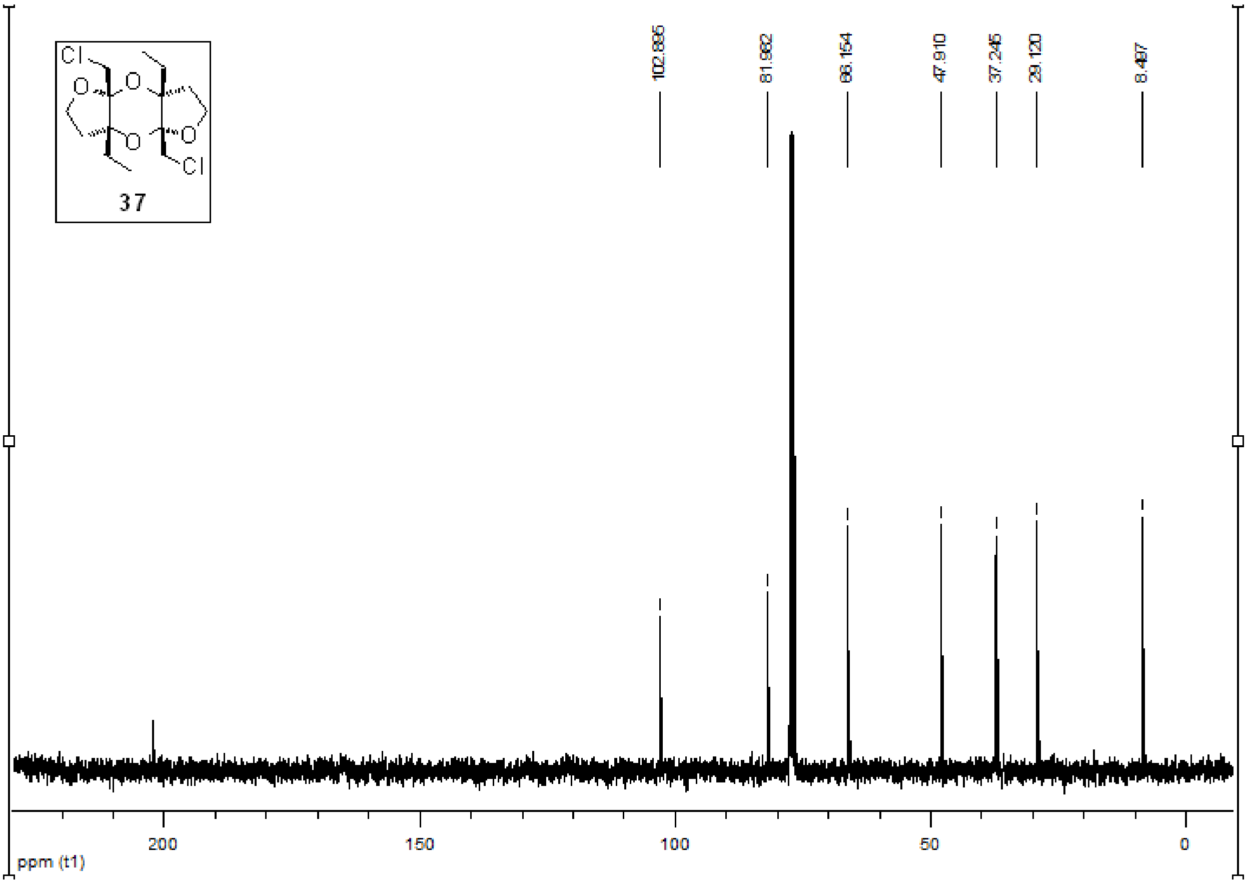
37
102.885
81.982
66.154
47.910
37.245
29.120
8.497
ppm (t1)
200
150
100
50
0

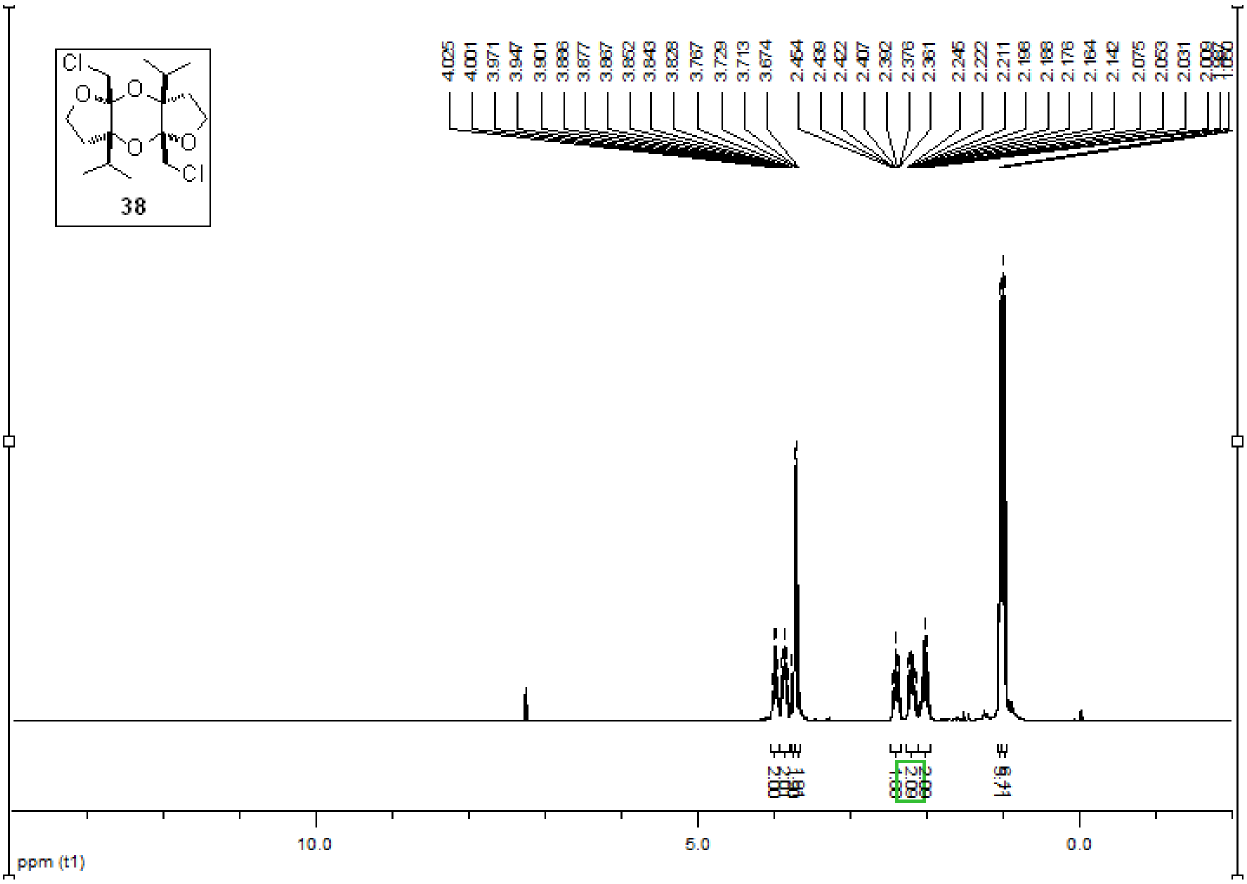

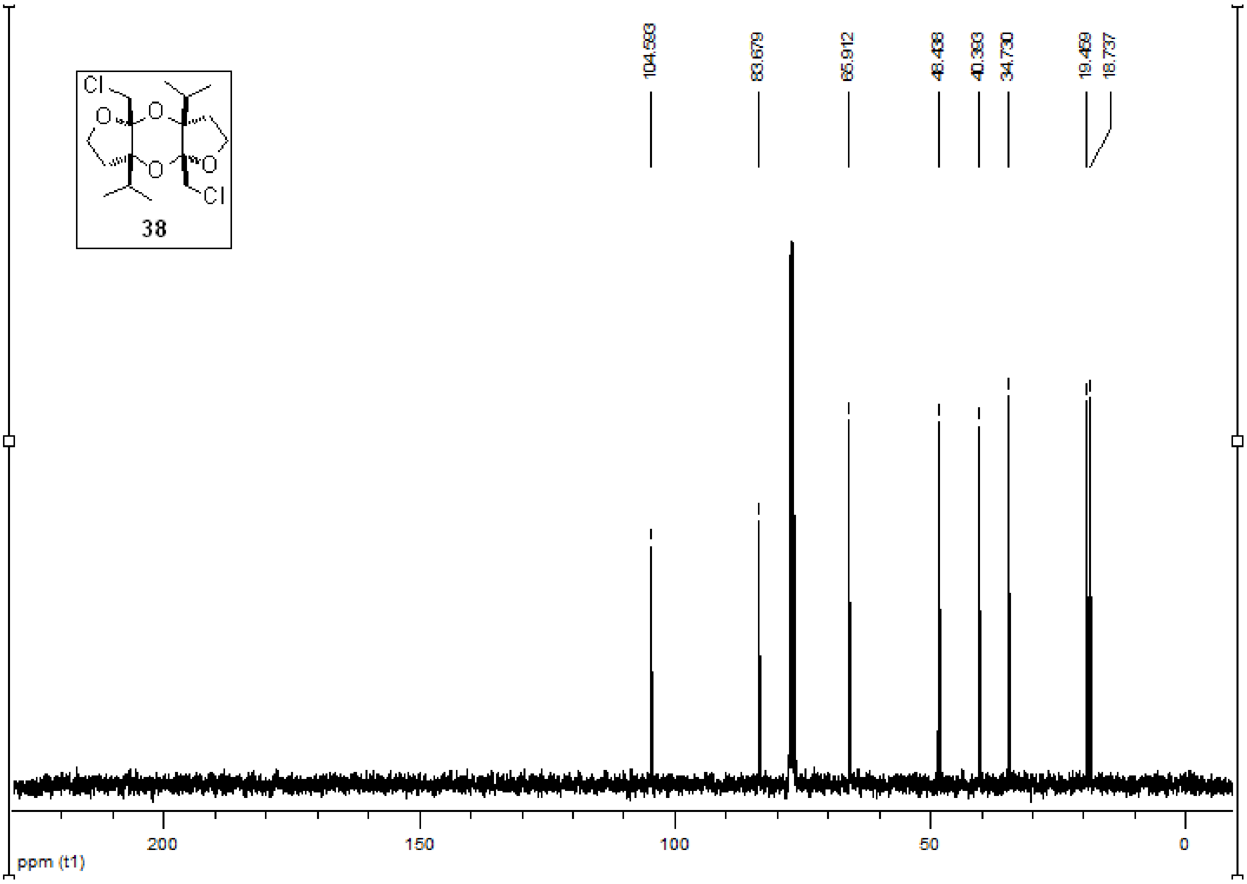

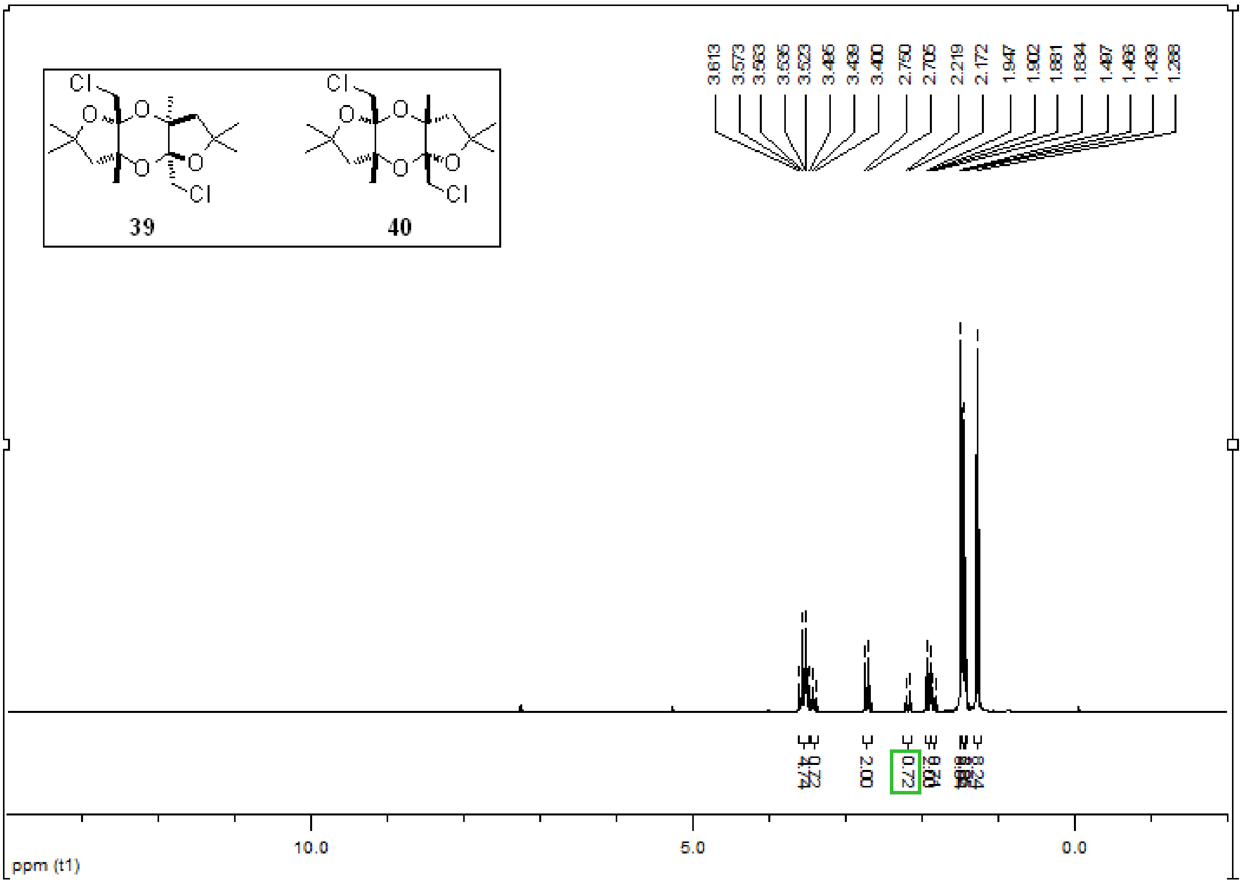

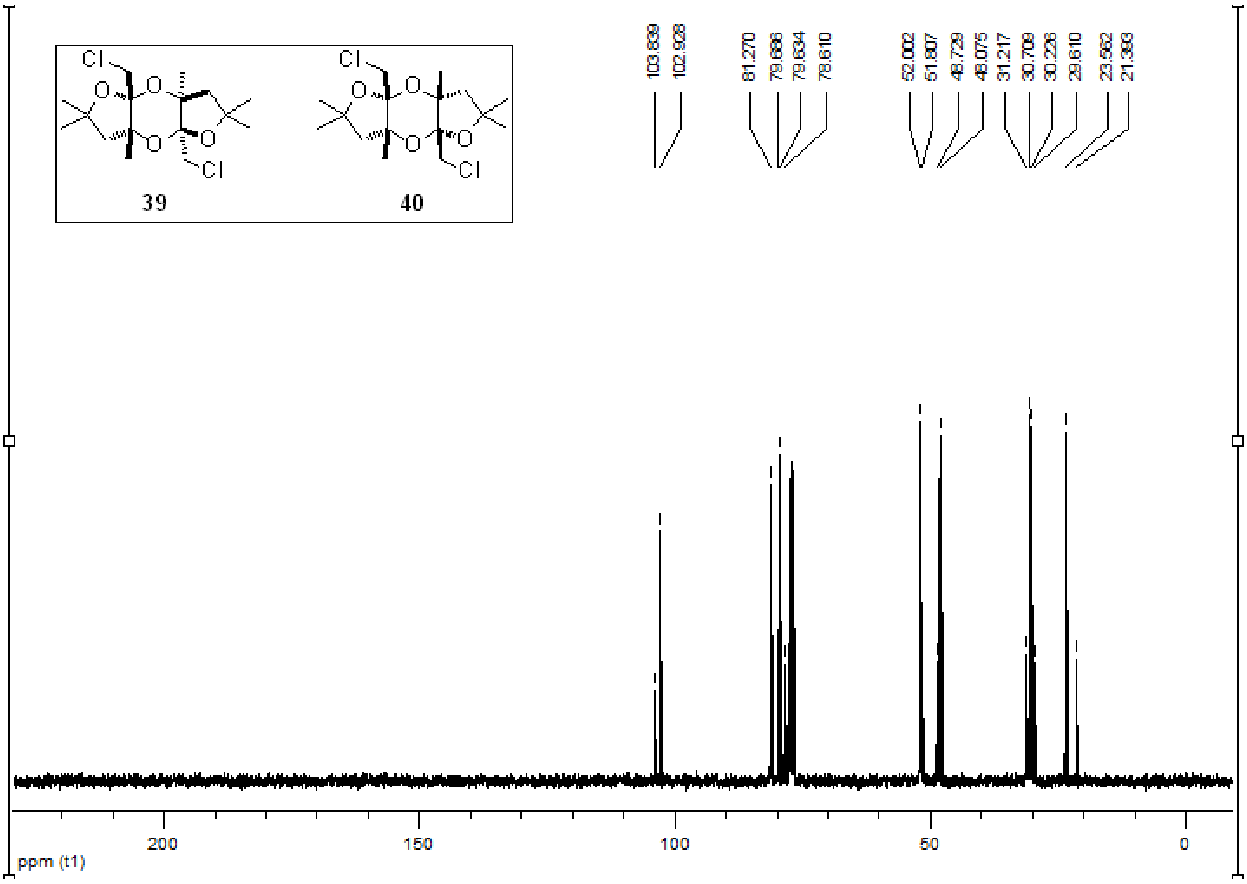

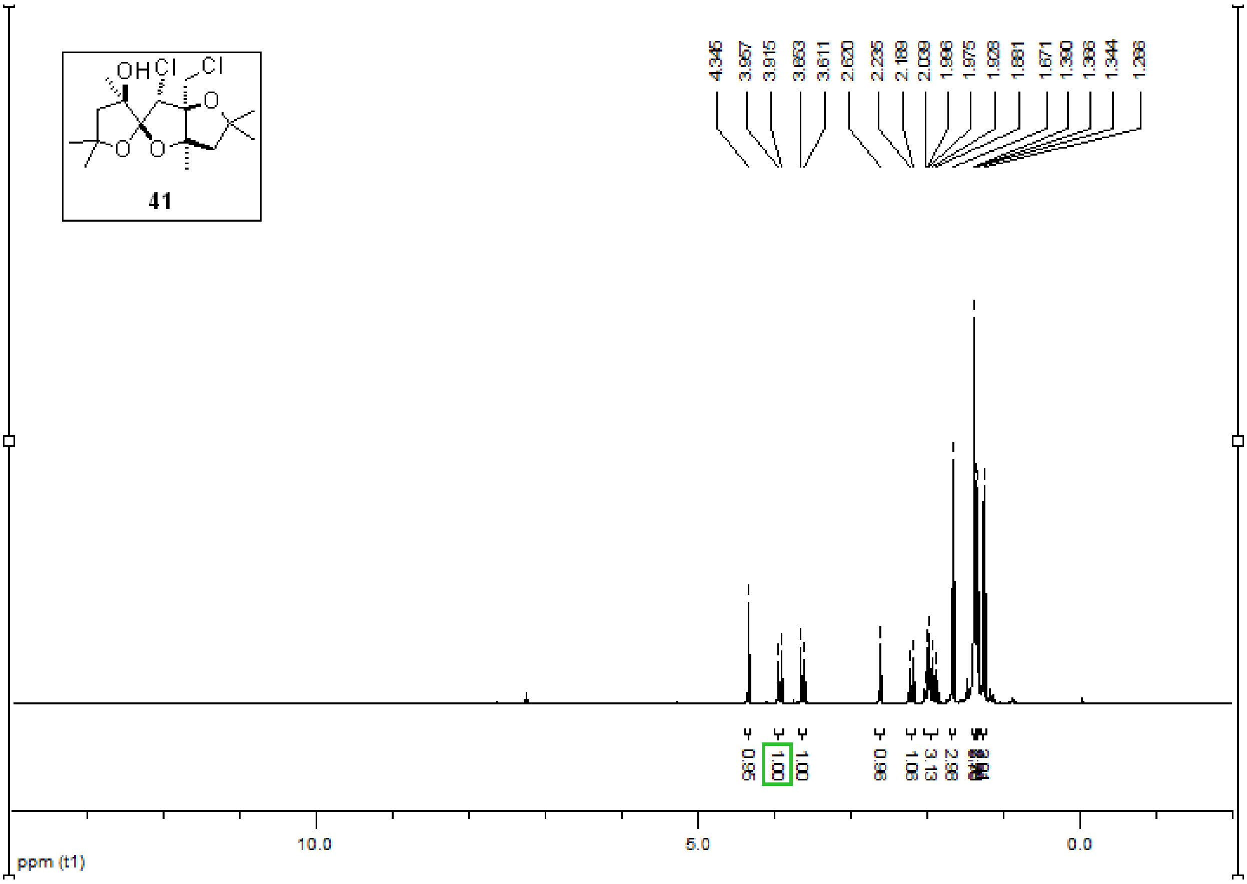

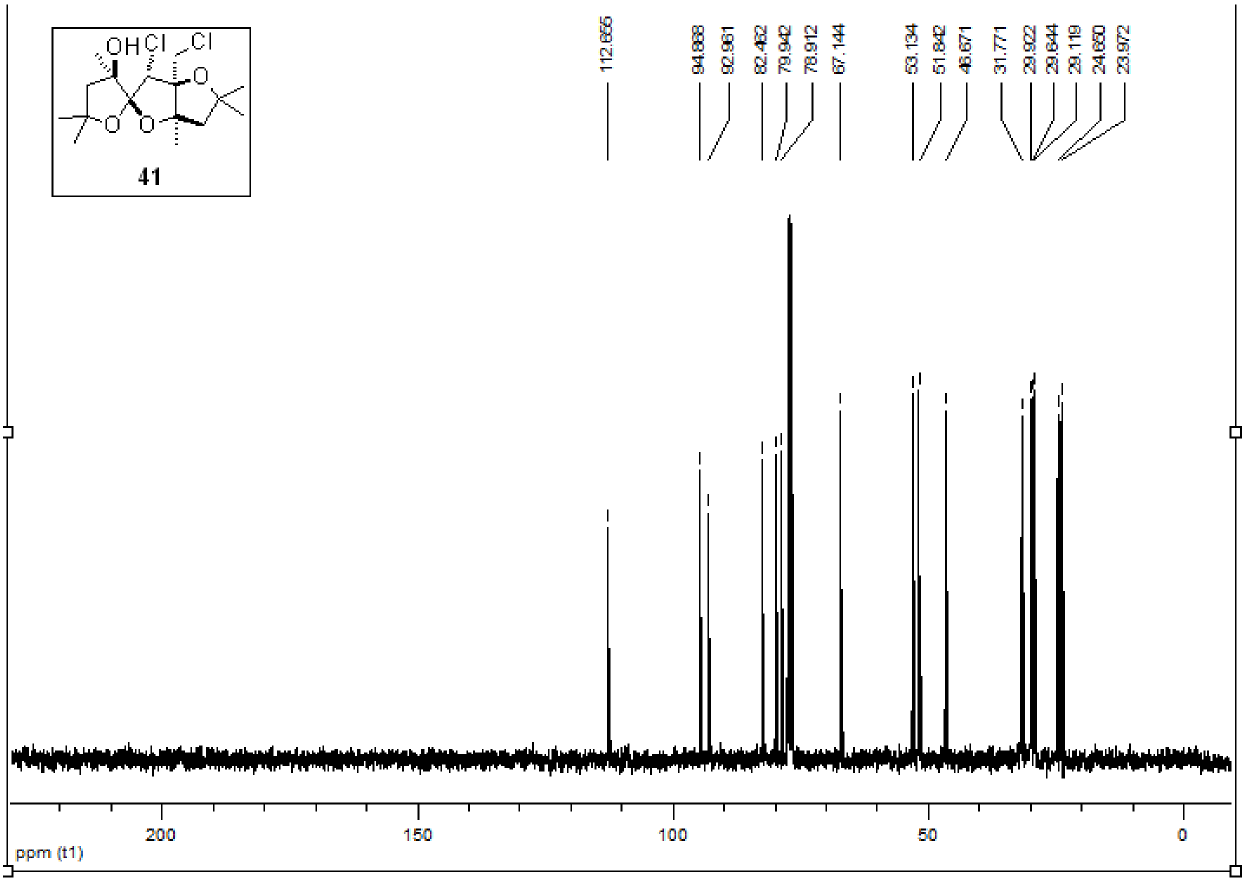

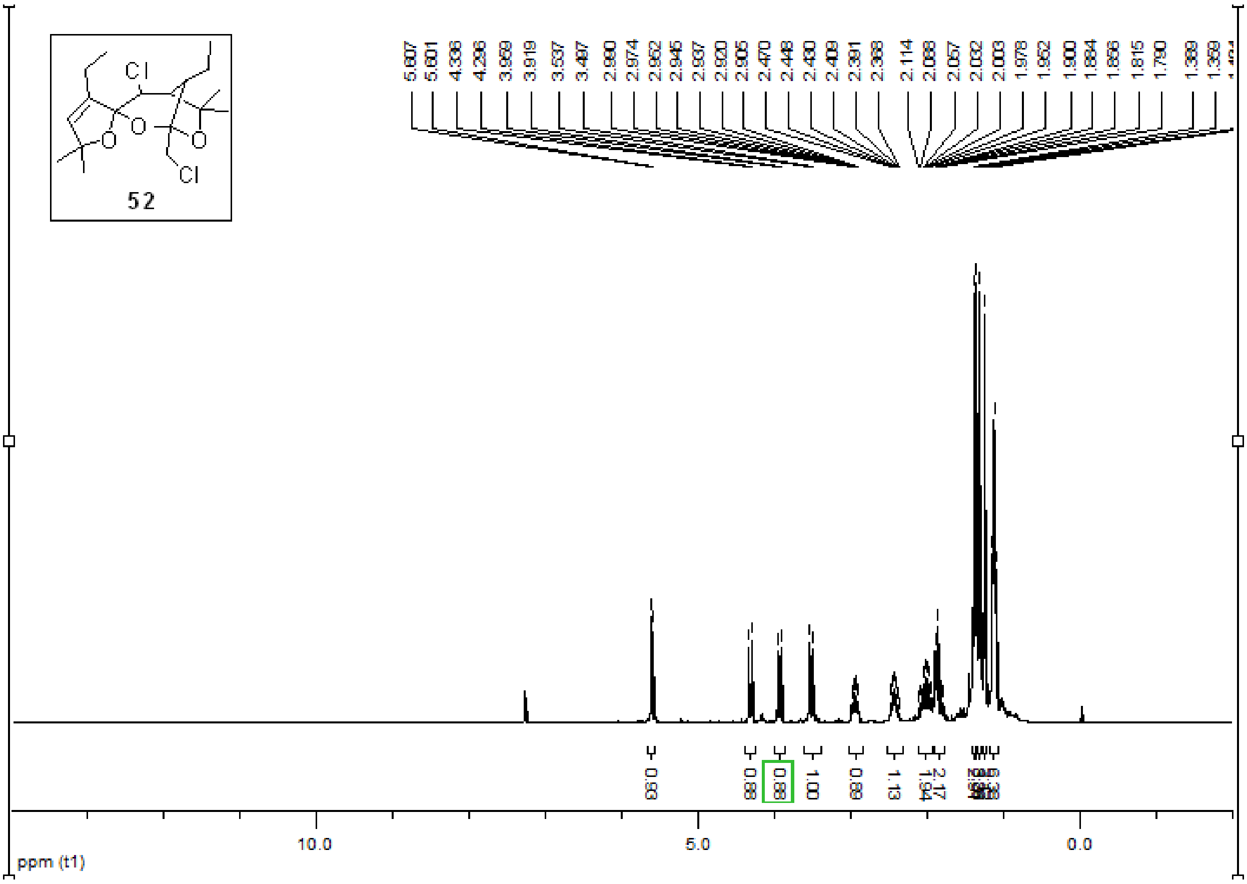
Cl
O
O
O
Cl
52
5.607
5.601
4.336
4.296
3.959
3.919
3.537
3.497
2.990
2.974
2.962
2.946
2.937
2.920
2.905
2.470
2.448
2.430
2.409
2.391
2.368
2.114
2.088
2.057
2.032
2.003
1.978
1.962
1.900
1.884
1.856
1.815
1.790
1.389
1.359
0.93
0.88
0.88
1.00
0.89
1.13
1.94
2.17
10.0
5.0
0.0
ppm (t1)

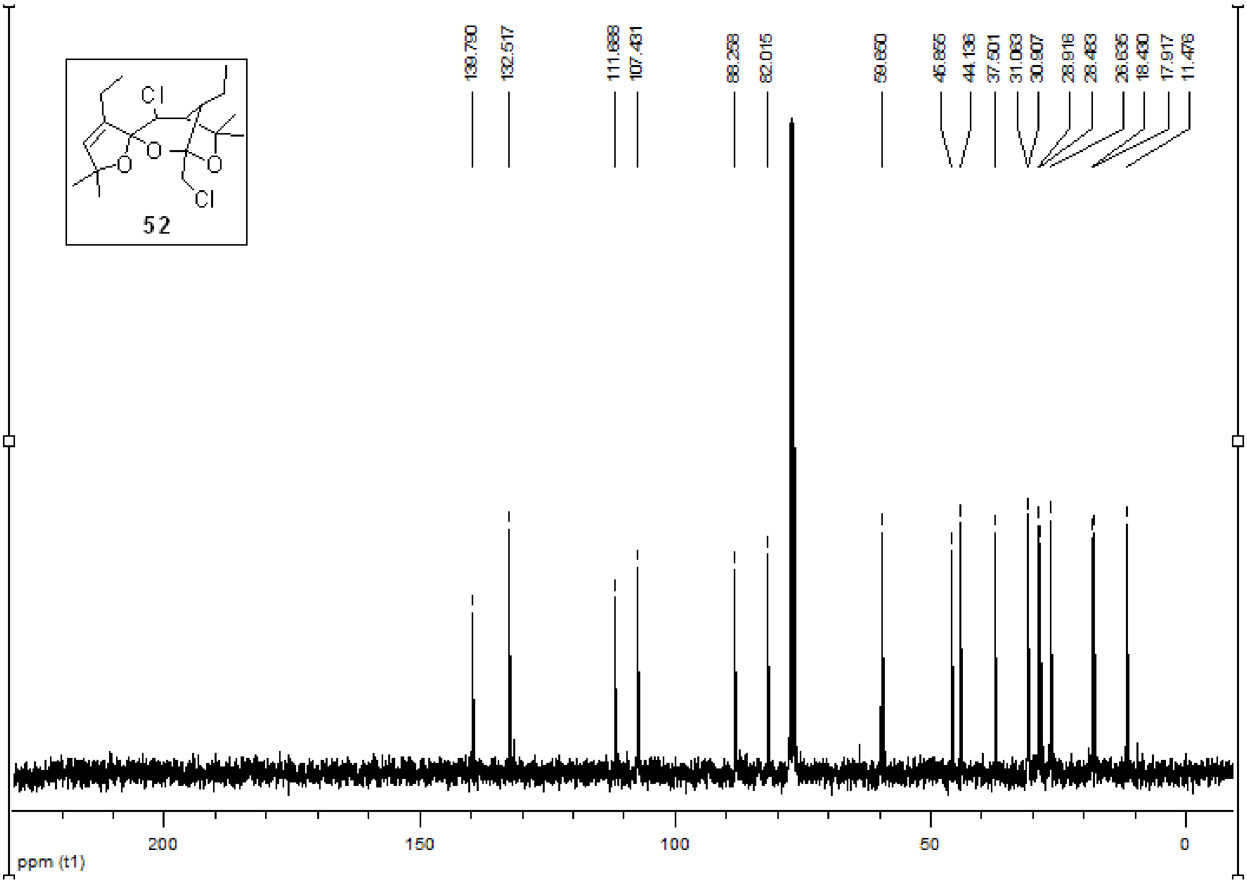
Cl
O
O
O
Cl
52
139.790
132.517
111.688
107.431
88.258
82.015
59.650
45.655
44.136
37.501
31.063
30.907
28.916
28.483
26.635
18.430
17.917
11.476
200
150
100
50
0
ppm (t1)

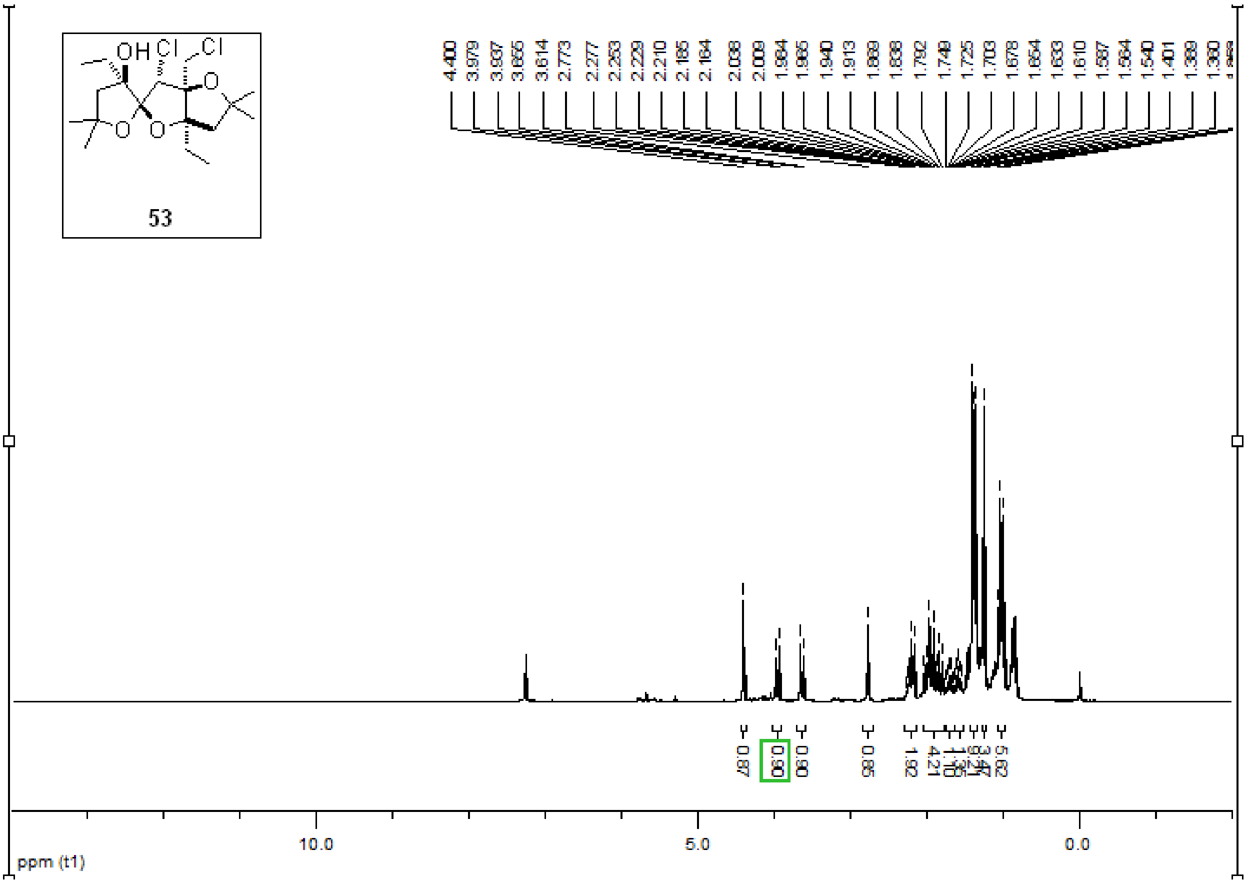

OH
Cl
Cl
O
O
O
53
4.400
3.979
3.957
3.655
3.614
2.773
2.277
2.253
2.229
2.210
2.185
2.164
2.038
2.008
1.984
1.965
1.940
1.913
1.889
1.868
1.792
1.749
1.725
1.703
1.678
1.654
1.633
1.610
1.587
1.564
1.540
1.401
1.389
1.380
0.87
0.90
0.90
0.85
1.92
4.21
1.10
10.0
5.0
0.0
ppm (t1)

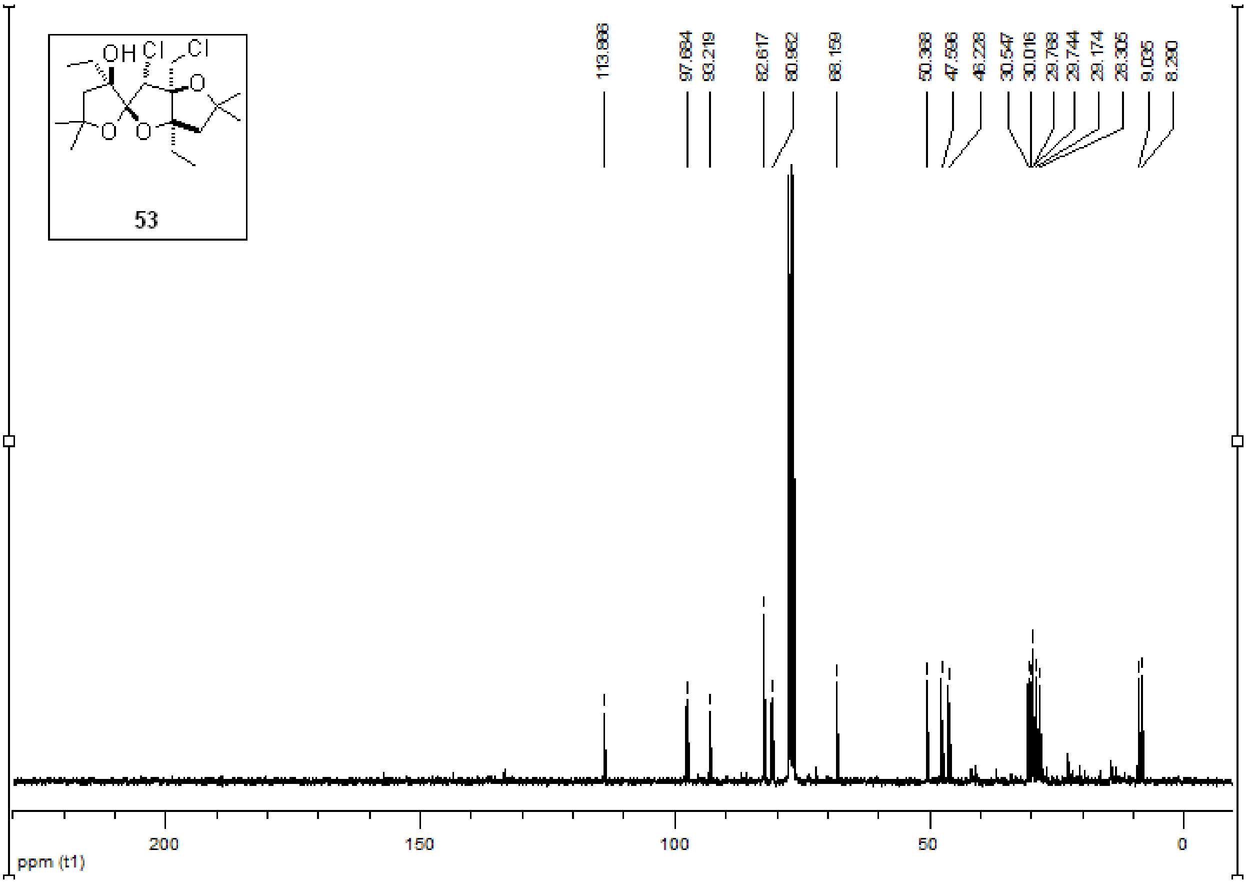

OH
Cl
Cl
O
O
O
53
113.996
97.684
93.219
82.617
80.962
68.159
50.368
47.596
46.228
30.547
30.016
29.768
29.744
29.174
28.305
9.035
8.290
200
150
100
50
0
ppm (t1)

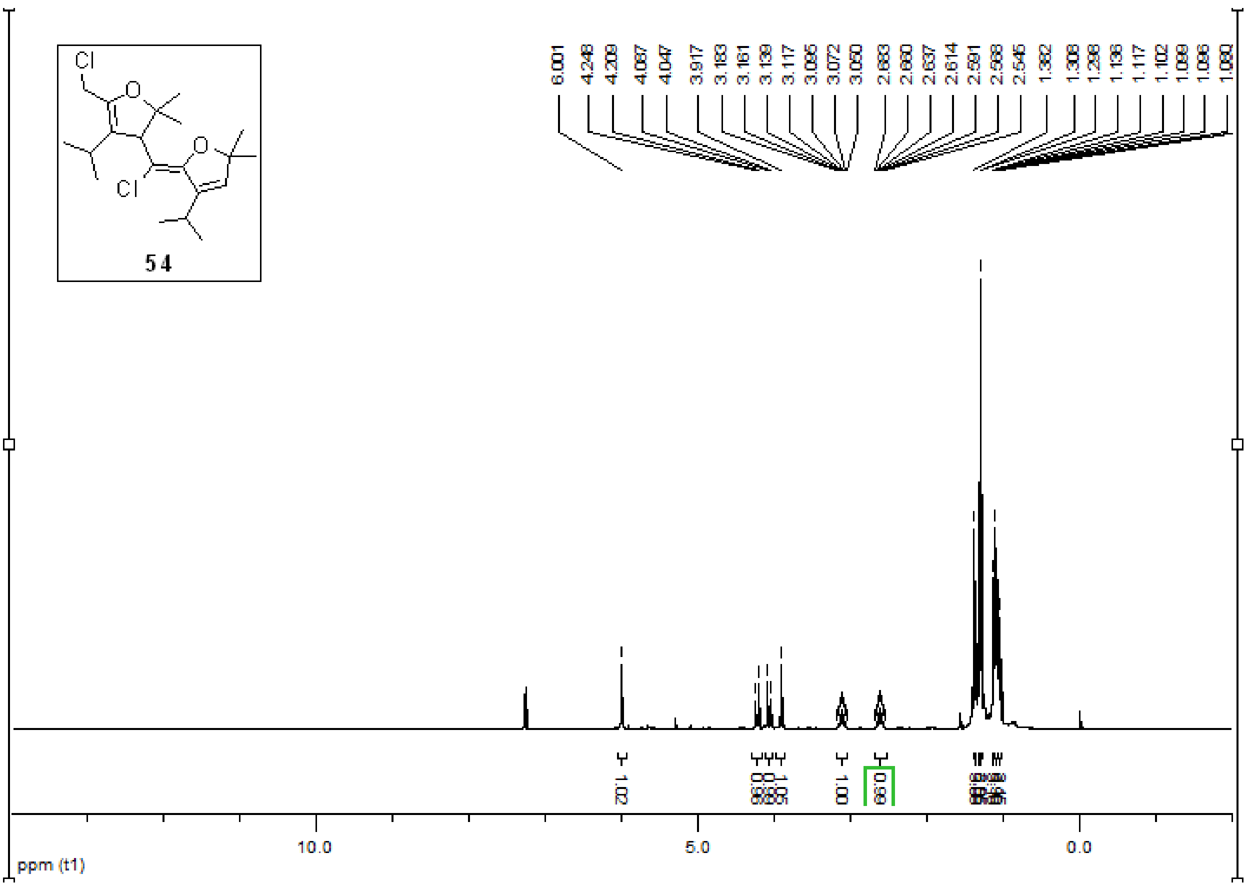
Cl
O
Cl
O
54
6.001
4.248
4.209
4.087
4.047
3.917
3.183
3.161
3.139
3.117
3.095
3.072
3.050
2.683
2.660
2.637
2.614
2.591
2.568
2.545
1.382
1.308
1.298
1.136
1.117
1.102
1.099
1.096
1.080
1.02
1.00
ppm (t1)
10.0
5.0
0.0

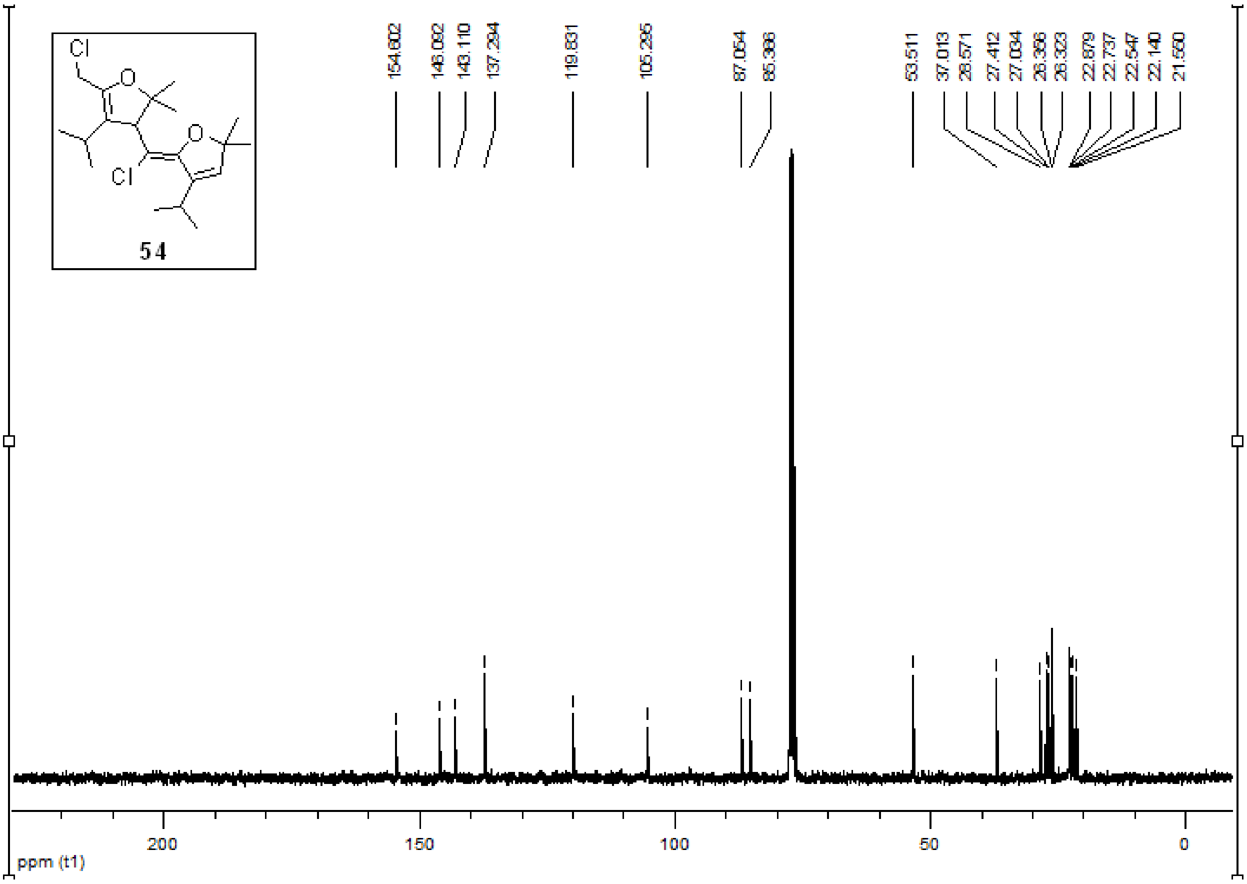
Cl
O
Cl
O
54
154.602
146.092
143.110
137.294
119.831
105.295
87.054
85.366
53.511
37.013
28.571
27.412
27.034
26.356
26.323
22.879
22.737
22.547
22.140
21.550
ppm (t1)
200
150
100
50
0

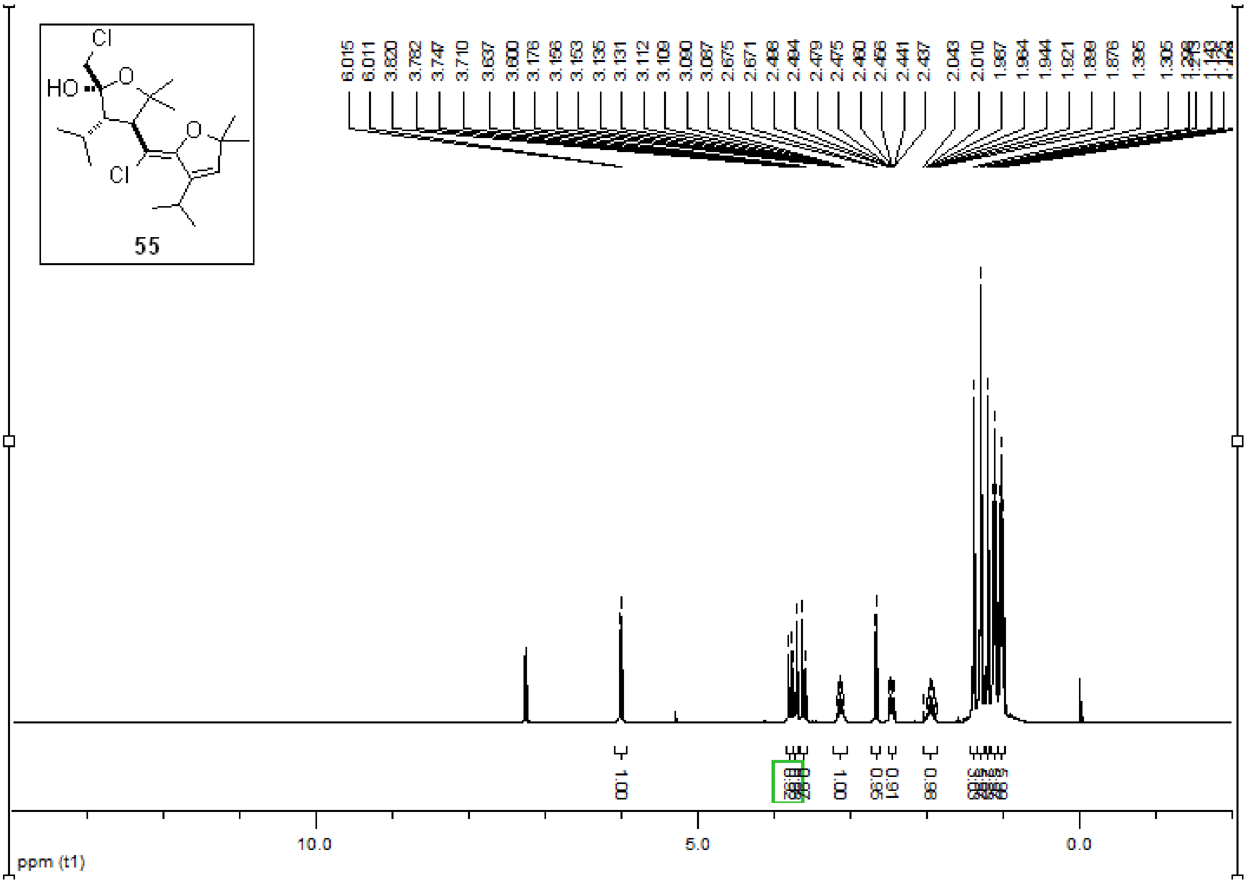
55
ppm (t1)
10.0
5.0
0.0

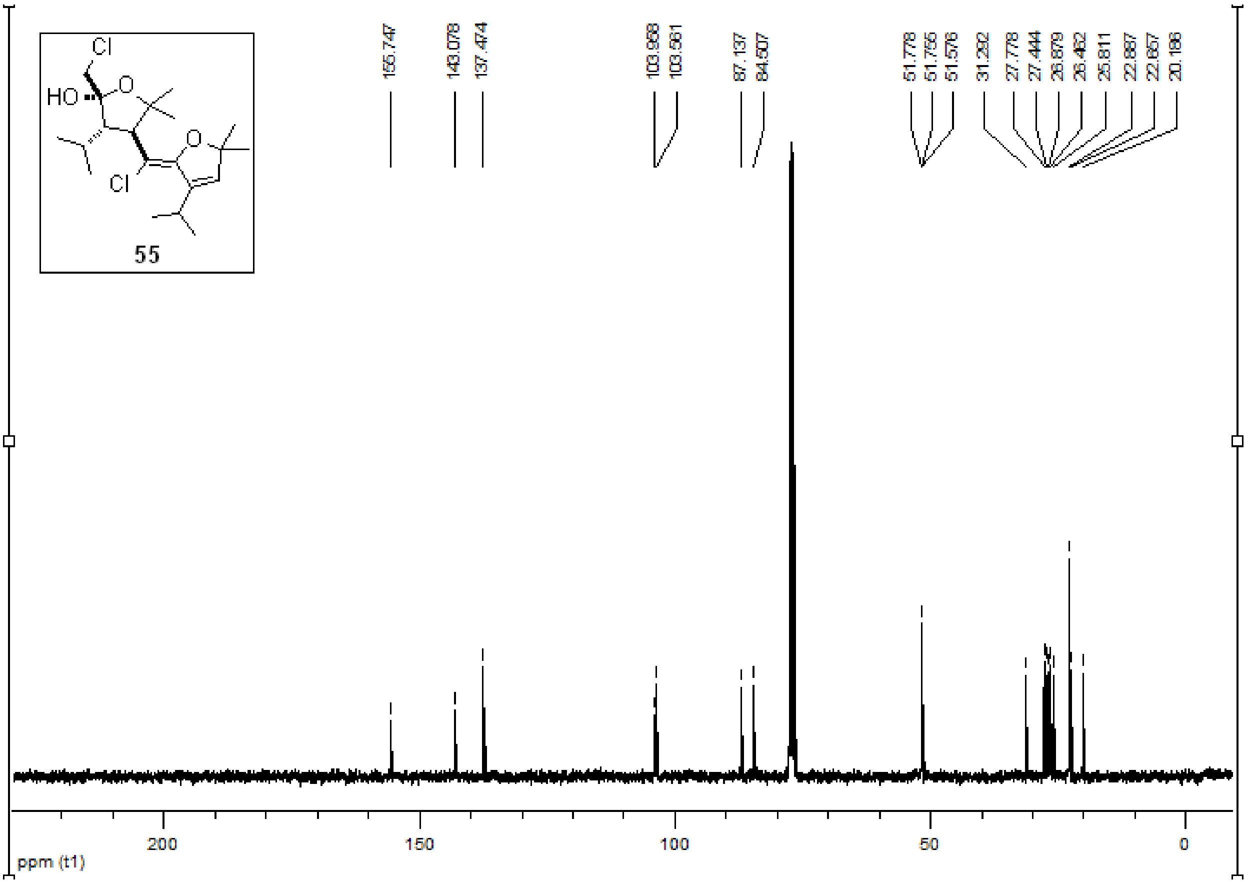
55
155.747
143.078
137.474
103.958
103.561
87.137
84.507
51.778
51.755
51.576
31.292
27.778
27.444
26.879
26.452
25.811
22.887
22.657
20.186
ppm (t1)
200
150
100
50
0

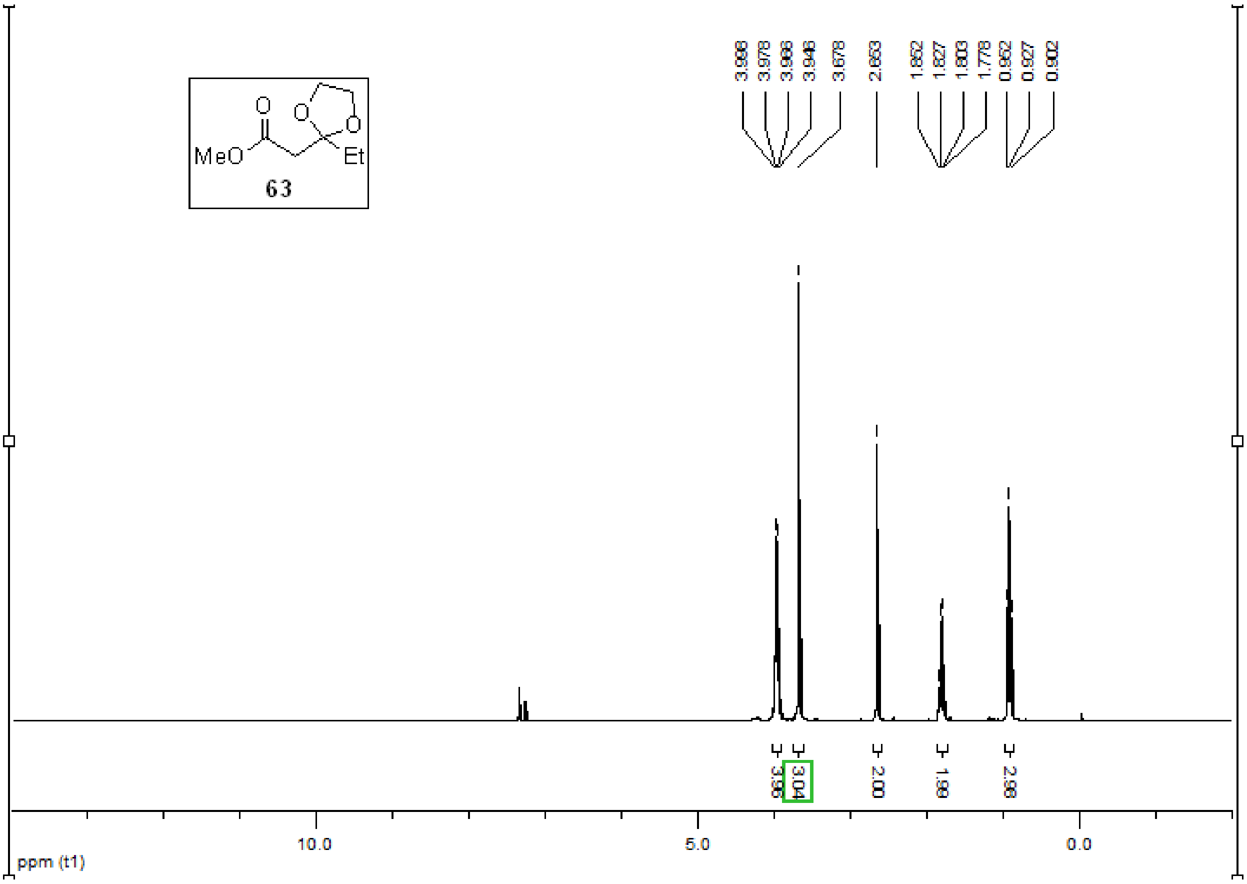

MeO
O
O
O
Et
63
3.998
3.978
3.966
3.946
3.678
2.653
1.852
1.827
1.803
1.778
0.952
0.927
0.902
3.95
3.04
2.00
1.99
2.96
10.0
5.0
0.0
ppm (t1)

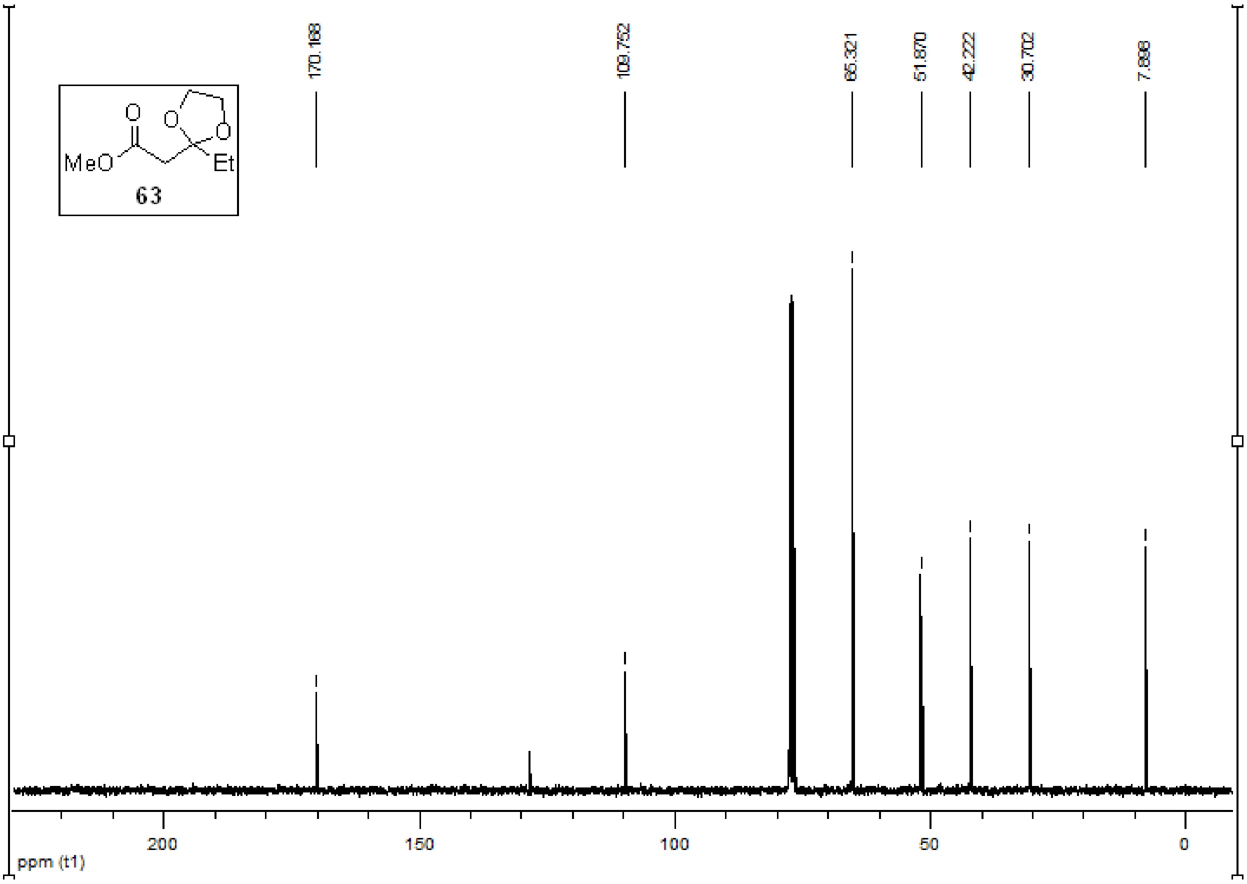

MeO
O
O
O
Et
63
170.168
109.752
65.321
51.870
42.222
30.702
7.898
200
150
100
50
0
ppm (t1)

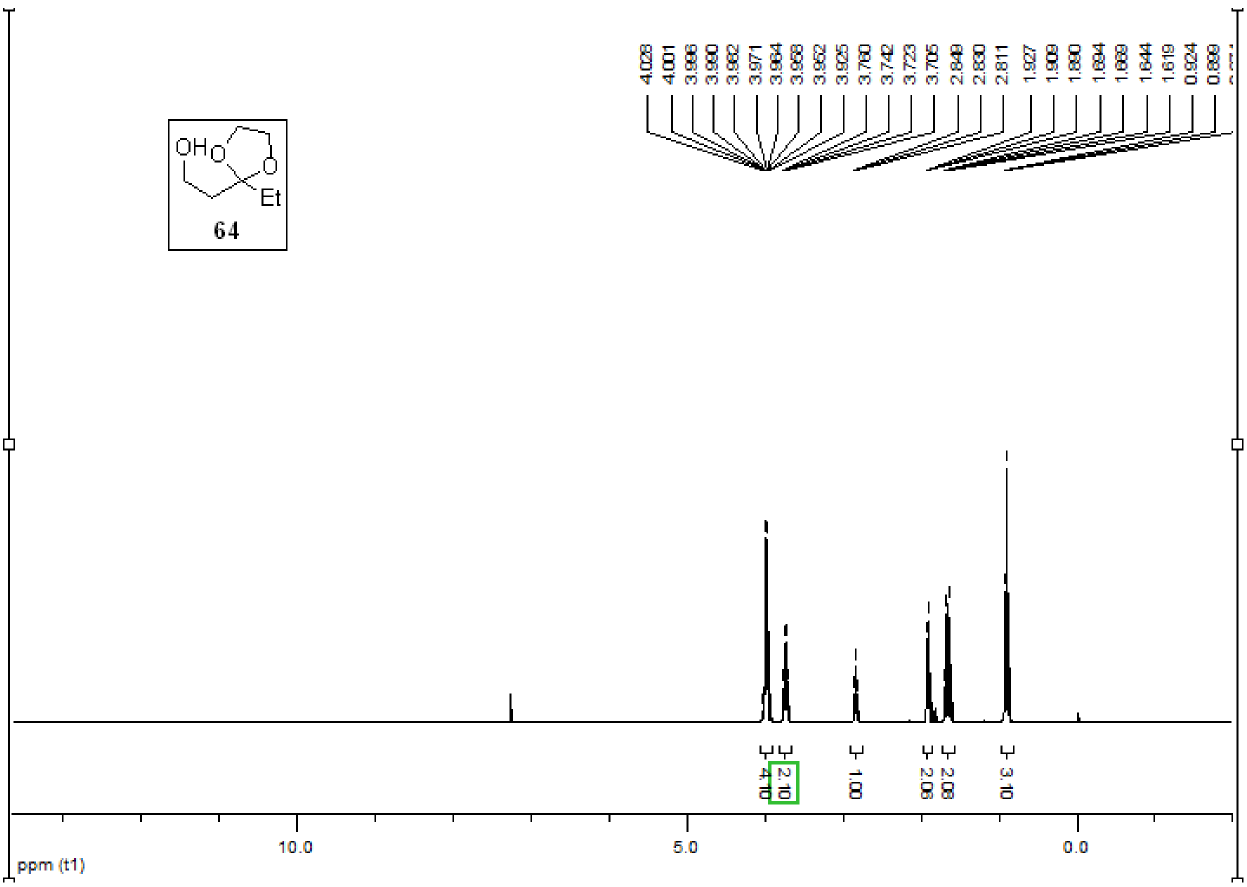

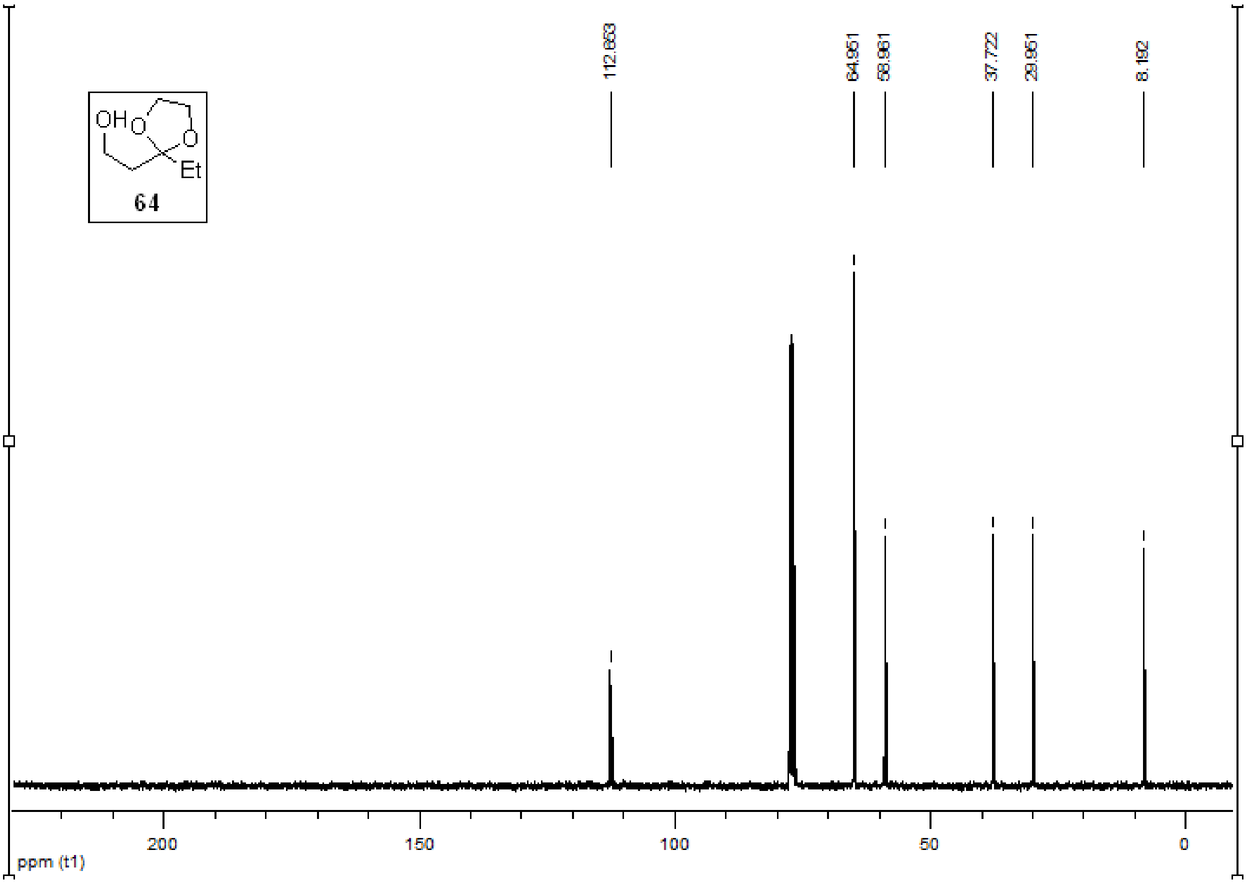

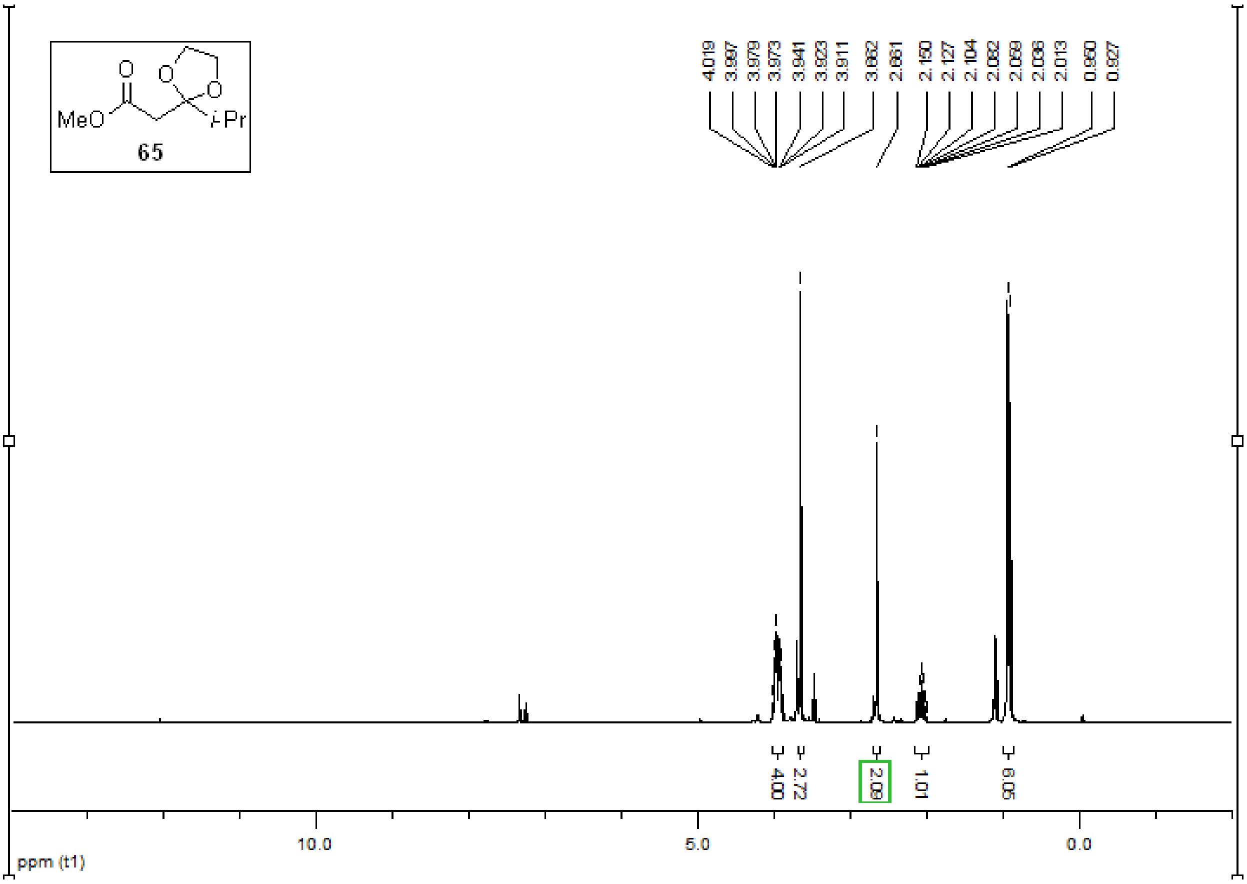
MeO
O
O
O
i-Pr
65
4.019
3.997
3.979
3.973
3.941
3.923
3.911
3.662
2.661
2.150
2.127
2.104
2.082
2.059
2.036
2.013
0.950
0.927
4.00
2.72
2.09
1.01
6.05
10.0
5.0
0.0
ppm (t1)

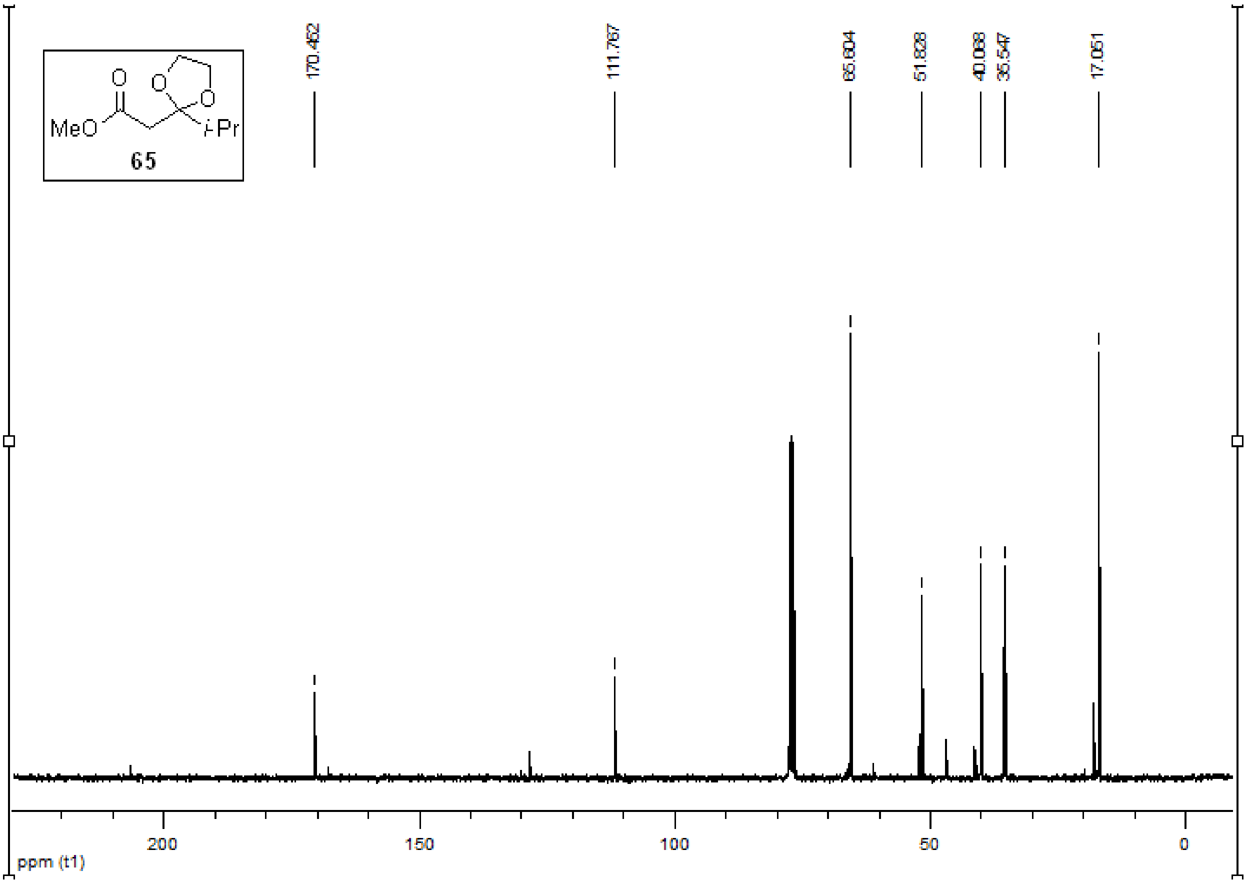
MeO
O
O
O
i-Pr
65
170.462
111.767
65.604
51.628
40.068
35.547
17.051
200
150
100
50
0
ppm (t1)

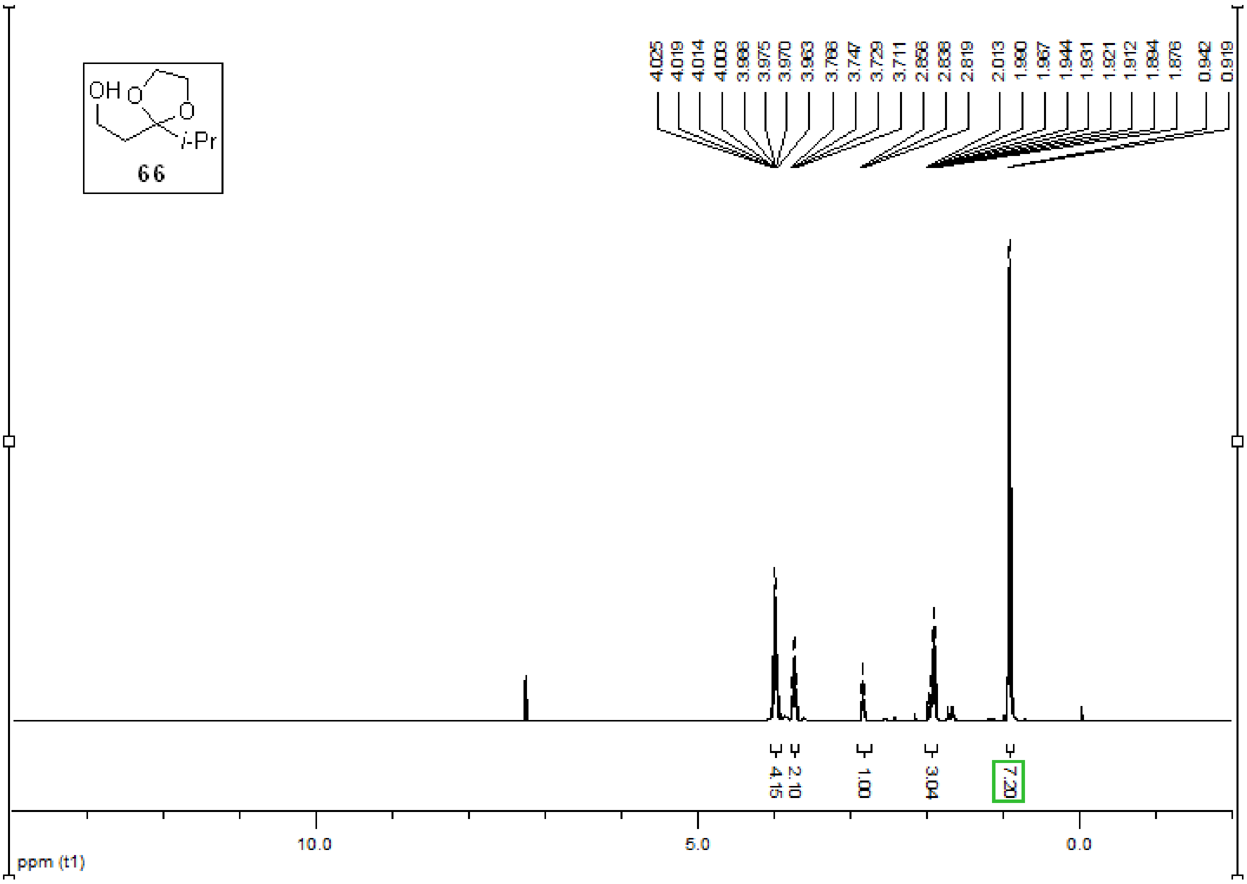

66
4.025
4.019
4.014
4.003
3.986
3.975
3.970
3.963
3.766
3.747
3.729
3.711
2.856
2.838
2.819
2.013
1.990
1.967
1.944
1.931
1.921
1.912
1.894
1.876
0.942
0.919
4.15
2.10
1.00
3.04
7.20
10.0
5.0
0.0
ppm (t1)

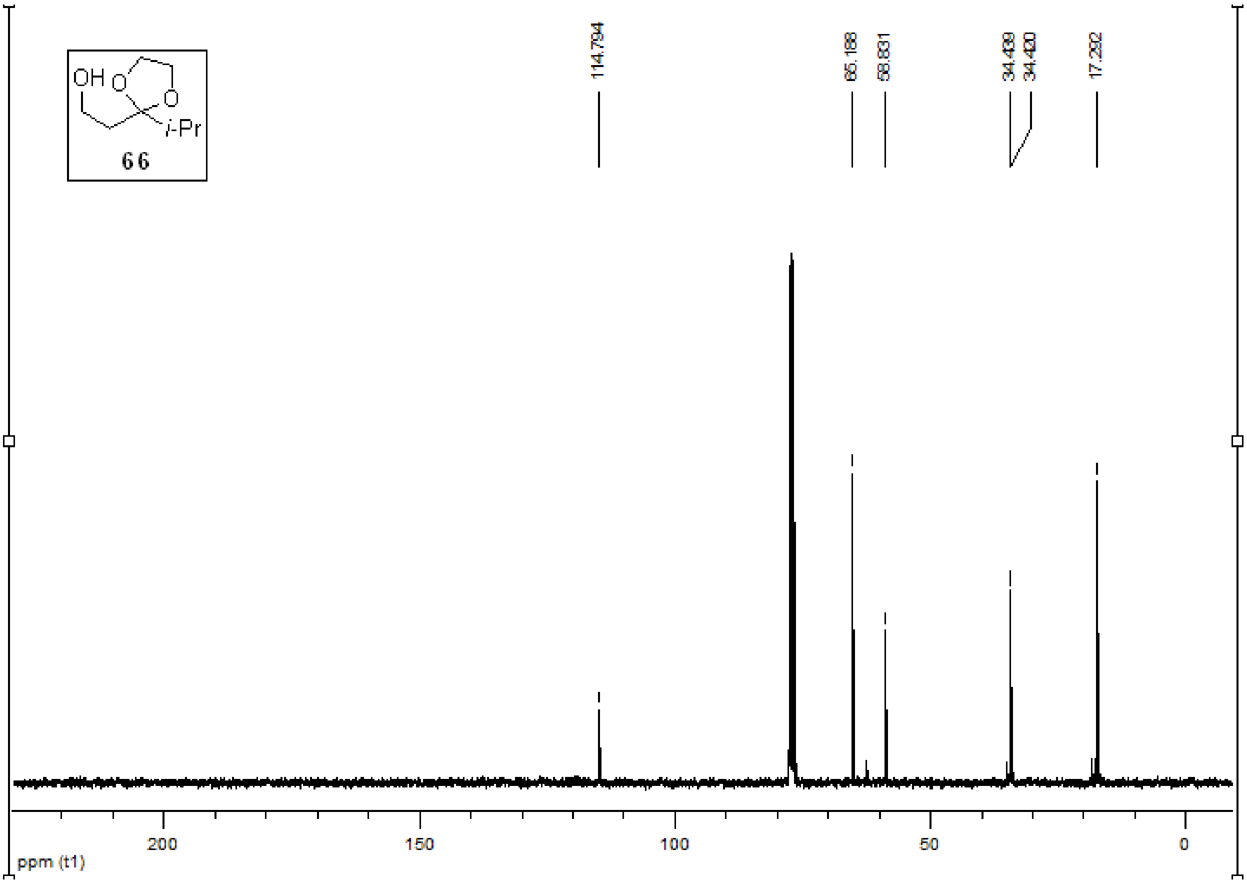

66
114.794
65.188
58.831
34.439
34.420
17.292
200
150
100
50
0
ppm (t1)

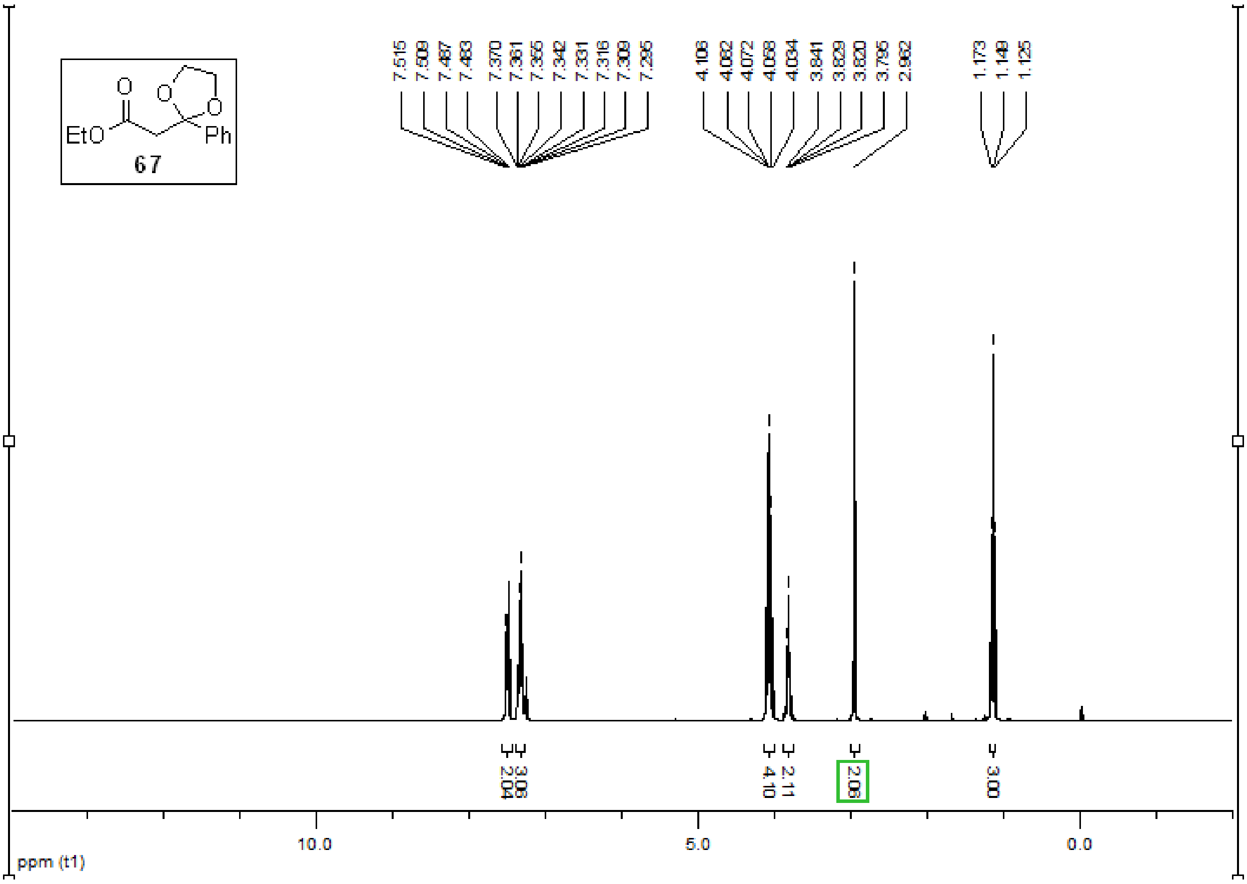
7.515
7.509
7.487
7.483
7.370
7.361
7.355
7.342
7.331
7.316
7.309
7.295
4.106
4.082
4.072
4.058
4.034
3.841
3.829
3.820
3.796
2.962
1.173
1.149
1.125
EtO
O
O
O
Ph
67
2.04
3.06
4.10
2.11
2.06
3.00
10.0
5.0
0.0
ppm (t1)

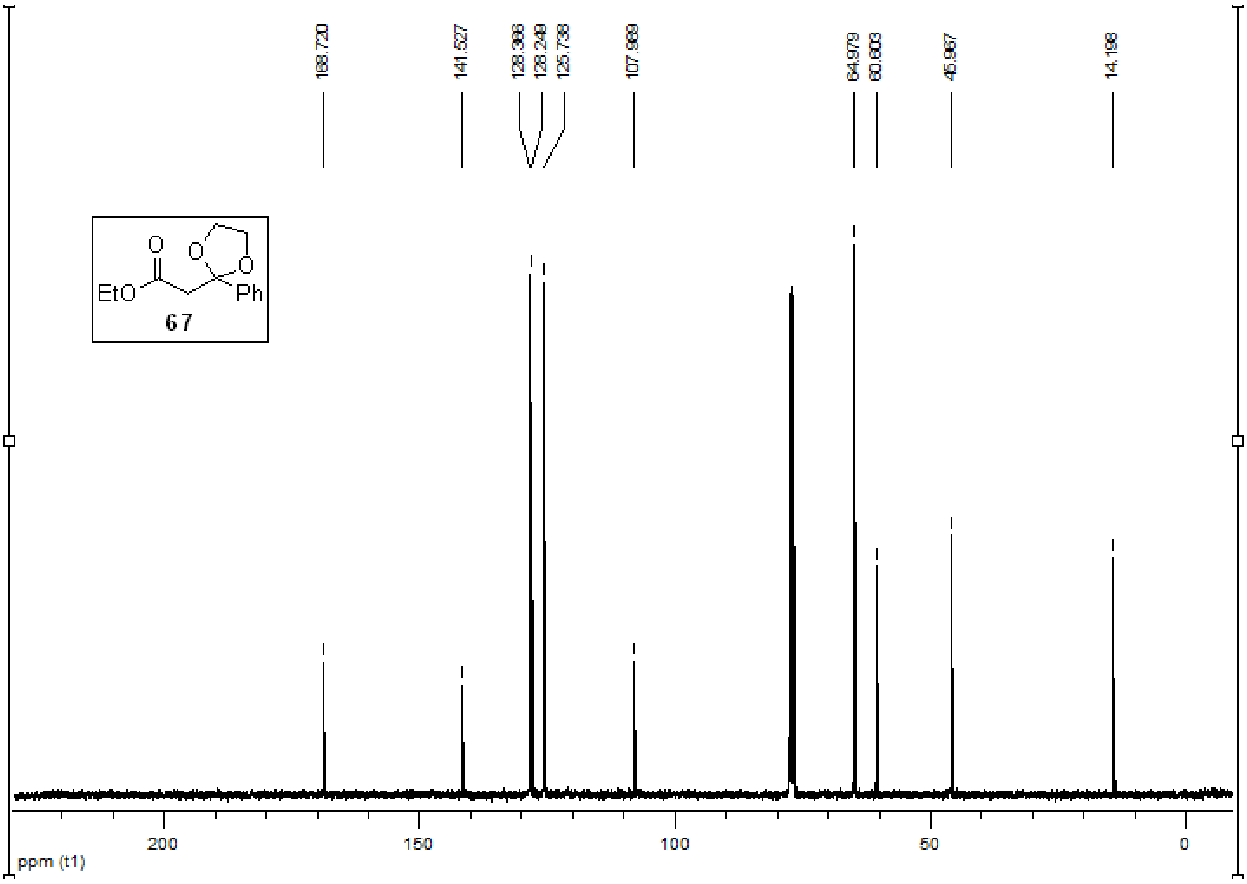
168.720
141.527
128.366
128.249
125.738
107.989
64.979
60.603
45.967
14.198
EtO
O
O
O
Ph
67
200
150
100
50
0
ppm (t1)

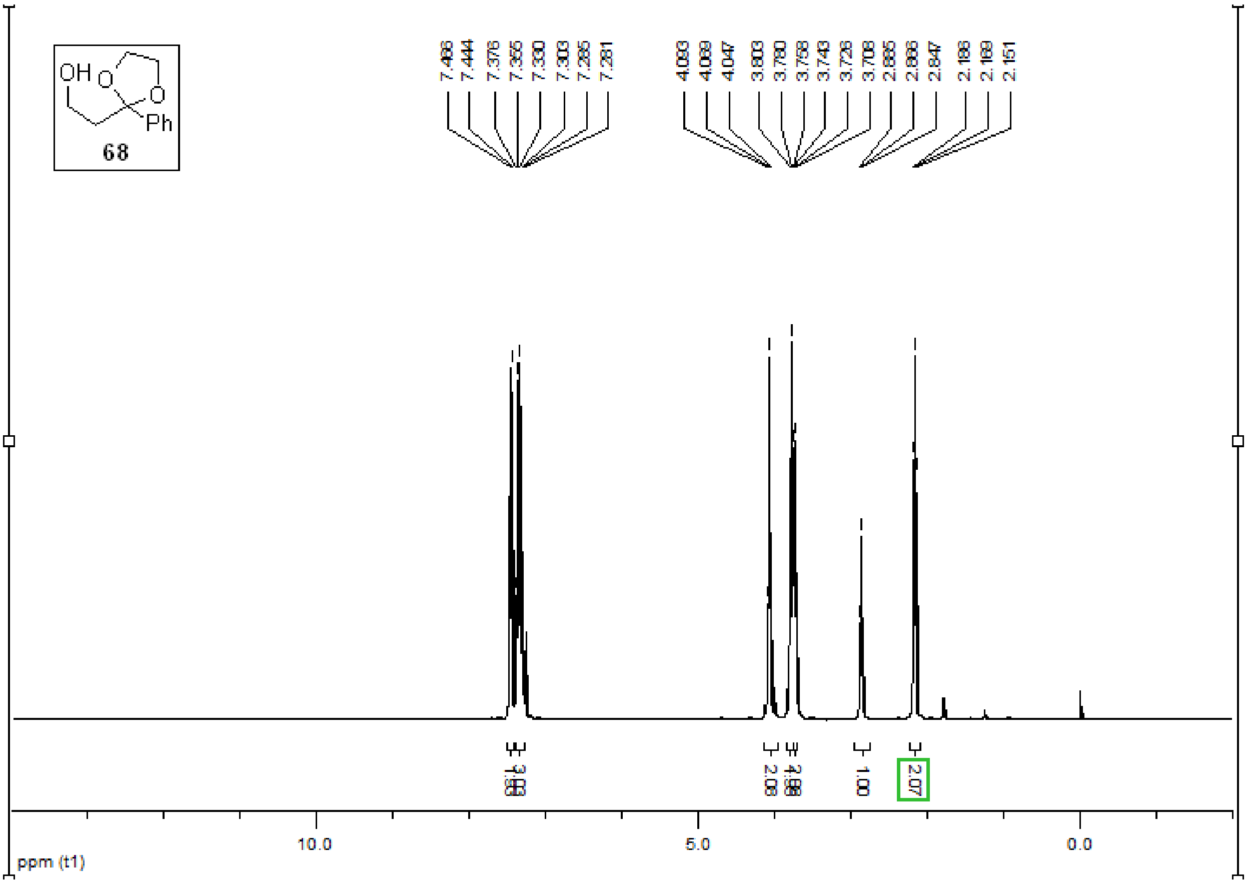

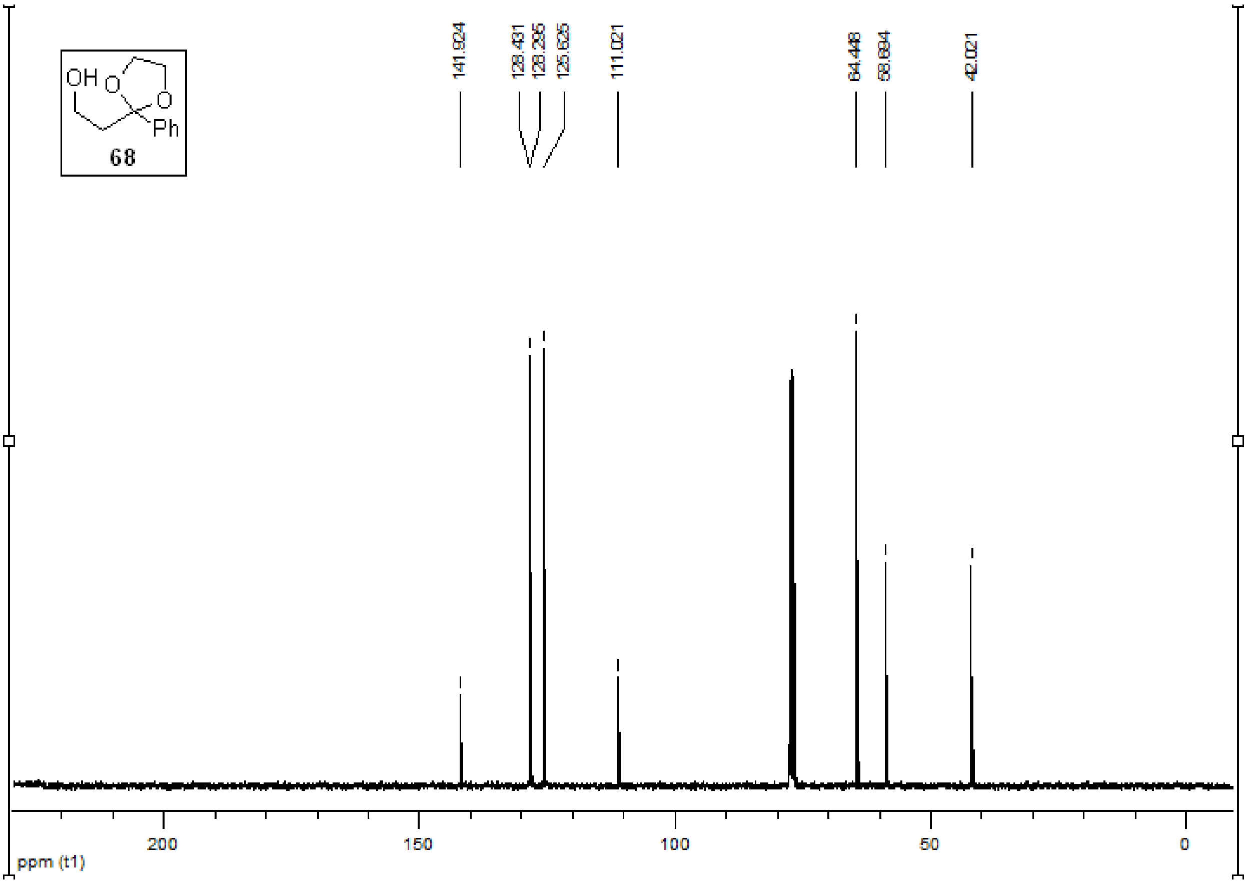

Supplement: File 1 — Experimental procedures and analytical data of cited compounds. [file Beilstein_J_Org_Chem-09-1319-s001.pdf]
